# Characterization of Glycoside Hydrolase Families 13 and 31 Reveals Expansion and Diversification of α-Amylase Genes in the Phlebotomine *Lutzomyia longipalpis* and Modulation of Sandfly Glycosidase Activities by *Leishmania* Infection

**DOI:** 10.3389/fphys.2021.635633

**Published:** 2021-04-09

**Authors:** Samara Graciane da Costa-Latgé, Paul Bates, Rod Dillon, Fernando Ariel Genta

**Affiliations:** ^1^Laboratory of Insect Biochemistry and Physiology, Oswaldo Cruz Institute, Fundação Oswaldo Cruz, Rio de Janeiro, Brazil; ^2^Faculty of Health and Medicine, Division of Biomedical and Life Sciences, Lancaster University, Lancaster, United Kingdom; ^3^National Institute of Science and Technology for Molecular Entomology, Rio de Janeiro, Brazil

**Keywords:** *Lutzomyia longipalpis*, sugar, sucrase, α-glucosidase, α-amylase, glycoside hydrolase, *Leishmania mexicana*

## Abstract

Sugar-rich food sources are essential for sandflies to meet their energy demands, achieving more prolonged survival. The digestion of carbohydrates from food is mainly realized by glycoside hydrolases (GH). To identify genes coding for α-glycosidases and α-amylases belonging to Glycoside Hydrolase Family 13 (GH13) and Glycoside Hydrolase Family 31 (GH31) in *Lutzomyia longipalpis*, we performed an HMMER search against its genome using known sequences from other dipteran species. The sequences retrieved were classified based on BLASTP best hit, analysis of conserved regions by alignment with sequences of proteins with known structure, and phylogenetic analysis comparing with orthologous proteins from other dipteran species. Using RT-PCR analysis, we evaluated the expression of GH13 and GH31 genes, in the gut and rest of the body of females, in four different conditions: non-fed, sugar-fed, blood-fed, and *Leishmania mexicana* infected females. *L. longipalpis* has GH13/31 genes that code for enzymes involved in various aspects of sugar metabolism, as carbohydrate digestion, storage, and mobilization of glycogen reserves, proteins involved in transport, control of N-glycosylation quality, as well as others with a putative function in the regulation of myogenesis. These proteins are representatives of GH13 and GH31 families, and their roles seem to be conserved. Most of the enzymes seem to be active with conserved consense sequences, including the expected catalytic residues. α-amylases also demonstrated the presence of calcium and chloride binding sites. *L. longipalpis* genome shows an expansion in the α-amylase gene family, with two clusters. In contrast, a retraction in the number of α-glucosidases occurred. The expansion of α-amylases is probably related to the specialization of these proteins for different substrates or inhibitors, which might correlate with the higher diversity of plant foods available in the natural habitat of *L. longipalpis*. The expression of α-glucosidase genes is higher in blood-fed females, suggesting their role in blood digestion. Besides that, in blood-fed females infected with the parasite *Leishmania mexicana*, these genes were also modulated. Glycoside Hydrolases from families 13 and 31 are essential for the metabolism of *L. longipalpis*, and GH13 enzymes seem to be involved in the interaction between sandflies and *Leishmania*.

## Introduction

For adult sandflies, the energy requirements are provided primarily by sugar meals obtained from different sources, for both females and males. These sugar sources have been described for different sandfly species, depending on their preferred site for development. Desert and savanna regions are the preferred habitats for the genus *Phlebotomus* (Old World) and tropical forests for the genus *Lutzomyia* (New World). Sandflies commonly feed on plant tissues, fruit juices, nectars from flowers, honeydews excreted by aphids and coccids, among other sources, with different sugar compositions (Killick-Kendrick and Killick-Kendrick, 1987; Macvicker et al., 1990; Cameron et al., 1995). For females feeding on blood for egg maturation, meal is also rich in sugar and glyco-derivatives, as glycoproteins and glycolipids. The immature forms of sandflies are detritivores. Although there is no definitive proof, they develop by ingesting soil rich in bacteria, fungi, and organic molecules derived from decomposition of plant and animal cells (Moraes et al., 2012; Vale et al., 2012). Thus, glycosidases play a crucial role in the digestion of larvae and adult sandflies.

Alpha-amylases and α-glucosidases are vital for sand fly adults since their substrates are common components of plant tissues and secretions. Starch (substrate for amylase) occurs in leaves and fruits, and sucrose (substrate for α-glucosidase) is the main component of sap and nectar, and, besides other sugars, is also found in honeydew (Killick-Kendrick and Killick-Kendrick, 1987; Macvicker et al., 1990). In larvae, these enzymes may play an essential role in glycogen digestion, the reserve carbohydrate in fungi (Moraes et al., 2012). Based on similarity, gene sequences of these enzymes have been grouped into families. In insects, these enzymes are described in glycoside hydrolases families 13 (GH13) and 31 (GH31) (Cantarel et al., 2009).

Alpha-amylases play an important role in the breakdown of internal α-1,4-glucan bonds of starch and related polysaccharides, such as amylose and amylopectin (Janeček et al., 2014). They are soluble enzymes participating in the process of initial digestion in insects. GH13 α-glucosidases are exoglycosidases acting generally upon α-glucosidic bonds, including α-1,4 bonds, and participate in hydrolysis of aryl glycosides, disaccharides and oligosaccharides with varying efficiency (Terra and Ferreira, 1994). GH31 α-glucosidases are intracellular proteins participating in the glycogen metabolism and glycoconjugate biosynthesis and processing (Lovering et al., 2005).

In insects, the glucose obtained after hydrolysis of sugar-rich food is uptaken by enterocytes through specific membrane transporters. Then, glucose is used to produce energy through glycolysis and related metabolic pathways or converted to trehalose, the circulating sugar in the hemolymph. When circulating trehalose is in excess, glucose is stored as glycogen or converted into triacyl-glycerides in several tissues. Insects have to expend energy continuously, and adaptation to food availability changes is a central challenge for survival. In a starving condition, they must live on reserves (glycogen or triacyl-glycerides) accumulated in periods of food abundance. The flight, for example, is a condition of extreme energy demand (Thompson, 2003; Arrese and Soulages, 2010). In insects, some of the enzymes involved in the storage and mobilization of glycogen, as the 1,4-α-glucan branching enzyme, glycogen debranching enzyme (GDE), and lysosomal α-glucosidase are also members of GH13 or GH31 families.

The GH13 is the largest family of glycoside hydrolases, presenting a wide variety of enzymes with more than 30 different specificities. These enzymes may act on several substrates, by hydrolyzing α-glycosidic bonds, generating α-anomeric mono/oligosaccharides, or catalyzing α-glycosidic bond formation by transglycosylation (Kuriki and Imanaka, 1999). GH13 family includes representatives of α-amylases, α-glucosidases, α-1,4-glucan branching enzymes, pullulanases, cyclodextrin glucanotransferases, 4-α-glucanotransferases (the domain of glycogen debranching enzyme), oligo-α-1,6-glucosidases, amino acid transporters, besides others.

The GH31 is also a diverse group, with a range of different hydrolytic activities. Members of this family are capable of cleaving a terminal carbohydrate moiety from substrates varying considerably in size, from disaccharides to complex reserve polysaccharides such as starch, glycogen, or glycoproteins (Ernst et al., 2006). α-glucosidases, α-xylosidases, α-galactosidases, α-mannosidases, 6-α-glucosyltransferases, and 3-α-isomaltosyltransferases, besides others, represent the hydrolytic activities that are present in the GH31 family. The best-described activities in this group are the α-glucosidases that are involved in the glycogen degradation (lysosomal α-glucosidase) (Hoefsloot et al., 1988), and in glycoprotein processing (neutral α-glucosidase) in the endoplasmic reticulum (Trombetta et al., 1996, 2001).

Enzymes classified in the GH13 or GH31 families share a typical tertiary structure in their catalytic domain, the (β/α)_8_-barrel fold. Members from the GH13 family present a conserved central nucleus composed by the catalytic domain A, domain B, which is a variable-length loop located between sheet β3 and α3 helix of the (β/α)_8_-barrel, and domain C, a Greek key motif in the C-terminal position (Ramasubbu et al., 1996; Janecek, 1997; Kanai et al., 2001; Abad et al., 2002). The conserved regions (CSRs) described in sheets β2, β3, β4, β5, β7, β8 and in the loop 3, were defined to help in the assignment of the correct enzymatic specificity to the members of this protein family. The catalytic triad aspartate, glutamate, and aspartate are located in the β4, β5, and β7 sheets, respectively (Janecek, 1997). Also, a fourth conserved residue was determined as an arginine positioned two residues preceding the catalytic nucleophile (MacGregor et al., 2001). In TAKA-amylase A from *Aspergillus oryzae* (Matsuura et al., 1984), one of the first GH13 structures to be elucidated, the Asp206 inside the GLRID domain (β4) acts as a catalytic nucleophile and Glu230 (β5) as the general acid/base. A second aspartate (Asp297, β7) contributes to stabilizing the transition state of the substrate during catalysis. It also maintains the glutamate with the correct protonation state for activity (Matsuura et al., 1984; Uitdehaag et al., 1999; Svensson and Macgregor, 2001). Insects α-amylases are typically calcium-dependent, and chloride may also works as an activator (Terra and Ferreira, 2012). The calcium-binding site has one aspartate residue in the loop 3 region, and asparagine and histidine positioned at the β3 and β4 sheets, respectively (Boel et al., 1990; Machius et al., 1995; Janecek, 1997). The chloride-binding site has a conserved arginine residue preceding the catalytic nucleophile, one asparagine residue placed two residues prior to the catalytic aspartate in the β7 sheet, and an Arg/Lys residue localized inside the variable domain RVMSSY (Janecek, 1997; D’Amico et al., 2000).

In the GH31 family, catalytic residues consist in one aspartate (catalytic nucleophile) inside the conserved domain WIDMNE (β4 sheet), and a second aspartate in the β6 sheet (general acid/base residue) (Lee et al., 2001; Lovering et al., 2005; Ernst et al., 2006; Sim et al., 2008). The sequence similarity is not evident between GH13 and GH31 members, but both families present a conserved aspartate as a catalytic nucleophile in the β4 sheet (Janeček et al., 2007). Most members of the GH31 family are multi-domain proteins, but the specific function of the accessory domains is generally unknown.

Phlebotomine sandflies are considered of medical importance, mainly because about 10% of their known species are vectors of some pathogen-caused disease (Akhoundi et al., 2016). Among the diseases caused by pathogens transmitted by phlebotomine sandflies, we can highlight the leishmaniasis. These diseases are caused by protozoan parasites belonging to the genus *Leishmania*, transmitted through the bite of female sandflies of the genus *Phlebotomus* in the Old World, and *Lutzomyia* in the New World (Killick-Kendrick, 1999; Sharma and Singh, 2008; Pace, 2014). In the Americas, *Lutzomyia longipalpis* is the natural vector of *Leishmania infantum*, the causative agent of visceral leishmaniasis, the most severe form of the disease (Soares and Turco, 2003; Murray et al., 2005; Akhoundi et al., 2016). In the insect vector, the *Leishmania* develops exclusively inside the gut. It is in direct contact with the digestive enzymes secreted by phlebotomines, especially proteases but also glycosidases, as alpha-amylases and alpha-glucosidases from GH13. *L. longipalpis* is a permissive vector, supporting the development of different *Leishmania* species as *L. mexicana*, *L. tropica*, *L. major*, and *L. amazonensis*, besides *L. infantum* (Soares and Turco, 2003; Kamhawi, 2006). *L. longipalpis* permissivity allows the study under laboratory conditions of the interaction of different *Leishmania* species with the digestive system of this vector.

Recently, we demonstrated the presence of at least four different α-glucosidase activities in *L. longipalpis*, with different specificities and putative roles in the digestion of plant or blood sugars (da Costa et al., 2019). However, the molecular identities of these enzymes are still unknown. In an initial effort to clarify this question, we revealed the presence of genes coding for proteins belonging to GH13 and GH31 in *L. longipalpis*. We compared *L. longipalpis* protein sequences available in Vector Base with the proteins described for other Dipterans from suborder Brachycera (genus *Drosophila*), suborder Nematocera (genus *Aedes*, *Culex*, *Anopheles*), and other members of the family Psychodidae (genus *Phlebotomus*). We also evaluated the expression pattern of GH13 and GH31 candidate genes in different tissues, under different feeding conditions, and the effect of *L. mexicana* infection in their expression. We identified 21 genes that belong to the GH13, described as α-amylases, α-glucosidases, amino acid transport proteins (heavy chain), 1,4-α-glucan branching enzyme and glycogen debranching enzyme; and six genes in the GH31 family, described as glycosidases NET37 (Nuclear envelope transmembrane glycosidase 37), lysosomal α-glucosidase and neutral α-glucosidase (α subunit). These enzymes are involved in sugar metabolism, storage, and mobilization of glycogen, protein transport, N-glycosylation quality control, and myogenesis regulation.

## Materials and Methods

### Chemicals

The TRI Reagent^®^ (Cat: T9424) was purchased from Sigma-Aldrich Company (St. Louis, MO, United States). Qubit ssDNA Assay Kit (cat: Q10212) and SYBR^TM^ Green PCR Master Mix (cat: 4309155) were obtained from Thermo Fisher Scientific (Waltham, MA, United States). QuantiTect Reverse Transcription Kit (Cat: 205310) was obtained from Qiagen (Hilden, Germany). Sheep blood with Alsever’s anticoagulant (Cat: SB068) was purchased from TCS Biosciences (Buckingham, United Kingdom). Other reagents used in this work were analytical grade.

### Search for GH13 and GH31 Sequences in *L. longipalpis* Genome

Sequences from GH13 and GH31 families from five different insect species – *Aedes aegypti* (AAEL006719, AAEL000647, AAEL008502, AAEL010602, AAEL009838, AAEL017226, AAEL022548), *Culex quinquefasciatus* (CPIJ005064, CPIJ007333, CPIJ011854, CPIJ001201, CPIJ009306), *Anopheles gambiae* (AGAP012230, AGAP010428, AGAP001200, AGAP001534, AGAP000862), *Drosophila melanogaster* (FBpp0311600, FBpp0071525, FBpp0070061, FBpp0084221), and *Drosophila ananassae* (FBpp0115773, FBpp0115136, FBpp0345570) – were retrieved from Vector Base^1^, Fly base^2^, and NCBI^3^ (Cantarel et al., 2009; Gabriško, 2013). These sequences were employed to perform ClustalW alignment and HMMER search in the Vector Base database to find similar sequences in the *L. longipalpis* genome (conserved domains on *Lutzomyia longipalpis*, LlonJ1.4, last updated 27 June 2017). Sequences recovered from *L. longipalpis* were annotated by similarity with proteins and conserved domains using the BLASTp (ref-seq protein) and Swiss-Prot/UniProt databases. Manual annotation was performed with the Apollo annotation tool (Dunn et al., 2019) and ClustalW (Thompson et al., 1994). Sequences were considered complete when initial methionine, correct exon/intron junction, stop codon were identified, and exon structures were complete based on the alignment with orthologous genes.

After sequences identification, characteristics like putative signal peptide (SignalIp) (Petersen et al., 2011), transmembrane domain (THMM) (Krogh et al., 2001), GPI-anchor (Big-PI predictor) (Eisenhaber et al., 1998), protein subcellular localization (DeepLoc) (Almagro Armenteros et al., 2017), N-glycosylation (NetNGlyc) (Gupta et al., 2002), O-glycosylation (NetoGlyc) (Steentoft et al., 2013), molecular mass and isoelectric point (pI) (Compute pI/Mw) (Bjellqvist et al., 1994; Gasteiger et al., 2005) were predicted *in silico*. The N-glycosylation sites were not considered if predicted inside transmembrane or cytoplasmic domains of membrane proteins. Genes and proteins representative structures were drawn using PROSITE MyDomains tool^4^ (Hulo et al., 2008).

The InterPro tool (Finn et al., 2017) was used to predict catalytic sites and, for α-amylases, also the calcium-binding site. The catalytic sites predicted by InterPro were confirmed by local alignment using ClustalW tool by comparing the *L. longipalpis* sequences with sequences of crystallized proteins deposited in the UniProt databank, and the analysis was based on conserved regions (CSRs) (Janecek, 1997; Kashiwabara et al., 2000; Lovering et al., 2005). This comparison was also used to predict the chloride binding sites in α-amylases. For GH13, the templates were the sequences of α-amylase from *Aspergillus oryzae* (Uniprot P0C1B3) (Toda et al., 1982), maltase from *Apis cerana japonica* (Uniprot A1IHL0) (Wongchawalit et al., 2006), amino acid transport protein (UniProt Q07837) (Bertran et al., 1993) from *Homo sapiens*, glucan branching enzyme from *E. coli* (UniProt P07762) (Baecker et al., 1986), and glycogen debranching enzyme from *Candida glabrata* (UniProt Q6FSK0) (Zhai et al., 2016). For GH31, the templates were the sequences of neutral α-glucosidase from *Homo sapiens* (UniProt O43451), lysosomal α-glucosidase from *Homo sapiens* (UniProt P10253) (Roig-Zamboni et al., 2017), and glycosidase NET37 from *Homo sapiens* (UniProt Q6NSJ0).

### Phylogenetic Tree Construction of GHs Proteins

Using each of the GH13 and GH31 sequences retrieved from *L. longipalpis* genome as a query, we searched for orthologous proteins in six other insect species. Sequences from *Aedes aegypti* (LVP_AGWG strain, AaegL5.1 geneset), *Anopheles gambiae* (PEST strain, AgamP4.9 geneset), *Culex quinquefasciatus* (Johannesburg strain, CpipJ2.4 geneset) and *Phlebotomus papatasi* (Israel strain, PpapI1.4 geneset) were retrieved from Vector Base^5^. Sequences from *Drosophila melanogaster* and *Drosophila ananassae* were retrieved from Fly base^6^. For *Phlebotomus papatasi*, sequences were searched and annotated as described above at “Search for GH13 and GH31 sequences in *L. longipalpis* genome.” The orthologous sequences used in this analysis are described in Supplementary Tables 1,2.

For phylogenetic analysis, sequences were aligned using Clustal Omega algorithm (version 1.2.2) (Sievers et al., 2011; Sievers and Higgins, 2018), after removing the signal peptide. The maximum likelihood phylogenetic tree was obtained using MEGAX software (Kumar et al., 2018) using partial deletion (90% site coverage cutoff). The bootstrap consensus tree was inferred from 500 replicates with cutoff of 50% for bootstrap values for the nodes.

### Maintenance of Insects

The experiments were performed using *L. longipalpis* (from Jacobina, Bahia, Brazil), maintained at Lancaster University (United Kingdom). Insects were kept under standard laboratory conditions of temperature (24 ± 2°C), and a photoperiod of 8 h of light/16 h of darkness. Adult sandflies were fed with 70% (w/v) autoclaved sucrose solution in cotton wool *ad libitum*, unless stated differently in the experiments. For oviposition, females were fed with sheep blood containing Alsever’s anticoagulant. For this, an artificial apparatus (Hemotek – Discovery Workshops, United Kingdom) at 37°C was used for 1 h using chicken skin as a membrane. Engorged females were transferred to rearing containers with a piece of cotton wool soaked in sugar solution. The eggs were separated from dead females after oviposition and reared to preserve the colony. For experiments, recently emerged females (0–3 h) were fed with 1.2 M sucrose for 2 days (SF females), with a piece of cotton wool soaked in the sugar solution, or were maintained for 3 days with a cotton wool soaked in water, before feeding on sheep blood. After blood-feeding (infected or not), they were kept with a piece of cotton wool soaked in water for 2 days.

### *Leishmania mexicana* Cultivation and Sandflies Infection

For infections, axenic cultures of amastigotes from *L. mexicana* strain M379 were used. For maintaining parasites in the amastigote form, they were incubated at 32°C with Grace’s insect medium supplemented with 20% of fetal bovine serum, BME_1_ vitamins, 2% human urine, and 25 μg/mL gentamycin sulfate. Parasites with a maximum of 26 passages in the amastigote maintaining medium, after isolation from the vertebrate host, were used for sandfly infections (Moraes et al., 2018). Infections were performed using a parasite concentration of 2 × 10^6^ parasites/mL, estimated using Neubauer chamber. Briefly, after centrifugation for 5 min at 2000 × *g*, the supernatant was removed, and parasites were mixed on sheep blood and offered to 4-day old unfed females as described in section “Maintenance of Insects.” Unfed females were discarded, and control insects were fed with uninfected blood. After blood-feeding, infected and control females were maintained as described above with water for 2 days.

### RNA Extraction and cDNA Preparation

Newly hatched females (0–3 h) (NF), females fed with 1.2 M sucrose for 2 days (SF females), control females fed with uninfected blood (BF females) and infected females (BF_INF females), 2 days after blood feeding, were dissected in sterile phosphate-buffered saline. Pools with 10 guts or rest of the body (RB) were transferred to polypropylene vials containing 50 μL of TRI Reagent^®^ (Cat. No. T9424, Sigma-Aldrich, Darmstadt, Germany) and kept at -80°C until further RNA extraction. For infected females, midgut was checked with light microscopy for parasite presence and only midguts containing parasites were used.

Total RNA was extracted following the supplier’s protocol and quantified using NanoDrop^®^ (NanoDrop Technologies, Wilmington, United States). The cDNA was synthesized by the Reverse Transcriptase (RT) reaction using the QuantiTect Reverse Transcription Kit (Qiagen, Hilden, Germany) according to the manufacturer’s protocol. cDNA was quantified using Nanodrop^®^ and normalized to a concentration of 50 ng/μL.

### Analysis of Gene Expression by RT-PCR or RT-qPCR

GH13 and GH31 coding sequences identified in the *L. longipalpis* genome were used to design specific oligonucleotides (Thornton and Basu, 2011), using the Primer3 program (Rozen and Skaletsky, 2000), Beacon Designer^TM^ Free Edition^7^, and mFOLD Software^8^. The list of primers is described in Supplementary Table 3. Gene expression was initially analyzed by RT-PCR, and at least one gene representative of each cluster (A, B, or C) of the GH13 α-amylase group (see section Results, Figure 3) was analyzed. Genes LLOJ000566/LLOJ008156 (LlAglu1/LlAglu2), LLOJ002257 (LlAglu3), LLOJ004841 (LlAamyA4) and LLOJ003489 (LlNAglu2), were additionally analyzed by RT-qPCR. Samples obtained from the gut and the rest of the body of recently emerged, sugar, and blood-fed females were used for RT-PCR or RT-qPCR analysis. Samples from females infected with *Leishmania mexicana* parasites were used only for the RT-qPCR analysis.

For RT-PCR, we carried duplicate reactions for each cDNA sample obtained from a tissue pool of 10 sand flies. Three biological replicates were carried for each group, with two cDNA samples obtained for each condition tested. Each amplification reaction was performed in a volume of 25 μL containing 2× Biomix Red (Bioline, London, United Kingdom), 100 ηg cDNA, and 0.2 μM of each primer. The PCR parameters were: incubation at 95°C for 5 min, followed by 26 cycles of 94°C for 30 s, 55°C for 40 s, 72°C for 1 min, and a final incubation of 72°C for 5 min. Relative expression was normalized using a housekeeping gene (LLOJ001891, Glyceraldehyde 3-phosphate dehydrogenase, GAPDH) as control. RT-PCR products were analyzed by electrophoresis in a 1% (w/v) agarose gel containing gel red^®^ (Biotium, Fremont, United States). Expression level was determined by densitometry of bands using the software ImageJ (Schneider et al., 2012).

Reverse transcriptions followed by quantitative polymerase chain reactions (RT-qPCR) were conducted using a CFX96 Touch^TM^ Real-Time PCR Detection System (BioRad). We carried duplicate reactions for each cDNA sample obtained from a tissue pool of 10 sand flies. Three biological replicates were carried for each group, with two cDNA samples collected for each condition tested. The reaction was performed in 10 μL volume containing 2× of SYBR^TM^ Green Master Mix (Thermo Fisher Scientific), 20 ηg cDNA, and 0.5 μM of each primer. The parameters for PCR were: incubation at 95°C for 15 min, followed by 39 cycles of 94°C for 15 s, 56°C for 30 s, and 72°C for 30 sec. As a negative control, PCR reactions were carried out without cDNA template to assess primer dimer formation or contamination in the reactions. To ensure that only a single PCR product was amplified, an analysis of the melting curve was performed. The expression of target genes in each tissue was quantified by the comparative Ct (ΔCt) method (Schmittgen and Livak, 2008) normalized with the GAPDH housekeeping gene as an internal control.

### Statistical Analysis

For multiple comparisons, one-way ANOVA was used, followed by Tukey’s multiple comparison tests, and significance was considered when *p* < 0.05. Results are expressed as the means ± SEM. All statistical analysis were executed using the Software GraphPad Prism 6.0 (San Diego, CA, United States).

## Results

### Sequence and Domain Analysis of the GH13 and GH31 Members in the Genome of *Lutzomyia longipalpis*

Proteins belonging to GH13 and GH31 are evolutionary conserved, being found in many classes of organisms. A total of 15 sequences belonging to the GH13 and four sequences from GH31 were found in the *L. longipalpis* genome, based on HMMER search in the Vector Base platform. For this, we used GH13 and GH31 know sequences of 5 dipteran species (*Aedes aegypti*, *Culex quinquefasciatus, Anopheles gambiae, Drosophila melanogaster, Drosophila ananassae*) as queries (Supplementary Tables 1,2).

Sequences of proteins found were classified based on similarity to other protein sequences and conserved domains using the BLASTp (ref-seq protein) and Swiss-Prot/UniProt databases according to their best hit. Results indicated that only 3 sequences from GH13 (LLOJ004841, LLOJ008156, LLOJ008629) and 1 from GH31 (LLOJ003489) were complete, considering the presence of initial methionine, stop codon, correct exon/intron junctions, and similar size of the exons when compared to orthologous proteins. Truncated sequences were analyzed and annotated for missing parts using the Apollo annotation tool and alignment by ClustalW. The transcriptomic data present in the Vector Base was also used to predict the correct size of exons. Modifications for each of the sequences retrieved are described in Supplementary Table 4. Some sequences could not be completed because they were in a region of poor sequencing quality or at the beginning or end of the scaffold. The nucleotide and protein sequences after annotation are presented in Supplementary Files 1 and 2, respectively.

**FIGURE 1:**
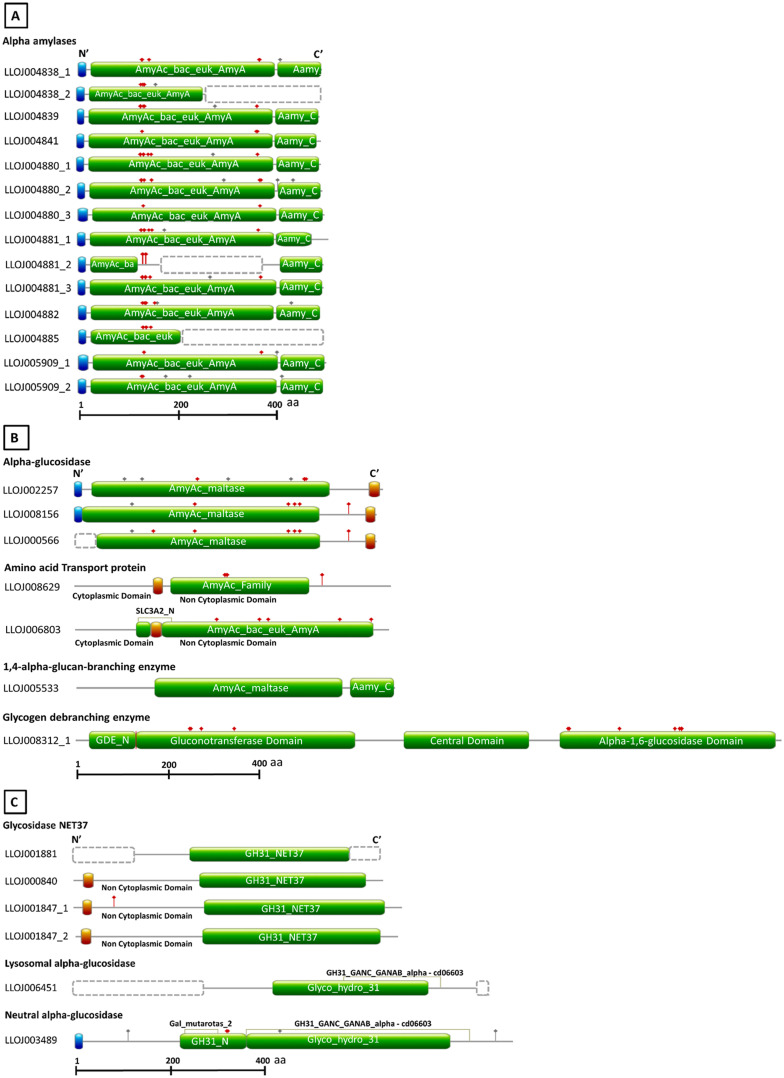
Schematic diagram of the domain architecture of *L. longipalpis* proteins from GH13 and GH31. Sequences were retrieved from Vector Base (Jacobina strain, LlonJ1.4 geneset, June 2017). Signal peptide, transmembrane domains, and domains used to identify the different catalytic activities are boxed with the blue, red and green background, respectively. Gray and red dots above structures represent the N- and O- glycosylation sites. Dotted boxes represent missing parts in incomplete protein sequences. For incomplete protein structures, models were designed based on homology with orthologous protein sequences. The structures were outlined using as reference the best hits obtained with Blastp (refseq_protein) and Conserved Domain Database. **(A)** Domain composition of GH13 α-amylases. **(B)** Domain composition of GH13 α-glucosidases, amino acid transport proteins, 1,4-α-glucan-branching-enzyme, and glycogen debranching enzyme. **(C)** Domain composition of GH31 glycosidases NET37, neutral α-glucosidase, and lysosomal α-glucosidase.

Some nucleotide sequences retrieved were coding for more than one protein belonging to GH13 or GH31. These sequences were split up, so the number of genes and, consequently, proteins increased. From sequences initially retrieved from Vector Base, genes LLOJ004838, LLOJ004880, LLOJ004881, LLOJ005909 codified for four different alpha amylases. After annotation, it was possible to determine that these genes actually codify for ten different alpha-amylases. Gene LLOJ004838, for example, codified for two different alpha-amylases that seem to be transcribed at different moments (corroborated by transcriptomic data from Vector Base). Protein1 (LlAamyA1) was transcribed in both larvae and adults and Protein 2 (LlAamyA2) was transcribed only in immature forms. Annotation resulted in 21 complete sequences for GH13 and 6 for GH31. With these analyses, we identified 14 α-amylases, tree α-glucosidases, two amino acid transport proteins (heavy chain), one 1,4-α-glucan branching enzyme, and one glycogen debranching enzyme from GH13 (Supplementary Table 5). For GH31, we identified four glycosidases NET37 (nuclear envelope transmembrane glycosidase 37), one lysosomal α-glucosidase, and one neutral α-glucosidase (α-subunit) (Supplementary Table 6). Gene structures are demonstrated in Supplementary Figure 1, and protein structures are represented in Figure 1.

According to the genomic map available in the Vector Base, the α-amylases genes were organized in two clusters. LlAamyA1 to LlAamyA4 (4 α-amylases) composed the cluster A, and the cluster B was formed by LlAamyB1 to LlAamyB8 (8 α-amylases). Besides that, the α-amylases C1 and C2 were in tandem in the genome. All the putative α-amylases were putative soluble extracellular proteins, with a predicted molecular mass from 54.6 to 56 kDa, and estimated isoelectric points from 4.4 to 6.4 (only complete sequences were analyzed, see Supplementary Table 5). α-glucosidases were predicted as membrane proteins. LlAglu1 and LlAglu2 were putative transmembrane proteins, and for LlAglu3, both transmembrane domain and GPI anchor were predicted. These enzymes had molecular masses around 70 kDa and estimated isoelectric points from 4.7 to 5.6 (Supplementary Table 5). A lysosomal membrane α-glucosidase (LlLysAglu1, LLOJ000840) with 76.3 kDa and a soluble α-glucosidase (LlNAglu1, LLOJ003489), putatively active in the endoplasmic reticulum, were identified in the GH31 family (Supplementary Table 6).

Analysis with InterPro and amino acid sequences alignments of *L. longipalpis* with GH13 and GH31 representative sequences from different organisms allowed the identification of highly conserved regions, catalytic residues, and the calcium and chloride binding sites for α-amylases (Tables 1, 2). The active site containing the catalytic residues, the calcium-binding site, and the chloride-binding site (Supplementary Table 5 and Table 1) were correctly identified for α-amylases sequences belonging to cluster A and also for LlAamyB7 and LlAamyC2. The α-amylase B1 (LlAamyB1/LLOJ004880_1) had not a typical catalytic triad and presented only two residues that might serve as the nucleophile and the acid/base catalyst (D204 and D241, respectively). Moreover, this enzyme also showed two substitutions: i) one in the calcium-binding site, a conserved histidine residue by asparagine, and ii) one in the chloride-binding site at the β4 sheet, a conserved arginine by leucine (Supplementary Table 5 and Table 1). Some of the α-amylases might not function as active enzymes, since they lacked the correct nucleophile and the acid/base catalyst, as α-amylases B4 and B6 (LlAamyB4/LLOJ004881_1 and LlAamyB6/LLOJ004881_3).

**TABLE 1:** Conserved amino acid sequences in proteins belonging to GH13.

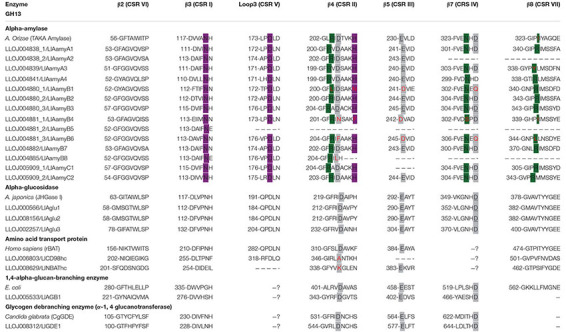

*Identification based on conserved regions described in Janecek (1997). The catalytic conserved residues, calcium binding and chloride binding residues are with gray, purple and green background, respectively. Non-canonical residues are marked in red.*

**TABLE 2:** Conserved amino acid sequences in proteins belonging to GH31.

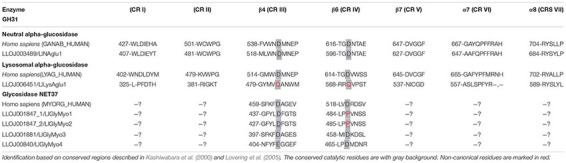

*Identification based on conserved regions described in Kashiwabara et al. (2000) and Lovering et al. (2005). The conserved catalytic residues are with gray background. Non-canonical residues are marked in red.*

The catalytic triad was D216, E290, D357 for both α-glucosidases 1 and 2 (LlAglu1, LLOJ000566 and LlAglu2, LLOJ0008156), and D236, E304, D375 for α-glucosidase 3 (LlAglu3, LLOJ0002257) with all highly conserved regions preserved (Supplementary Table 5 and Table 1).

The amino acid transport proteins (heavy chains) found in our screening shared a domain similarity with alpha-glucosidases, but the general protein primary structure was different (Figure 1), and they lacked the conserved catalytic residues (Table 1). By similarity, LLOJ008629 was described as “neutral and basic amino acid transport protein” (NBAThc), while LLOJ006803, due to the presence of the SLC3A2 domain, was classified as CD98hc (cluster of differentiation 98) (Supplementary Table 5 and Table 1).

The sequence of a 1,4-α-glucan branching enzyme (LlAGB1, LLOJ005533) had a central domain containing the active site, and the catalytic residues similar to the other enzymes from GH13. In the putative sequence of a glycogen debranching enzyme (LlGDE1, LLOJ008312), we identified both α-1,4 glucanotransferase (GT) and α-1,6 glucosidase (GC) domains, this enzyme was a soluble cytoplasm enzyme with an estimated size of 172.6 kDa. The GT domain at the N-terminal region of this protein, despite the low overall sequence identity, had similarity with five short conserved regions of the GH13; the catalytic triad was conserved with the D548 as the catalytic nucleophile, E577 as the proton donor, and the conserved residue D649. The C-terminal GC domain was similar to the catalytic domain of glucoamylases and other members from GH15 family. The general acid and general base for GC activity were identified as D1282 and E1515, respectively (Supplementary Table 5 and Table 1).

Regarding the members of GH31 family, the neutral α-glucosidase (α-subunit) (LlNAglu1, LLOJ003489) was described with both Galactose mutarotase domain at the N-terminal end and the GANAB_GANAC domain (Figure 1C). This enzyme shared a 46.8% identity with the human neutral α-glucosidase (GANB_HUMAN) and was predicted to be localized at the endoplasmic reticulum. The two aspartic residues involved in the catalytic domain were identified as D522 and D598 (Supplementary Table 6 and Table 2). The lysosomal α-glucosidase (LlLysAglu1, LLOJ006451) presented a substitution of both catalytic aspartate residues by glutamine (Supplementary Table 6 and Table 2). The glycosidases NET37 were predicted to have a single transmembrane domain at N-terminus and the GH31_NET37 domain at its C-terminus. The glucosidase domain was facing the non-cytoplasmic region (Figure 1C). Since these proteins are dependent on the catalytic residues to be active, glycosidase NET37 1 (LlGlyMyo1, LLOJ001847_1) and NET37 2 (LlGlyMyo2, LLOJ001847_1), seemed to lack hydrolytic activity. The proton donor residue was substituted for proline (Supplementary Table 6 and Table 2). Although the sequence for glycosidase NET37 3 (LlGlyMyo3, LLOJ001881) was not complete, it was possible to predict the two catalytic residues, 401D and 460D. Glycosidase NET37 4 (LlGlyMyo4, LLOJ000840) had glutamate as the conserved catalytic nucleophile, E408, and D467 as a proton donor.

### Gene Numbers and Phylogenetic Analysis of GH13 and GH31 Sequences

**FIGURE 2:**
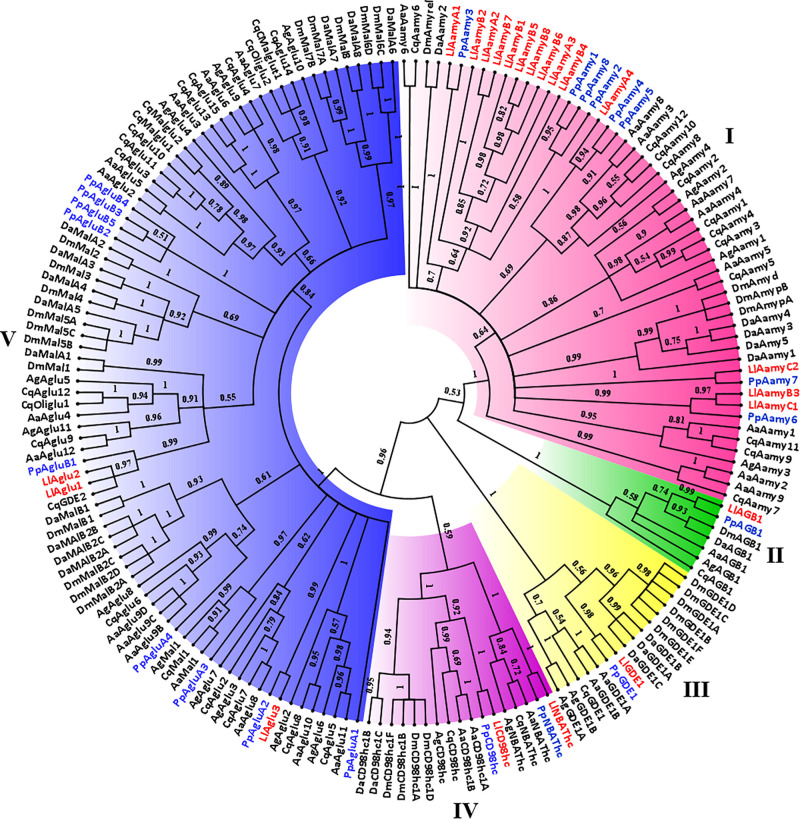
Phylogenetic analysis of *L. longipalpis* GH13 proteins. Radial cladogram represents the alignment of GH13 protein sequences, α-amylases (Aamy, red), α-glucosidases (Aglu or Mal, blue), amino acid transport proteins (CD98hc and NBAThc, purple), 1,4-α-branching enzymes (AGB, green), glycogen debranching enzymes (GDE, yellow), of *L. longipalpis* and other dipterans species. *L. longipalpis* sequences are highlighted in red and *P. papatasi* in blue. *L. longipalpis* (Ll), *P. papatasi* (Pp), *A. aegypti* (Aa), *A. gambiae* (Ag), *C. quinquefasciatus* (Cq), *D. melanogaster* (Dm) and *D. ananassae* (Da). The tree represents the bootstrap consensus of 500 replicates, visualized with FigTree v1.4.3. Bootstrap values for the nodes are represented. The identifiers and corresponding genes are specified in Additional file 1.

A phylogenetic tree was constructed, aiming to support the classification and prediction of the functional role of the proteins. Using the *L. longipalpis* GH13 and GH31 protein sequences, we identified orthologs in other dipteran species and verified if gene amplification might have occurred in these GH families in the *Phlebotominae* branch. Phylogenetic analysis of GH13 protein sequences demonstrated the occurrence of 5 clades, each of them representing one of the activities described in this family (Figure 2). Within each clade, Phlebotominae proteins are more closely related to each other than to their orthologs in other dipteran species (Figure 2).

Clade I represented the α-amylases, and one of the subgroups of this clade had representatives only from the Phlebotominae subfamily. This subgroup was composed by 10 of 14 (71%) α-amylases of the *L. longipalpis* genome, suggesting a gene expansion in this protein family that included three sequences from cluster A (LlAamyA1, LlAamyA2, LlAamyA3) and seven sequences from cluster B (LlAamyB1, LlAamyB2, LlAamyB4, LlAamyB5, LlAamyB6, LlAamyB7, LlAamyB8) (Figure 2). The glucan branching enzymes were represented by a monophyletic group in clade II, demonstrating the sequence conservation among dipteran species. Enzymes from *L. longipalpis* and *P. papatasi* are grouped with the *D. melanogaster* and *D. ananassae* (Figure 2). Clade III represented the debranching enzyme, and was formed by a paraphyletic group, since the Phlebotominae subfamily, Drosophilidae, and Culicidae families established different branches (Figure 2). Clade IV was composed by the amino acid transport proteins, and formed a paraphyletic group divided into two subgroups. One subgroup represents the neutral and basic amino acid transporters (NBATs), and the other subgroup, the CD98 heavy chain, comprised the large neutral amino acid transport (LAT1). NBATs proteins missed the SLC3A2 domain, which is characteristic of CD98hc. The LlCD98hc grouped with orthologs from the Drosophilidae family, already classified as different isoforms of the CD98 heavy chain (Figure 2).

Clade V represented the α-glucosidase proteins, and *L. longipalpis* α-glucosidases were grouped with *P. papatasi* orthologs. Two subgroups had representatives only from the Drosophilidae family (Figure 2). As described in Table 3, *P. papatasi* seemed to have an expansion in the α-glucosidase genes when compared to *L. longipalpis*. One of the subgroups of this clade had representatives only from *P. papatasi.* Interestingly, the PpAgluA3 is ortholog to the CqMal1 (Figure 2), an enzyme from *C. quinquefasciatus* characterized as GPI membrane-bound α-glucosidase with toxin binding properties (do Nascimento et al., 2017).

**TABLE 3:** Comparison of GH13 and GH31 gene numbers retrieved from Vector Base, Fly Base and NCBI in different dipteran species.

	***L. longipalpis***	***P. papatasi***	***A. aegypti***	***A. gambiae***	***C. quinquefasciatus***	***D. melanogaster***	***D. ananassae***
**GH13**							
Alpha-Amylase	14	8	9	4	12	3 (4)	5
Alpha-glucosidase	3	9	12 (14)	11	19	10 (16)	10 (12)
1,4-alpha-glucan-branching enzyme	1	1	1	1	1	1	1
Amino acid transport protein heavy chain	2	2	2 (3)	2	2	1 (4)	1 (2)
Glycogen debranching enzyme	1	1	1 (2)	1 (2)	2	1 (6)	1 (3)
**GH31**							
Lysosomal alpha-glucosidase	1	1	1 (8)	1	1	0	0
Neutral alpha-glucosidase α subunit	1	1	2	1	2	1 (5)	1
Glycosidase NET37	4	2	6 (10)	3 (5)	3	2 (5)	2 (4)

*The number between brackets represents the total number of transcripts.*

**FIGURE 3:**
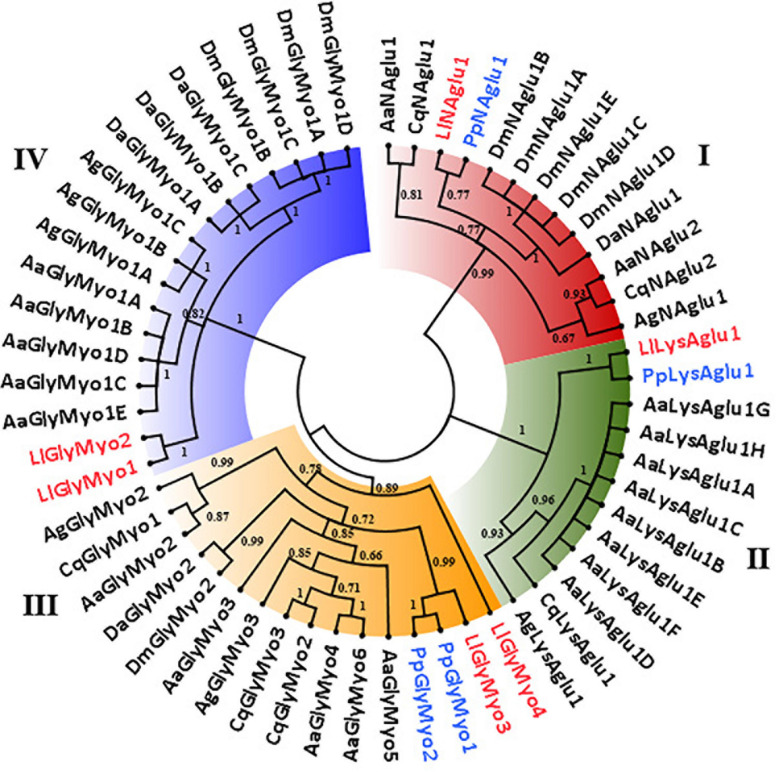
Phylogenetic analysis of *L. longipalpis* GH31 proteins. Radial cladogram represents the alignment of GH31 protein sequences, neutral α-glucosidases (NAglu, red), lysosomal α-glucosidases (LysAglu, green), Glycosidases NET37 with catalytic domain (GlyMyo, orange), and Glycosidases NET37 with no catalytic domain (GlyMyo, blue) of *L. longipalpis* and other dipterans species. *L. longipalpis* sequences are highlighted in red and *P. papatasi* in blue. Sequences are from *L. longipalpis* (Ll), *P. papatasi* (Pp), *A. aegypti* (Aa), *A. gambiae* (Ag), *C. quinquefasciatus* (Cq), *D. melanogaster* (Dm) and *D. ananassae* (Da). The tree represents the bootstrap consensus of 500 replicates, visualized with FigTree v1.4.3. Bootstrap values for the nodes are represented. The identifiers and corresponding genes are specified in Additional file 2.

Phylogenetic analysis of GH31 protein sequences demonstrated the occurrence of 4 clades representing each activity described in this family (Figure 3). Once more, within each clade, *Phlebotominae* proteins related more closely to each other when compared to their orthologs in other dipteran species, except for clade IV, because it had no representative from *P. papatasi* (Figure 3). Clade I and II represented the lysosomal and the neutral alpha-glucosidases, respectively. As described in Table 2, neutral alpha-glucosidases maintained their catalytic residues. The LlLysAglu1 sequence had no conserved catalytic residue, and the phylogeny analysis grouped it with lysosomal enzymes from other dipteran species that also had not catalytic residues (Table 2 and Figure 3). The other two clades, III and IV, contained the glycosidases NET37. For NET37 glycosidases, clade III represented proteins containing the conserved catalytic domain, while clade IV represented glycosidases without the catalytic residues (Table 2 and Figure 3).

When we compare the number of genes for GH13 and GH31 in the *L. longipalpis* genome with their orthologs in 6 dipteran species, a potential expansion of α-amylases genes was observed in *L. longipalpis* (Table 3). The total of 14 α-amylase sequences found was 75% and 16% higher when compared to *Phlebotomus papatasi* and *Culex quinquefasciatus*, respectively, and almost 3-fold the number of α-amylases found in the representatives of the *Drosophilidae* family. Interestingly, a contraction occurred for α-glucosidases genes in *L. longipalpis*, when compared to the other six species (Table 3). The number of genes coding for α-1,4-glucan branching and glycogen debranching enzymes (GDE) was conserved among the species analyzed (Table 3).

For GH31 family, the number of lysosomal and neutral α-glucosidase was conserved in phlebotomine and mosquitoes. *Drosophila* species had no lysosomal α-glucosidases. Glycosidase NET37 demonstrated a variable number of copies among the species analyzed and seemed to be duplicated in *L. longipalpis* when compared to *P. papatasi* (Table 3).

### Expression of *L. longipalpis* GH13 and GH31 Genes in Different Physiological Conditions

After analyzing the sequences using bioinformatics tools, primers were designed for selected sequences in the GH13 family and all sequences of GH31. In the case of α-amylases, we analyzed at least one representative for each phylogenetic cluster of Figure 2. Also, it was not possible to design specific primer pairs to independently analyze expression of genes coding for LlAglu1/LlAglu2 or LlMyoGly1/LlMyoGly2, due to the high sequence identity between these sequences.

Primers amplified all sequences producing only one amplicon with the expected size from genomic DNA (data not shown). The expression of the *L. longipalpis* GH transcripts was evaluated by RT-PCR using different tissues (rest of body or gut) as sources of RNA, and at different dietary conditions, namely newly hatched females, sugar or blood-fed females.

**FIGURE 4:**
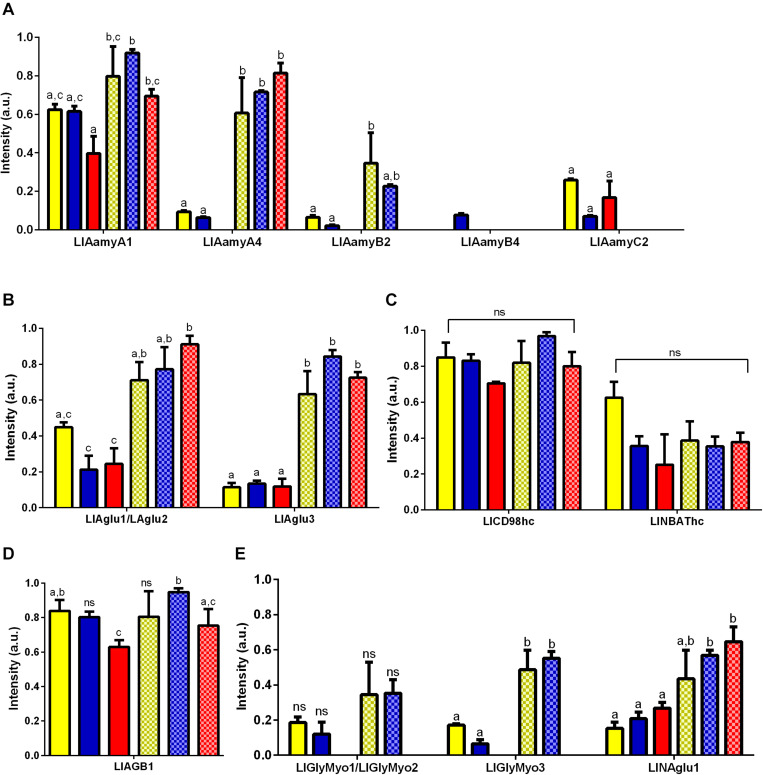
Expression pattern of genes belonging to GH13 and GH31 families in *L. longipalpis* by RT-PCR. Expression was analyzed in different tissues of females submitted to different physiological conditions. The expression of genes from GH13 was analyzed for **(A)** α-amylases (LlAamyA1, LlAamyA4, LlAamyB2, LlAamyB4 and LlAamyC2); **(B)** α-glucosidases (LlAglu1/LlAglu2 and LlAglu3); **(C)** amino acid transport proteins heavy chain (LlCD98hc and LlNBAThc); **(D)** 1,4-glucan-branching enzyme (LlAGB1). For the GH31, the expression pattern was analyzed for **(E)** glycosidases NET37 (LlGlyMyo1/LlGlyMyo2 and LlGlyMyo3) and neutral alpha-glucosidase (LlNAglu1). Samples of the rest of the body (plain bars) and gut (dotted bars) were analyzed for each physiological condition tested; newly hatched females (yellow bars), females fed on 1.2 M sucrose for 48 h (blue bars) and females after 48 h of blood-feeding (red bars). Results were the mean ± SEM of three biological replicates. Each replicate included two cDNA samples for each condition tested. cDNA samples were obtained from a pool of 10 sand flies (gut or rest of the body). One-way ANOVA, followed by Tukey multiple comparison tests. In the subset of results for each gene, bars with the same letters are not statistically different to each other, *p* > 0.05. ns: results with no significant differences to the other bars of the subset.

All tested tissues and conditions poorly expressed both LlGlyMyo4 and the lysosomal α-glucosidase (data not show). The LlAamyA1 and LlAamyA4 were the α-amylases with the highest levels of expression in the gut when compared to other α-amylases genes independently of dietary condition tested (Figure 4A). No significant difference was found for LlAamyA1 expression levels when comparing different dietary conditions inside the same tissue. However, when comparing different tissues, LlAmy1 was significantly more expressed in the gut of sugar-fed females (one way ANOVA, ^∗∗^*p* < 0.01) and blood-fed females (One way ANOVA, ^∗^*p* < 0.05) than in rest of the body (Figure 4A). LlAamyA4 expression was higher in the gut for all dietary conditions tested (One way ANOVA, ^****^p < 0.0001) and unaltered considering the dietary condition inside the same tissue (Figure 4A). Interestingly, blood-fed females did not express LlAamyB2. LlAamyB4 was poorly expressed in all tested conditions, while the expression of LlAamyC2 only occurred in the rest of the body (Figure 4A). α-glucosidase transcripts (LlAglu1/LlAglu2 and LlAglu3; Figure 4B) presented an expression pattern similar to LlAamyA1 and LlAamyA4, and no significant differences were found in expression levels when comparing different dietary conditions inside the same tissue. For LlAglu1/LlAglu2, the level of expression in the gut was higher for sugar-fed (One way ANOVA, ^∗∗^*p* < 0.01) and blood-fed females (One way ANOVA, ^****^p < 0.0001), when compared to the rest of the body (Figure 4B). LlAglu3 expression was higher in the gut for all dietary conditions tested (One way ANOVA, NF ^∗∗^*p* < 0.01 and SF/BF ^∗∗∗^*p* < 0.001) (Figure 4B).

The expression of the α-1,4-glucan branching enzyme and the amino acid transporters is not linked to a specific tissue, and LlNBAThc and LlCD98hc presented no significant changes in expression (Figure 4C) in different feeding conditions. The expression of LlAGB1 (Figure 4D) was reduced when females feed on blood.

For the GH31, blood-fed females did not express glycosidases NET37 in any tissue. LlGlyMyo1/LlGlyMyo2 were expressed in the gut and carcass of newly emerged and sugar fed insects, but with no significant differences among these samples (Figure 4E). LlGlyMyo3 was also expressed in these same tissues and conditions, but it was more expressed in the gut when compared to the rest of body (*p* < 0.05, Figure 4E). There were no significant differences in the expression of LlGlyMyo3 in the gut and rest of body when comparing newly emerged flies with sugar-fed ones (Figure 4E). LlNAglu1 was significantly more expressed in the gut when compared to the rest of body, and its expression in each tissue was not affected by the feeding conditions (Figure 4E).

### Gene Expression of LlAamyA4, LlAglu1/LlAglu2, LlAglu3, and LlNAglu2 in *L. longipalpis* Infected With *Leishmania mexicana*

Since we have an interest in sandfly proteins that might be affected by *Leishmania*, we selected the genes coding for LlAamyA4, LlAglu1/LlAglu2, LlAglu3 (GH13) and LlNAglu1 (GH31) for further quantitative expression analysis after *L. mexicana* infections. These genes were chosen considering their preferential expression in the gut, the organ which was in direct contact with the parasite during the experimental infections.

**FIGURE 5:**
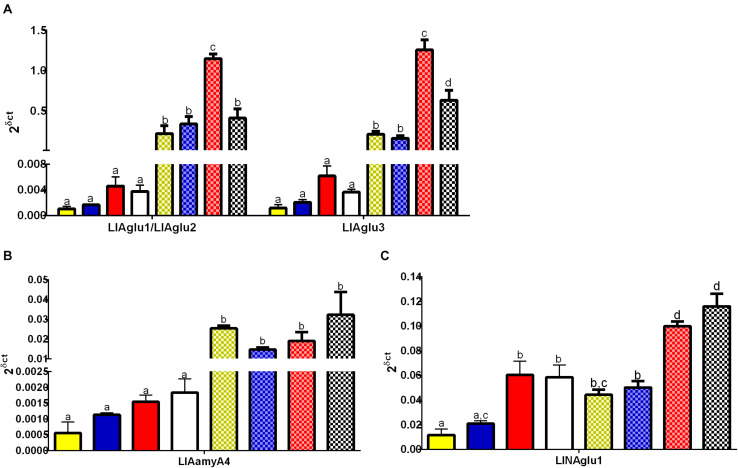
Expression pattern of genes belonging to the GH13 and GH31 in *L. longipalpis* by qRT-PCR (2^δ^ ^ct^). Expression was analyzed in different tissues for adult females submitted to different physiological conditions. The expression levels of genes belonging to GH13 were analyzed for **(A)** α-glucosidases (LlAglu1/LlAglu2 and LlAgGlu3), and **(B)** α-amylase (LlAamyA4). For the GH31, the expression patterns were analyzed for **(C)** neutral α-glucosidase (LlNAglu1). Samples of the rest of the body (plain bars) and gut (dotted bars) were analyzed, for each physiological condition tested: newly hatched females (yellow bars), females fed on 1.2 M sucrose for 48 h (blue bars), females at 48 h after blood-feeding (red bars), and females at 48 h after infection with *L. mexicana* (white bars). Results were the mean ± SEM of three biological replicates. Each replicate included two cDNA samples for each condition tested. cDNA samples were obtained from a pool of 10 sand flies (gut or rest of the body). One-way ANOVA, followed by Tukey multiple comparison tests. In the subset of results for each gene, bars with the same letters are not statistically different to each other, *p* > 0.05.

RT-qPCR analysis of the genes LlAglu1/LlAglu2, LlAglu3, LlAamyA4 (GH13), and LlNAglu1 (GH31) confirmed higher gene expressions in gut tissues when compared to the rest of the body, in all conditions tested (One way ANOVA, ^∗^*p* < 0.05) (Figure 5). LlAglu1/LlAglu2 and LlAglu3 genes showed higher quantitative expression values when compared to the genes LlAamyA4 and LlNAglu1. Expression values for LlAglu1/2 and LlAglu3 were about 40 X higher than LlAamyA4 and 10 X higher than those observed for LlNAglu1 (Figure 5).

Interestingly, the expression levels of the putative α-glucosidases LlAglu1/2 and LlAglu3 were significantly affected by the blood diet and *Leishmania* infection (Figure 5). We observed an induction in the expression levels of LlAglu1/LlAglu2 (1.14 ± 0.06) and LlAglu3 (1.2 ± 0.1) in the gut of blood-fed females in comparison to non-fed or sugar-fed females (One way ANOVA, ^****^p < 0.0001; Figure 5A). Also, infection with *L. mexicana* parasite modulated negatively both the expression of LlAglu1/LlAglu2 (0.4 ± 0.1) and LlAglu3 (0.6 ± 0.1) (Figure 5A) (One way ANOVA, ^****^p < 0.0001). The sugar diet did not significantly affect expression of these genes when compared to the non-fed condition.

LlAamyA4 had the lowest level of expression among the genes studied and presented the same pattern obtained previously using semi-quantitative RT-PCR, with no significant changes after sugar or blood feeding, when comparing to newly emerged flies (Figure 5B). Besides that, LlAamyA4 expression was not affected by parasites in the blood diet (Figure 5B).

LlNAglu1 is not highly expressed in the gut tissue compared to the rest of the body, but there is a significant difference between these tissues for all dietary conditions tested (Figure 5C). The expression level of LlNAglu1 was also induced by the blood diet in both tissues in comparison to non-fed condition or sugar diet (One way ANOVA, ^****^p < 0.0001; Figure 5C), but in this case the expression was not modulated by infection with *L. mexicana* parasites (Figure 5C).

## Discussion

Glycosidases play a significant role in the digestion of plant and blood sugars in sand flies, especially α-amylases and α-glucosidases. In this work, the majority of the 21 genes belonging to the GH13 family, in the genome of *L. longipalpis*, were classified as enzymes typically involved in carbohydrate digestion, especially 14 α-amylases, and three alpha-glucosidases. Proteins involved in glycogen metabolism (1,4-α-glucan branching enzyme and glycogen debranching enzyme, lysosomal α-glucosidase), amino acid transport (NBAThc and CD98hc), quality control of N-glycosylation in the endoplasmic reticulum (neutral α-glucosidase α subunit) and myogenesis regulation (NET37), were also described in GH13 or GH31 families.

We described potentially extracellular soluble α-amylases with predicted molecular masses around 55 kDa, and isoelectric points (pIs) between 4.5 and 6.5. We also described putative membrane-anchored alpha-glucosidases with molecular masses around 70 kDa and pIs between 4.5 and 5.5. In general, insect α-amylases have molecular weights about 48–60 kDa, pI 3.5–4.0, are calcium-dependent enzymes, and in some cases, they are also activated by chloride (Terra and Ferreira, 2012). The insect α-glucosidases have molecular weights ranging from 60 to 80 kDa (or multiple of these), with pI of 5.0–7.2 (Terra and Ferreira, 2012).

For insects in general, α-amylases are found as soluble enzymes in the midgut lumen, and α-glucosidases can also be seen as soluble enzymes, or anchored to the microvillar or perimicrovillar membranes of midgut cells (Silva and Terra, 1995; Nishimoto et al., 2001; Cristofoletti et al., 2003). The LlAglu1, LlAglu2, and LlAglu3 described for *L. longipalpis* are probably membrane-anchored proteins, and their corresponding genes showed higher expression in the gut, when compared to the rest of the body in adult sandflies (Figures 4B, 5A). Their expression was higher when females fed with blood but did not change in sugar-fed females compared to non-fed newly emerged flies (Figure 5A). Besides that, infection with the parasite *L. mexicana* modulates their expression. α-glucosidases were described as membrane proteins anchored to the midgut glycocalyx in *L. longipalpis*, with a basal activity in non-fed females and activity induction starting 24 h after sugar feeding (Gontijo et al., 1998; da Costa et al., 2019).

Our expression analysis suggests that the increase of α-glucosidase activity previously reported in sugar-fed females (da Costa et al., 2019) was not due to transcriptional gene regulation. No increase in expression was observed for any gene encoding α-glucosidase when females were sugar-fed compared to newly emerged flies, which contrasts with the observation that the gut sucrase activity increases 3× after sugar feeding for 48 h (da Costa et al., 2019). Besides that, sucrase induction is dose-dependent and responds preferentially to sucrose (da Costa et al., 2019). Another possibility is that transcription is regulated transiently, and after 48 h, it was not possible to determine any gene induction or repression. In adults and larvae of *D. melanogaster*, it has been described that some genes encoding carbohydrate digestive enzymes, as MAL-A1, are repressed in response to dietary sugars, due to a mechanism of regulation by negative feedback. The induction of MAL-A4 gene transiently occurs when maltose is offered to larvae and repressed when fed with glucose (Chng et al., 2017; Yamada et al., 2018).

A soluble form of α-glucosidase was described for *L. longipalpis* and *P. langeroni* when females were blood-fed (Dillon and El Kordy, 1997; da Costa et al., 2019). Biochemical assays using different tissues as enzyme source, in different conditions, and chromatographic analysis demonstrated the presence of at least four different α-glucosidases in *L. longipalpis* (da Costa et al., 2019). In this work, we identified three genes coding for membrane-anchored α-glucosidases, that are positively regulated 48 h after blood feeding. These genes may undergo alternative splicing, producing different isoforms of α-glucosidases. Removal of the last exon might eliminate the transmembrane region and allow these enzymes to be secreted in soluble form, changing the biochemical characteristics of the enzymes. Additionally, the protein LlAglu3 is putatively anchored to the membrane by a GPI anchor, and this might undergo cleavage by phospholipases releasing the protein from the midgut membrane. The soluble form of these α-glucosidases may pass through the peritrophic membrane and act synergistically with trypsin and other enzymes that release glycopeptides or glycolipids from blood glycoconjugates in the endoperitrophic space. Also, the membrane-bound or soluble α-glucosidases might play a role in the process of heme detoxification during blood digestion, during the hemozoin formation. This physiological function was described for the α-glucosidases of *R. prolixus* (Mury et al., 2009). In this respect, *L. longipalpis*α-glucosidases may play different roles in the hydrolysis of glycosides, during the digestion process, which may be associated with different enzyme specificities for sugars or glyco-derivatives, as glycolipids or glycoproteins.

In insects challenged with *Leishmania*, transcriptomic studies have demonstrated changes in the expression of several immune-related genes, oxidative stress-related molecules, and some digestive enzymes, mainly proteases (Dillon et al., 2006; Ramalho-Ortigão et al., 2007; Jochim et al., 2008; Abrudan et al., 2014). These works indicate that inside the vector, a systemic response is activated to defeat the *Leishmania* infection. However, a recent report showed that, after infection of *L. longipalpis* with *L. infantum*, only a small subset of genes (less than 1% of the transcriptome) showed substantial changes in expression (Coutinho-Abreu et al., 2020). Interestingly, at the same time window used in our experiments (48 h after infection), these authors observed downregulation of upstream factors related to nutrient transport, digestive enzymes, and peritrophic matrix proteins, which is coherent with our findings. In this context, *Leishmania* may likely modulate the physiological responses of phlebotomines, including changes in their microbiota, to overcome several biochemical and molecular challenges for the establishment of infection. The result may be the reduction of an enzymatic activity that may directly hydrolyze parasite surface molecules, as lipophosphoglycans (Ilg et al., 1992) or glycoinositolphospholipids (GIPLs; McConville et al., 1993), or may compete with *Leishmania* glycosidases or receptors for the sucrose present in the anterior parts of the insect’s gut (Lyda et al., 2015).

Modulation of different sandfly enzymes by *Leishmania* parasites has been described, emphasizing proteases (Schlein and Romano, 1986; Dillon and Lane, 1993; Telleria et al., 2010; Santos et al., 2014). However, to our knowledge, this was the first time that the modulation of sandfly carbohydrases, caused by *Leishmania* infection, was specifically demonstrated. The modulation of α-glucosidase genes in *L. longipalpis* during *L. mexicana* infection may be related to a systemic response of the organism to the infection, which may cause the activation of different components of the immune response pathways, consequently affecting the carbohydrate metabolism as a whole. Furthermore, GalNAc-containing glycoproteins present in the microvillar membranes of sandflies midgut are involved in the mechanism of attachment of *Leishmania* in permissive sandfly species (Myskova et al., 2007).

Alpha-glucosidases are enzymes abundantly found in the microvillar membrane of *L. longipalpis* midgut, and as described in this work, they have some potential N-glycosylation sites. In another hypothesis, these enzymes might be acting as a site for the attachment of *L. mexicana* in the midgut of *L. longipalpis.* As a response to the infection, the vector might downregulate the expression of the α-glucosidase genes. However, it remains elusive if these changes in the expression of *L. longipalpis* glucosidase genes are related to specific regulation mechanisms or just results of a general shift in the digestive process caused by the pathogen. Considering that we assessed just one-time point (48 h after feeding with an infected blood meal), changes in the whole temporal digestion pattern might explain our results. It is important to consider, in this context, that our previous reports in the same model showed an increase in trypsin activity but no change in the general course of protein degradation (Moraes et al., 2018). This may suggest differences in the regulation of protease and carbohydrase genes in the gut of *L. longipalpis*.

We described three α-glucosidase coding genes in *L. longipalpis*, suggesting a retraction compared to other dipterans, as *Drosophila* and even *P. papatasi*. This may be related to a strong specialization of these enzymes for the hydrolysis of sucrose, in an environment for *Lutzomyia* with higher offer of sucrose-rich nectar as the tropical forest, when compared to the savanna or desert associated to *P. papatasi*. Supporting this hypothesis, there is previous biochemical evidence that *L. longipalpis* α-glucosidases have strong specificity for sucrose as a substrate (da Costa et al., 2019).

Although the primary role of α-glucosidases is the metabolism of sugars, in the process of evolution, these proteins might have acquired additional functions. In some mosquitoes species, like *C. pipiens, C. quinquefasciatus*, and *A. gambiae*, the α-glucosidase is also described as the receptor of the Bin toxin from *Lysinibacillus sphaericus* (Darboux et al., 2001; Romão et al., 2006; Opota et al., 2008). The expression levels of maltase/α-glucosidase genes for *D. melanogaster* are different when considering tissues and phases of development, suggesting a non-enzymatic regulatory function for these proteins (Gabriško, 2013). There are some examples where enzymes have lost their catalytic activity, developing new functions, but still have high sequence similarity with α-glucosidases. This phenomenon occurs for amino acid transporters (neutral and basic amino acid transport protein (NBAT) and the cluster of differentiation 98 (CD98) (Figure 2). We described two coding genes for these amino acid transport proteins (heavy chain) in *L. longipalpis*. The amino acid transporter is a heterodimeric protein, and the heavy chain has the function of correctly locating and folding the light chain on the plasma membrane. The light chain subunit has the purpose of transporting amino acids across the cell membrane (Gabriško and Janeček, 2009).

Interestingly, the α-amylase genes described for *L. longipalpis* are organized in two clusters (A and B), and the α-amylases C1 and C2 are in tandem in the genome in a different region. LlAamyC2 corresponds to the protein AF132512, previously described as a salivary α-amylase (Charlab et al., 1999). These clustered genes have the same putative enzymatic specificity, are closely spatially localized, and have high sequence similarity to each other, so probably these sequences originated from multiple subsequent genomic duplications. *Phlebotomus papatasi* and *C. quinquefasciatus* also present the clustering of some α-amylases. Our results suggest a gene expansion of *L. longipalpis* α-amylase family compared to *P. papatasi*, mosquitoes, and the genus *Drosophila* (Figure 2 and Table 3).

As a natural defense system, plants produce many aproteic and proteinaceous inhibitors such as acarbose, cyclodextrins, acarviosine-glucose, lectin-like, and knottin type to prevent the digestion by herbivorous insect’s midgut alpha-amylases (Franco et al., 2002). Insects feeding on plant sap, as phlebotomines, might also be affected by these inhibitors. The expansion of the α-amylase gene family in *L. longipalpis* might reflect a diversification of these activities to overcome plant inhibitors or even a to account for tissue expression specificity. In the natural habitat of *L. longipalpis*, tropical forest, there is a greater diversity of available food sources when compared to the habitat of *P. papatasi*, savanna and desert (Akhoundi et al., 2016). It was demonstrated that even inside the *P. papatasi* species, there is a variation in the spectrum of glycosidase activities when comparing phlebotomines collected in an oasis (sugar-rich) to those obtained in the dry-season desert (Jacobson et al., 2007). However, this hypothesis involves an adaptation that depends on the insect development stage. In this respect, it would be interesting to investigate if these α-amylases genes are differentially expressed throughout the entire sand fly life cycle, since amylase activity has been described both in adults and larvae (Ribeiro et al., 2000; Vale et al., 2012).

The seven CSRs and the catalytic residues were found in most *L. longipalpis* α-amylases and α-glucosidases. We also identified three conserved calcium-binding sites in the β3 sheet (CSR I), loop 3 (CSR V), and β4 sheet (CSR II) in the complete α-amylases sequences (Supplementary Table 5 and Table 1). In general, four residues function as the calcium-binding site, and three of them are conserved (Machius et al., 1995). Calcium preserves the structural integrity of the active site, maintaining proper folding (Buisson et al., 1987). Although some of the α-amylases identified did not demonstrate the typical catalytic residues (LlAamyB1, LlAamyB4, LlAamyB6), it is not possible to confirm whether these proteins are catalytically inactive or function with a distinct catalytic mechanism from the classical one described for GH13 proteins.

The chloride binding site is a characteristic of some α-amylases, and chloride works as an allosteric activator of the enzyme. In most *L. longipalpis* α-amylases, the chloride binding residues were identified. The dependence of *L. longipalpis* larvae α-amylases on chloride ions was demonstrated (Vale et al., 2012). However, LlAamyB1 and LlAamyB4 have a substitution in the asparagine residue (Table 1). Some α-amylases are described as chloride-independent, as α-amylases from plants, fungi and most bacteria (Terra and Ferreira, 1994). In this respect, *L. longipalpis* amylases deserve a more detailed biochemical characterization to clarify their kinetical properties.

Regarding GH31, the phylogenetic analysis of GH31 sequences together with the expression analysis suggest that, at least for the neutral α-glucosidase, this enzyme may play its canonical role in the metabolism of *L. longipalpis*. The neutral α-glucosidases, described as glucosidase II in mammals, are soluble enzymes present in the endoplasmic reticulum. They participate in the quality control of glycoprotein folding, catalyzing the hydrolysis of glucose residues of oligosaccharides bound to peptides (Trombetta et al., 1996, 2001; Porath et al., 2016). These proteins are composed of one catalytic α-subunit and the non-catalytic β-subunit, comprising an ER retention signal (HDEL) at the C-terminal position. In *L. longipalpis*, the LlNAglu1 represents the catalytic α-subunit of the neutral α-glucosidase, being homologous to the α-glucosidase II in mammals. The correct catalytic residues were identified for LlNAglu1, with the nucleophile catalytic inside the domain WNDMNE and the general acid/base aspartate positioned on the β6 sheet (Lee et al., 2001; Lovering et al., 2005; Ernst et al., 2006; Sim et al., 2008). In insects, neutral α-glucosidases have been identified by bioinformatics approaches, but no biochemical or structural studies have been carried until now.

The *L. longipalpis* lysosomal α-glucosidase showed a truncated sequence but was predicted based on the grouping in the phylogenetic tree with other enzymes from mosquito species. Proteins from mosquitoes present the TREFOIL and the NtCtMGAM domains that are typical of acid α-glucosidases (Roig-Zamboni et al., 2017). Since the classical catalytic residues were not identified in the *L. longipalpis* sequence, and the expression levels for this enzyme were low, it is plausible to assume that this represents a protein that has lost its enzymatic activity during evolution. The family Drosophilidae (*D. melanogaster* and *D. ananassae*) has no lysosomal α-glucosidase, while these enzymes in mosquitoes lacked the catalytic residues. This pattern might indicate the absence of glycogen degradation inside lysosomes in these insects, suggesting that the glycogen debranching enzyme mainly performs the mobilization of glycogen reserves in the cytosol, in association with other enzymes.

The NET37 proteins are not well described, especially in insects. They were recently characterized in mammals as membrane proteins anchored in the nuclear envelope with the catalytic domain facing the endoplasmic reticulum (Chen et al., 2006; Datta et al., 2009). These proteins have a transmembrane domain in the N-terminal region and a catalytic C-terminal region belonging to the GH31. *Lutzomyia longipalpis* NET37 proteins present the same structural organization (Figure 1C). NET proteins with different functions have been described. NET37 is required for myogenic differentiation of C2C12 cells (mouse myoblast cell line), with higher expression in the skeletal muscle of adult mouse. This activity is dependent on the catalytic site of the glycosidase motif (Datta et al., 2009). Due to the general lack of knowledge about these proteins, it is not possible yet to suppose why their expression is affected by blood-feeding in adult female sandflies.

In summary, the genome of *L. longipalpis* presented several genes with sequences belonging to GH13 and GH31. The putative function of these genes seemed to be conserved, including enzymes involved in sugar metabolism, storage and mobilization of energetic glycogen reserves, and also proteins involved in amino acid transport, N-glycosylation quality control and myogenesis regulation. The canonical role of α-amylases and α-glucosidases is the digestion of plant carbohydrates, but in *L. longipalpis*, these enzymes seemed to be recruited to recognize a wider variety of dietary carbohydrates, including molecules that are present in blood. Our data suggested that genes LLOJ000566 (LlAglu1), LLOJ008156 (LlAglu2), and LLOJ002257 (LlAglu3) are the most probable candidates to be responsible for the previously described α-glucosidade activities produced by *L. longipalpis* adult females after sugar or blood meals. Besides that, it is noteworthy that different α-glucosidade activities, in this insect, are probably regulated by different mechanisms, transcriptional and post-transcriptional, after blood or sugar feeding. It is also important to realize that GH13 and GH31 genes showed a very diverse array of tissue and nutritional expression patterns, emphasizing the complexity of carbohydrate metabolism in insects.

Additionally, new functions may have arisen for these glycosidases, in coherence with an expansion in α-amylase gene family, induction of α-glucosidases after blood-feeding, and downregulation in *Leishmania mexicana* exposed females. To elucidate the roles of α-amylases and α-glucosidases, including the interaction of sandflies with the *Leishmania* parasite, a more detailed analysis of their biochemical features and mechanisms of expression must be pursued.

## Data Availability Statement

The raw data supporting the conclusions of this article will be made available by the authors, without undue reservation.

## Author Contributions

SC, PB, RD, and FG: conception and design of the work. SC: obtainment of experimental data. SC and FG: data analysis. SC, PB, RD, and FG: writing and revision of the manuscript. All authors have read and approved the final version.

## Conflict of Interest

The authors declare that the research was conducted in the absence of any commercial or financial relationships that could be construed as a potential conflict of interest.

## Footnotes

1 http://www.vectorbase.org

2 http://flybase.org/

3 https://www.ncbi.nlm.nih.gov/

4 https://prosite.expasy.org/mydomains/

5 http://www.vectorbase.org

6 http://flybase.org/

7 http://www.premierbiosoft.com/qpcr/index.html

8 http://www.idtdna.com/UNAFold?

## Supplementary Material

Supplementary Figure 1 Schematic diagram of exon and intron composition of *L. longipalpis* genes belonging to GH13 and GH31. Sequences were retrieved from Vector Base (Jacobina strain, LlonJ1.4 geneset, June 2017). Green boxes and lines represent exons and introns, dotted lines and boxes represent missing parts in incomplete sequences. For incomplete sequences, structures were designed based on homology with orthologous sequences.

Supplementary Table 1 Identifiers and accession numbers of insect GH13 protein sequences used for the phylogenetic tree of Figure 2.

Supplementary Table 2 Identifiers and accession numbers of insect GH31 protein sequences used for the phylogenetic tree of Figure 3.

Supplementary Table 3 List and sequences of the RT-qPCR primers used for PCR-amplification of cDNA sequences codifying for proteins belonging to GH13 and GH31 of *L. longipalpis*.

Supplementary Table 4 Modifications on sequences from GH13 and GH31 retrieved from vector base.

Supplementary Table 5 Manual annotation of *L. longipalpis* GH13 sequences retrieved from Vector Base (*L. longipalpis*, Jacobina strain, LlonJ1.4 geneset, June 2017). Sequences were considered complete when initial methionine, correct exon/intron junction, stop codon were identified, and exon structures were complete based on homology with orthologous genes. For incomplete proteins some N and O-glycosylation sites are missing. In transmembrane proteins, only extracellular domains were considered to be N-glycosylated. Non-canonical catalytic residues are highlighted. ND, Not Determined.

Supplementary Table 6 Manual annotation of *L. longipalpis* GH31 sequences retrieved from Vector Base (*L. longipalpis*, Jacobina strain, LlonJ1.4 gene set, June 2017). Sequences were considered complete when initial methionine, correct exon/intron junction, stop codon were identified, and exon structures were complete based on homology with orthologous genes. For incomplete proteins some N and O-glycosylation sites are missing In transmembrane proteins, only extracellular domains were considered as N-glycosylated. Non-canonical catalytic residues are highlighted. ND, Not Determined.

Supplementary File 1 Exon and introns sequences of *L. longipalpis* genes belonging to GH13 and GH31 after sequence curation.

Supplementary File 2 Protein sequences of *L. longipalpis* and *P. papatasi* belonging to GH13 and GH31 after sequence curation.

## Abbreviations

GH: glycoside hydrolases
CSRs: conserved regions
glycosidase NET37: nuclear envelope transmembrane glycosidase 37
SF: sugar-fed
BF: blood-fed
NF: newly hatched females
RB: rest of the body
NBAThc: neutral and basic amino acid transport protein heavy chain
CD98hc: cluster of differentiation 98 heavy chain
GT: gluconotransferase
GDE: glycogen debranching enzymes
LlAamy: *Lutzomyia longipalpis* α-amylase
LlAglu: *Lutzomyia longipalpis* α-glucosidase
LlNBAThc: *Lutzomyia longipalpis* neutral and basic amino acid transport protein heavy chain
LlCD98hc: *Lutzomyia longipalpis* cluster of differentiation 98 heavy chain
LlAGB: *Lutzomyia longipalpis* 1,4-alpha-glucan-branching enzyme
LlGDE: *Lutzomyia longipalpis* glycogen debranching enzyme
LlGlyMyo: *Lutzomyia longipalpis* glycosidase NET37
LlLysAglu: *Lutzomyia longipalpis* lysosomal alpha-glucosidase
LlNAglu: *Lutzomyia longipalpis* neutral alpha-glucosidase
PpAgluA2: *Phlebotomus papatasi* α-glucosidase A2
CqMal1: *Culex quinquefasciatus* α-glucosidase 1.
