# Supplementary material for: Characterization of Glycoside Hydrolase Families 13 and 31 Reveals Expansion and Diversification of α-Amylase Genes in the Phlebotomine Lutzomyia longipalpis and Modulation of Sandfly Glycosidase Activities by Leishmania Infection
Source: Front Physiol. 2021 Apr 9;12:635633. doi: 10.3389/fphys.2021.635633 (PMC8063059; doi:10.3389/fphys.2021.635633)
Supplement: Supplementary Figure 1 — Schematic diagram of exon and intron composition of L. longipalpis genes belonging to GH13 and GH31. Sequences were retrieved from Vector Base (Jacobina strain, LlonJ1.4 geneset, June 2017). Green boxes and lines represent exons and introns, dotted lines and boxes represent missing parts in incomplete sequences. For incomplete sequences, structures were designed based on homology with orthologous sequences. [file Data_Sheet_3.pdf]

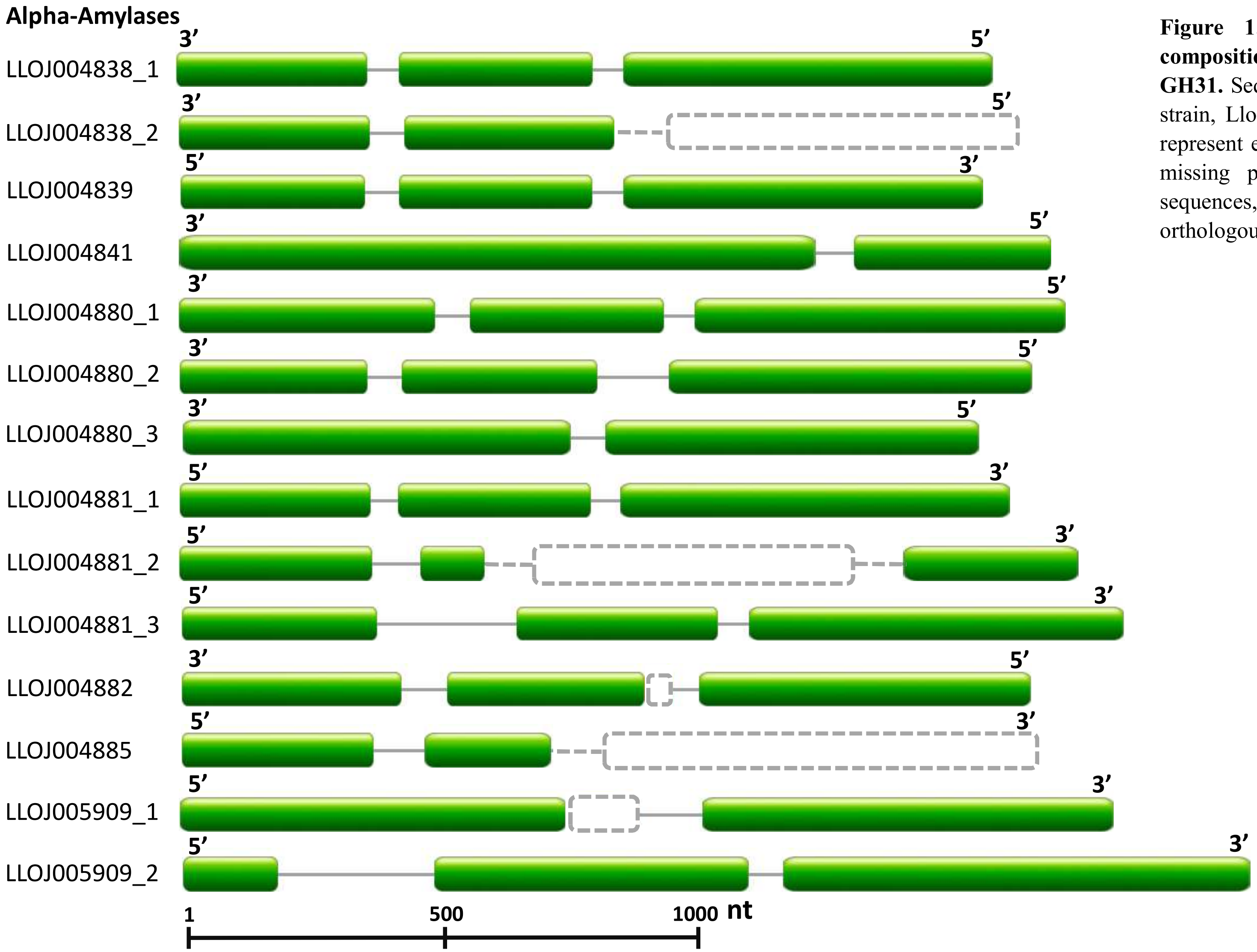

Figure 1. Schematic diagram of exon and intron composition of *L. longipalpis* genes belonging to GH13 and GH31. Sequences were retrieved from Vector Base (Jacobina strain, LlonJ1.4 geneset, June 2017). Green boxes and lines represent exons and introns, dotted lines and boxes represent missing parts in incomplete sequences. For incomplete sequences, structures were designed based on homology with orthologous sequences.

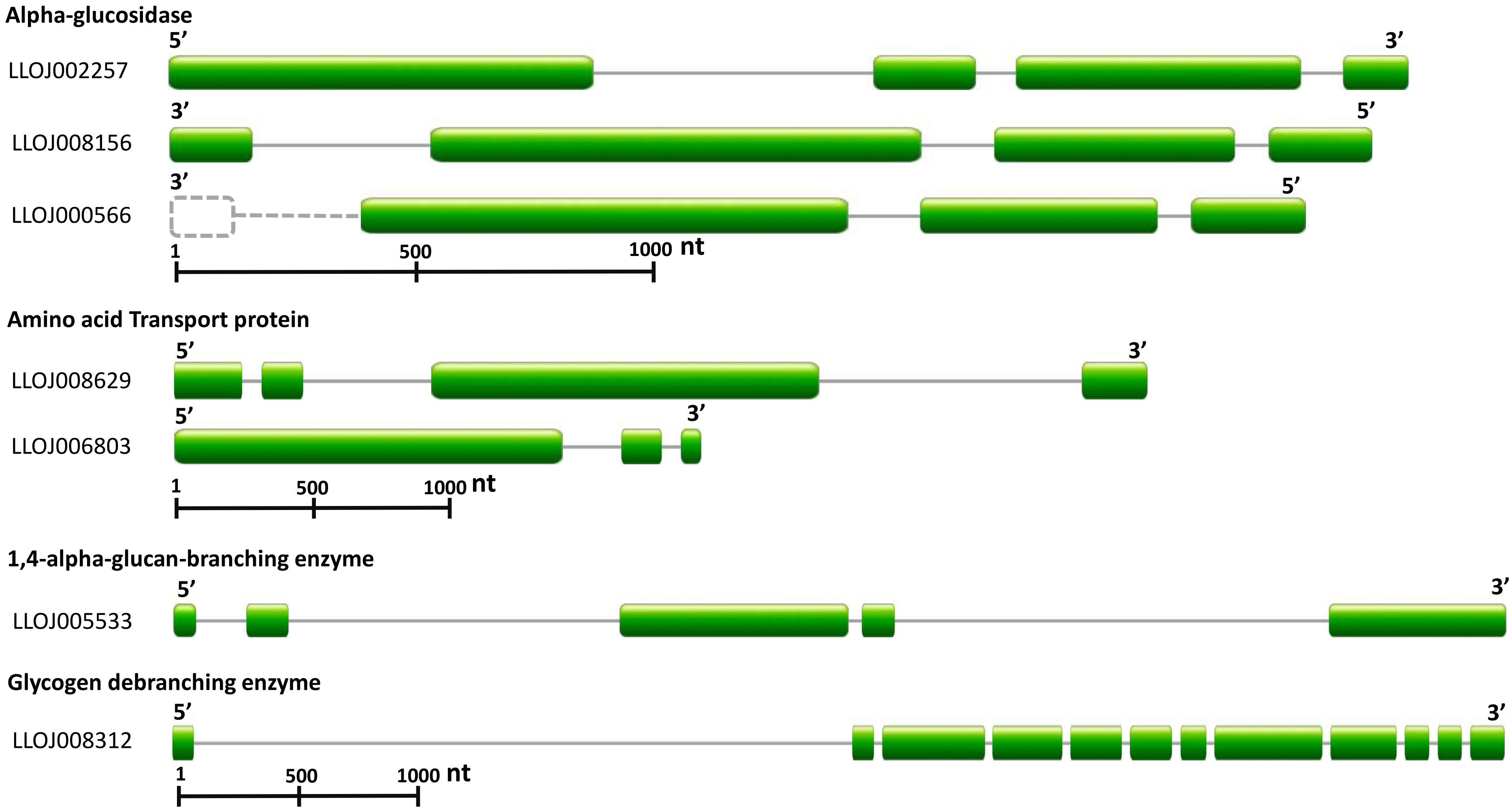

Alpha-glucosidase
5'
3'
LLOJ002257
3'
5'
LLOJ008156
3'
5'
LLOJ000566
1
500
1000 nt
Amino acid Transport protein
5'
3'
LLOJ008629
5'
3'
LLOJ006803
1
500
1000 nt
1,4-alpha-glucan-branching enzyme
5'
3'
LLOJ005533
Glycogen debranching enzyme
5'
3'
LLOJ008312
1
500
1000 nt

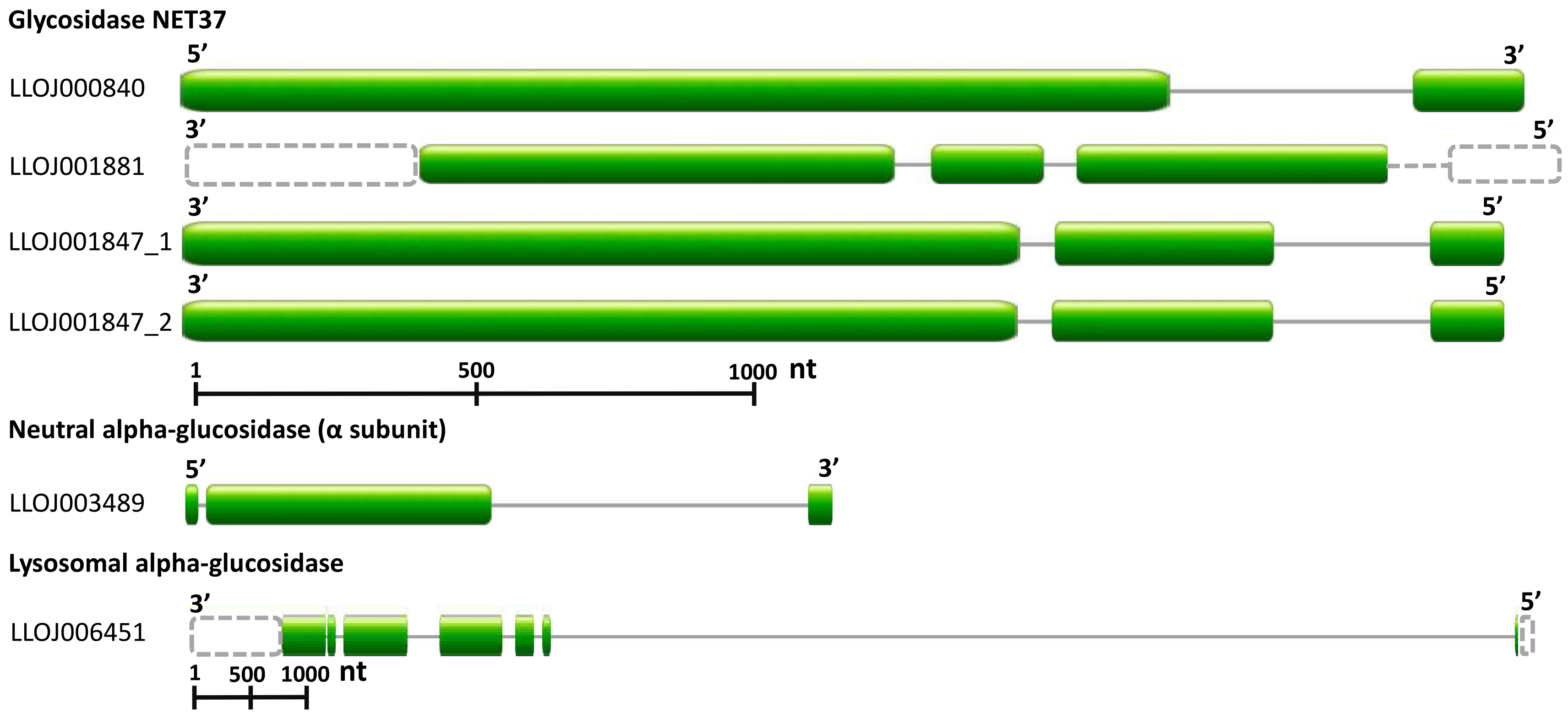
Glycosidase NET37
5'
3'
LLOJ000840
3'
5'
LLOJ001881
3'
5'
LLOJ001847_1
3'
5'
LLOJ001847_2
1
500
1000 nt
Neutral alpha-glucosidase (α subunit)
5'
3'
LLOJ003489
Lysosomal alpha-glucosidase
3'
5'
LLOJ006451
1
500
1000 nt
