# Supplementary material for: Characterization of Glycoside Hydrolase Families 13 and 31 Reveals Expansion and Diversification of α-Amylase Genes in the Phlebotomine Lutzomyia longipalpis and Modulation of Sandfly Glycosidase Activities by Leishmania Infection
Source: Front Physiol. 2021 Apr 9;12:635633. doi: 10.3389/fphys.2021.635633 (PMC8063059; doi:10.3389/fphys.2021.635633)
Supplement: Supplementary Table 5 — Manual annotation of L. longipalpis GH13 sequences retrieved from Vector Base (L. longipalpis, Jacobina strain, LlonJ1.4 geneset, June 2017). Sequences were considered complete when initial methionine, correct exon/intron junction, stop codon were identified, and exon structures were complete based on homology with orthologous genes. For incomplete proteins some N and O-glycosylation sites are missing. In transmembrane proteins, only extracellular domains were considered to be N-glycosylated. Non-canonical catalytic residues are highlighted. ND, Not Determined. [file Table_5.docx]

**Table 5.** **Manual annotation of *L. longipalpis* GH13 sequences retrieved from Vector Base** (*L. longipalpis*, Jacobina strain, LlonJ1.4 geneset, June 2017). Sequences were considered complete when initial methionine, correct exon/intron junction and stop codon were identified, and when exon structures were complete based on homology with orthologous genes. For incomplete proteins some N and O-glycosylation sites are missing. In transmembrane proteins, only extracellular domains were considered to be N-glycosylated. Non canonical catalytic residues are highlighted. N.D.- Not Determined.

| **Gene** | **Strand** | **Status** | **Best Hit** | **E value** | **Signal Peptide** | **TM Domain** | **N-Glycosilation Position** | **O-Glycosilation Position** | **Mw (kDa)** | **pI** | **DeepLoc** | **Catalytic site (InterPro)** | **Ca Binding Site (InterPro)** |
| --- | --- | --- | --- | --- | --- | --- | --- | --- | --- | --- | --- | --- | --- |
| LLOJ004838_1 (LlAamyA1) | Reverse | Complete | Alpha-amylase A (AMYA_DROMA - P54215) -  *Drosophila mauritiana* | 0.00E+00 | 1-17 | No | 410 | 130, 131, 145, 367, 369 | 54.6 | 6.43 | Extracellular - Soluble | D204, E241, D306 | N115, D174, H208 |
|  |  |  |  |  |  |  |  |  |  |  |  |  |  |
|  |  |  |  |  |  |  |  |  |  |  |  |  |  |
| LLOJ004838_2 (LlAamyA2) | Reverse | Incomplete (Truncated 5') | Alpha-amylase B (AMYB_DROYA - Q9BN01) - *Drosophila yakuba* | 4.40E-92 | 1-17 | No | 161 | 131, 135, 137, 139 | N.D. | N.D. | Extracellular - Soluble | D206, E243,  N.D. | N.D. |
|  |  |  |  |  |  |  |  |  |  |  |  |  |  |
|  |  |  |  |  |  |  |  |  |  |  |  |  |  |
| LLOJ004839 (LlAamyA3) | Forward | Complete | Alpha-amylase 1 (AM4N_DROAN - Q23835) - *Drosophila ananassae* | 0.00E+00 | 1-15 | No | 280 | 129, 130, 135, 137, 365 | 55.2 | 5.06 | Extracellular - Soluble | D203, E240, D308 | N115, D173, H207 |
|  |  |  |  |  |  |  |  |  |  |  |  |  |  |
|  |  |  |  |  |  |  |  |  |  |  |  |  |  |
| LLOJ004841 (LlAamyA4) | Reverse | Complete | Alpha-amilase 2 (AMY2_DROAN - O18345) – *Drosophila ananassae* | 0.00E+00 | 1-16 | No | No | 134, 365, 367 | 56.0 | 5.51 | Extracellular - Soluble | D203, E240, D304 | N114, D173, H207 |
|  |  |  |  |  |  |  |  |  |  |  |  |  |  |
|  |  |  |  |  |  |  |  |  |  |  |  |  |  |
| LLOJ004880_1 (LlAamyB1) | Reverse | Complete | Alpha-amylase 2 (AMY2_DROAN - O18345) *Drosophila ananassae* | 1.00E-176 | 1-16 | No | 277 | 130, 131, 136, 138, 146, 152, 367 | 55.3 | 4.61 | Extracellular - Soluble | D204, D241, **Q307** | N116, D174, **N208** |
|  |  |  |  |  |  |  |  |  |  |  |  |  |  |
|  |  |  |  |  |  |  |  |  |  |  |  |  |  |
| LLOJ004880_2 (LlAamyB2) | Reverse | Complete | Alpha-amylase 1 (AMY1_DROAN - Q23835) - *Drosophila ananassae* | 0.00E+00 | 1-16 | No | 297, 406, 438 | 130, 131, 136, 151, 370, 372 | 56.2 | 5.89 | Extracellular - Soluble | D204, E244, D309 | N116, D174, H208 |
|  |  |  |  |  |  |  |  |  |  |  |  |  |  |
|  |  |  |  |  |  |  |  |  |  |  |  |  |  |
| LLOJ004880_3 (LlAamyB3) | Reverse | Complete | Alpha-amylase A (AMYA_DROMA - P54215) - *Drosophila mauritiana* | 0.00E+00 | 1-21 | No | No | 134 | 55.9 | 5.09 | Extracellular - Soluble | D208, E245, D309 | N119, D178, H212 |
|  |  |  |  |  |  |  |  |  |  |  |  |  |  |
|  |  |  |  |  |  |  |  |  |  |  |  |  |  |
| LLOJ004881_1 (LlAamyB4) | Forward | Complete | Alpha-amylase A (AMYA_DROME - P08144) - *Drosophila melanogaster* | 1.50E-169 | 1-17 | No | 177 | 131, 132, 137, 139, 146, 147, 153, 366, 368 | 55.3 | 4.98 | Extracellular - Soluble | **N205**, D242, D307 | N117, D175, **F209** |
|  |  |  |  |  |  |  |  |  |  |  |  |  |  |
|  |  |  |  |  |  |  |  |  |  |  |  |  |  |
| LLOJ004881_2 (LlAamyB5) | Forward | Incomplete | Alpha-amylase-related protein (AMYR_DROAN - O18344) - *Drosophila ananassae* | 9.90E-50 | 1-16 | No | N.D. | 131,132, 137, 139 | N.D. | N.D. | Extracellular - Soluble | N. D. | N. D. |
|  |  |  |  |  |  |  |  |  |  |  |  |  |  |
|  |  |  |  |  |  |  |  |  |  |  |  |  |  |
| LLOJ004881_3 (LlAamyB6) | Forward | Complete | Alpha-amylase 4N (AM4N_DROAN - Q23834) - *Drosophila ananassae* | 4.00E-164 | 1-16 | No | 269 | 131, 132, 137, 139, 147, 371 | 55.9 | 4.47 | Extracellular - Soluble | **F208**, D245, **Q311** | N117, D178, H212 |
|  |  |  |  |  |  |  |  |  |  |  |  |  |  |
|  |  |  |  |  |  |  |  |  |  |  |  |  |  |
| LLOJ004882 (LlAamyB7) | Reverse | Incomplete | Alpha-amylase 1 (AM4N_DROAN - Q23835) - *Drosophila ananassae* | 0.00E+00 | 1-17 | No | 161, 432 | 131, 135, 137, 139, 136 | N.D. | N.D. | Extracellular - Soluble | D206, E243, D305 | N117, D176, H210 |
|  |  |  |  |  |  |  |  |  |  |  |  |  |  |
|  |  |  |  |  |  |  |  |  |  |  |  |  |  |
| LLOJ004885 (LlAamyB8) | Forward | Incomplete (Truncated 3') | Alpha-amylase-related protein (AMYR_DROAN - O18344) - *Drosophila ananassae* | 2.60E-63 | 1-16 | No | N.D. | 131, 132, 137, 139, 147 | N.D. | N.D. | Extracellular - Soluble | N.D. | N.D. |
|  |  |  |  |  |  |  |  |  |  |  |  |  |  |
|  |  |  |  |  |  |  |  |  |  |  |  |  |  |
| LLOJ005909_1 (LlAamyC1) | Forward | Incomplete | Alpha-amylase A (AMYA_DROMA - P54215) -  *Drosophila mauritiana* | 9.70E-174 | 1-21 | No | 401 | 134, 370 | N.D. | N.D. | Extracellular - Soluble | D208, N.D.,  D309 | N119, D178, H212 |
|  |  |  |  |  |  |  |  |  |  |  |  |  |  |
|  |  |  |  |  |  |  |  |  |  |  |  |  |  |
| LLOJ005909_2 (LlAamyC2) | Forward | Complete | Alpha-amylase-related protein (AMYR_DROAN - O18344) - *Drosophila ananassae* | 0.00E+00 | 1-18 | No | 179, 228, 414 | 130, 133 | 55.9 | 6.18 | Extracellular - Soluble | D207, E244, D309 | N116, D177, H211 |
|  |  |  |  |  |  |  |  |  |  |  |  |  |  |
|  |  |  |  |  |  |  |  |  |  |  |  |  |  |
| LLOJ000566 (LlAglu1) | Reverse | Incomplete (Truncated 3') | Maltase A1 (MAL1_DROME - P07190) – *D. Melanogaster* | 0.00E+00 | N.D. | Yes Position 581 - 600 | N. D. | 159, 241, 427, 440, 450, 547 | N.D. | N.D. | N.D. | D216, E290, D357 | No |
|  |  |  |  |  |  |  |  |  |  |  |  |  |  |
|  |  |  |  |  |  |  |  |  |  |  |  |  |  |
| LLOJ008156 (LlAglu2) | Reverse | Complete | Maltase A1 (MAL1_DROME - P07190) – *D. Melanogaster* | 0.00E+00 | 1-17 | Yes Position 581 - 600 | 116 | 241, 427, 440, 450, 547 | 69.6 | 5.6 | Cell Membrane - Membrane | D216, E290, D357 | No |
|  |  |  |  |  |  |  |  |  |  |  |  |  |  |
|  |  |  |  |  |  |  |  |  |  |  |  |  |  |
| LLOJ002257 (LlAglu3) | Forward | Complete | Maltase 2 (MAL2_DROVI - O16099) - *Drosophila virilis* | 0.00E+00 | 1-16 | Yes Position 588 - 610 | 101, 136, 308, 432 | 246, 459, 460, 463 | 70.9 | 4.7 | Cell Membrane - Membrane | D236, E304, D375 | No |
|  |  |  |  |  |  |  |  |  |  |  |  |  |  |
|  |  |  |  |  |  |  |  |  |  |  |  |  |  |
| LLOJ008629 (LlNBAThc) | Forward | Complete | Neutral and basic amino acid transport protein rBAT (SLC31_RAT-Q64319) - *Rattus norvegicus* | 9.60E-21 | No | Yes Position 157 - 176 | No | 300, 303, 305, 494 | 69.7 | 4.93 | Endoplasmic reticulum - Membrane | **K345,** E384,  N.D. | No |
|  |  |  |  |  |  |  |  |  |  |  |  |  |  |
|  |  |  |  |  |  |  |  |  |  |  |  |  |  |
| LLOJ006803 (LlCD98hc) | Forward | Complete | 4F2 cell-surface antigen heavy chain (4F2_RAT-Q64319) - *Rattus norvegicus* | 5.40E-19 | No | Yes Position 151 - 173 | No | 283, 367, 385, 528, 590 | 69.9 | 5.60 | Endoplasmic reticulum, Membrane | **A350,** N.D.,  N.D. | No |
|  |  |  |  |  |  |  |  |  |  |  |  |  |  |
|  |  |  |  |  |  |  |  |  |  |  |  |  |  |
| LLOJ005533 (LlAGB1) | Forward | Complete | 1,4-alpha-glucan-branching enzyme - XP_017959984.1 - *Drosophila navojoa* | 0.00E+00 | No | No | No | No | 79.8 | 5.82 | Cytoplasm, Soluble | D347, E402, D471 | No |
|  |  |  |  |  |  |  |  |  |  |  |  |  |  |
|  |  |  |  |  |  |  |  |  |  |  |  |  |  |
| LLOJ008312 (LlGDE1) | Forward | Complete | Glycogen debranching enzyme - XP_012157586.1 - *Ceratitis capitata* | 0.00E+00 | No | No | No | 248, 250, 272, 344, 1066, 1069, 1177, 1298, 1307, 1311, 1312, 1316 | 172.6 | 6.26 | Cytoplasm, Soluble | D548, E577, D649,  D1282,  E1515 | No |
|  |  |  |  |  |  |  |  |  |  |  |  |  |  |
|  |  |  |  |  |  |  |  |  |  |  |  |  |  |
|  |  |  |  |  |  |  |  |  |  |  |  |  |  |
