# Supplementary material for: Characterization of Glycoside Hydrolase Families 13 and 31 Reveals Expansion and Diversification of α-Amylase Genes in the Phlebotomine Lutzomyia longipalpis and Modulation of Sandfly Glycosidase Activities by Leishmania Infection
Source: Front Physiol. 2021 Apr 9;12:635633. doi: 10.3389/fphys.2021.635633 (PMC8063059; doi:10.3389/fphys.2021.635633)
Supplement: Supplementary Table 6 — Manual annotation of L. longipalpis GH31 sequences retrieved from Vector Base (L. longipalpis, Jacobina strain, LlonJ1.4 gene set, June 2017). Sequences were considered complete when initial methionine, correct exon/intron junction, stop codon were identified, and exon structures were complete based on homology with orthologous genes. For incomplete proteins some N and O-glycosylation sites are missing In transmembrane proteins, only extracellular domains were considered as N-glycosylated. Non-canonical catalytic residues are highlighted. ND, Not Determined. [file Table_6.docx]

**Table 6**. **Manual annotation of *L. longipalpis* GH31 sequences retrieved from Vector Base** (*L. longipalpis*, Jacobina strain, LlonJ1.4 gene set, June 2017). Sequences were considered complete when initial methionine, correct exon/intron junction and stop codon were identified, and when exon structures were complete based on homology with orthologous genes. For incomplete proteins some N and O-glycosylation sites are missing In transmembrane proteins, only extracellular domains were considered as N-glycosylated. Non canonical catalytic residues are highlighted. N.D.- Not Determined.

| **Gene** | **Strand** | **Status** | **Best Hit** | **E value** | **Signal Peptide** | **TM Domain** | **N-Glycosilation Position** | **O-Glycosilation Position** | **Mw (kDa)** | **pI** | **DeepLoc** | **Catalytic site** |
| --- | --- | --- | --- | --- | --- | --- | --- | --- | --- | --- | --- | --- |
| LLOJ001847_1 (LlGlyMyo1) | Reverse | Complete | Myogenesis-regulating glycosidase (MYORG_MOUSE -Q69ZQ1) - *Mus musculus* | 3.60E-75 | No | Yes Position 20-39 | No | 86 | 79.8 | 8.88 | Golgi apparatus, Membrane | D437, **P484** |
|  |  |  |  |  |  |  |  |  |  |  |  |  |
|  |  |  |  |  |  |  |  |  |  |  |  |  |
| LLOJ001847_2 (LlGlyMyo2) | Reverse | Complete | Myogenesis-regulating glycosidase (MYORG_MOUSE -Q69ZQ1) - *Mus musculus* | 2.40E-72 | No | Yes Position 13-35 | No | No | 79.3 | 8.18 | Golgi apparatus, Membrane | D427, **P485** |
|  |  |  |  |  |  |  |  |  |  |  |  |  |
|  |  |  |  |  |  |  |  |  |  |  |  |  |
| LLOJ001881 (LlGlyMyo3) | Reverse | Incomplete (Truncated 5' and 3') | Myogenesis-regulating glycosidase (MYORG_HUMAN - Q6NSJ0)  Homo sapiens | 4.40E-83 | N.D. | No | N.D. | No | N.D. | N.D. | N.D. | D401, D460 |
|  |  |  |  |  |  |  |  |  |  |  |  |  |
|  |  |  |  |  |  |  |  |  |  |  |  |  |
| LLOJ000840 (LlGlyMyo4) | Forward | Complete | Myogenesis-regulating glycosidase (MYORG_HUMAN - Q6NSJ0)  Homo sapiens | 4.50E-65 | No | Yes Position 21 - 43 | No | No | 76.3 | 7.13 | Lysosome/Vacuole, Membrane | E408, D467 |
|  |  |  |  |  |  |  |  |  |  |  |  |  |
|  |  |  |  |  |  |  |  |  |  |  |  |  |
| LLOJ006451 (LlLysAglu1) | Reverse | Incomplete (Truncated 5' and 3') | Lysosomal alpha-glucosidase (LYAG_RAT- Q6P7A9) - *Rattus norvegicus* | 2.00E-17 | N.D. | No | N.D. | No | N.D. | N.D. | Lysosome/Vacuole, Soluble | D492, **Q570** |
|  |  |  |  |  |  |  |  |  |  |  |  |  |
|  |  |  |  |  |  |  |  |  |  |  |  |  |
| LLOJ003489 (LlNAglu1) | Forward | Complete | Neutral alpha-glucosidase AB(B0WQR9_CULQU) - *Culex quinquefasciatus* | 0.00E+00 | 1-18 | No | 114, 435, 891 | 323, 325 | 105.8 | 5.62 | Endoplasmic reticulum, Soluble | D522, D598 |
|  |  |  |  |  |  |  |  |  |  |  |  |  |
|  |  |  |  |  |  |  |  |  |  |  |  |  |
