# Supplementary material for: Characterization of Glycoside Hydrolase Families 13 and 31 Reveals Expansion and Diversification of α-Amylase Genes in the Phlebotomine Lutzomyia longipalpis and Modulation of Sandfly Glycosidase Activities by Leishmania Infection
Source: Front Physiol. 2021 Apr 9;12:635633. doi: 10.3389/fphys.2021.635633 (PMC8063059; doi:10.3389/fphys.2021.635633)
Supplement: Supplementary File 1 — Exon and introns sequences of L. longipalpis genes belonging to GH13 and GH31 after sequence curation. [file Data_Sheet_1.pdf]

File 1. Exon and introns sequences of *L. longipalpis* genes belonging to GH13 and GH31 after sequence curation. The Xs represent missing nucleotides in incomplete sequences. For missing introns sequences, size was estimated based on homology with orthologous complete genes.

```
L. longipalpis Glycoside Hydrolases from family 13
Alpha-Amylase

> LLOJ004838_1/ LlAamyA1
>LLOTMP004838_1-E1 exon: PUTATIVE_protein_coding
ATGAAGTTCTGTGTTTTCCTCCTATTGACCTTTGTGGCCCTGGCCAGGGGTCAATTTGATCCGCATTTTG
TTTCCGGACGCAGTGGAATTGTTCACCTTTTTGAGTGGAAATGGGAAGATATTGCAGCTGAGTGCGAAAG
ATTCTTGGGTCCAAATGGTTTTGCTGGAGTCCAAGTTTCACCACCAAATGAGAATGTTATCGTAACGAAT
CGTCCATGGTGGGAACGTTATCAGCCCATATCTTATAGGATTGTAACGAGATCAGGGAATGAGCAACAAT
TCGCAAACATGGTCTACAGATGCAACAATGTTGGTGTTCGCATCTACCCTGATATTGTGATCAATCACAT
GGCTGCTTCTCATCCAGTTATGATG
>LLOTMP004838_1-E2 exon: PUTATIVE_protein_coding
GGAACTGGAGGGTCTACTGCTAACGTTGGAAACCGAGACTACCCAGCTGTGCCCTACAGCATCCATGATT
TCAACCCTTCATGCCCTATTACCAACTACAATGATCGCTATCAAGTGAGGAATTGTGAGCTAGTAGGCTT
GCCAGATCTCAATCAAGGAGTTGCCTGGGTCAGGGATAGAATTGTTGAGTATTTGGACAATCTTGTGGCA
CTGGGTGTTGCTGGATTCCGCGTTGATGCAGCCAAGCACATGTGGCCAGGTGATCTGGAGATCATCTATG
GACGCGTAAGAGAACTGAATGTAGTTCACGGCTTCCCAGTTCATGCTAGACCATTCTTCTTCCAAGAAGT
CATTGACTTGGGCGGTGAAGCTATATCCAAG
>LLOTMP004838_1-E3 exon: PUTATIVE_protein_coding
AACGAATACACGGGCTTTGGTGTTGTGACTGAATTCAAATTTTCCGCTGAAATTGGTCGTGTCTTCCGTG
GCAATGATCTTCTGCATCACCTGTCCAATTGGGGTGAAGCATGGGGCTTCCTTCCATCCAATCGTGCTCT
GGTGTTTGTTGAGAATCACGACAATGAACGCGGACACGGAGCTGGAGGAGACCAAATCCTAACCTACAAA
AATGCTAAACAATACAAAATGGCCGTTGCTTTTACACTTGCGCATCCATTCGGAATTCCACGTATCATGT
CCTCCTTTGCGTTCACCAACACCGAAATTGGTCCACCAATGGATGGGAATCAGAATATTATTTCTCCCAG
TATTAATGCCGATGGTACGTGTGGCAATGGATGGGTGTGCCAGCACAGATGGAGGCAGATCTTCAATATG
ATTCGCTTCCGGAATGAAGCAGGAACAGCTGGACTTACAAATTGGTGGTCAAATGGAAGCCAGCAGATTG
CTTTTGCACGTGCCGGTCGGGCTTTTGTTGCCTTCAACAATCAAGGATCCAACATGAATGTAAATCTTCA
GACTTCCCTTCCTGGTGGAACGTACTGCGATGTTATCTCGGGGAATGTTTCCGGAGGATCATGTTCCGGA
AAGACCGTTACAGTCAATGGAGACGGAACTGCCAATATAGTCATTGGAGCAGCTGAAGAAGATGGCGTGC
TTGCTATTCACGTTGGTGCAAGATTGTA
>LLOTMP004838_1-Intron 1: PUTATIVE_protein_coding
TGAGTGTTTGTTTACGCATAAGAAAATGAGGAAGGAGATTAAAAGTTATTGTTTTTTTTGTAGT
>LLOTMP004838_1-Intron 2: PUTATIVE_protein_coding
TAAGGACAACAACGTCTTTCCTCCTTCCTTGCTCCTAATTCTTTGACTTTCTTTTCCTAGG

> LLOJ004838_2/ LlAamyA2
>LLOTMP004838_2-E1 exon: PUTATIVE_protein_coding
ATGAAAATCTTATGGATTTTCTTCTTGGCCATAGTGGCCACTGTTCGTGGTCAATTTGATCCGCACTACG
TTGCAGGTCGTAGTGTGATGGTCTTCCTTATGGACTGGAAGTATGAGGACGTTGCTCTGGAGTGTGAGAG
ATTCCTCGGACCAATGGGCTTTGCTGGTGTTCAGGTTTCAGCTGCTTCTGAGAATGATATTGTTACGAGT
CCACTTCGTCCTTGGTGGGAACGCTATCAAGTTGTTTCCTACAAGATCGGTTCGAGATCTGGAGATGCAG
ACAGTTTTGCAGACATGTGCACCAGATGCAATAACGTTGGGGTACGCGTCTACGTTGATGCCATCTTCAA
CAACATGGGGGGTATGGGGATTATGAA
>LLOTMP004838_2-E2 exon: PUTATIVE_protein_coding
AGGAACAGCTGGATCATTGGGAGATTCAATAACAAGAACCTTCCCTGCTGTCCCCTACACTATTCTAAAT
TTCCACAATCCACCATGTTCGATAACAGACTACAACAACACGCAACAGGTTCGCAATTGCGATCTTGTAG
GTGCTCCGGATCTTGATCAATCTCAACCCTATGTACGAGATAGAATTGTAGGTTATCTGAATCATCTAAC
TTCCCTTGGGTGCGCTGGTTTTCGCATTGATTCAGCCAAGCATATGTGGCCAACGGATCTCCAGGCCATC
TATAGCAGTGTCATAGACTTAAATACAGCCCAAGGATTTCCAGCTGGTAGCAGATCATTCATCTTCCAAG
AAGTCATTGATTCTGGCCATGAAGCAATTAAGAAGTATACTCTGAAAAAAATAGTTTATATTTGA
>LLOTMP004838_2-E3 exon: PUTATIVE_protein_coding
XXXXXXXXXXXXXXXXXXXXXXXXXXXXXXXXXXXXXXXXXXXXXXXXXXXXXXXXXXXXXXXXXXXXXX
XXXXXXXXXXXXXXXXXXXXXXXXXXXXXXXXXXXXXXXXXXXXXXXXXXXXXXXXXXXXXXXXXXXXXX
XXXXXXXXXXXXXXXXXXXXXXXXXXXXXXXXXXXXXXXXXXXXXXXXXXXXXXXXXXXXXXXXXXXXXX
XXXXXXXXXXXXXXXXXXXXXXXXXXXXXXXXXXXXXXXXXXXXXXXXXXXXXXXXXXXXXXXXXXXXXX
```

XXXXXXXXXXXXXXXXXXXXXXXXXXXXXXXXXXXXXXXXXXXXXXXXXXXXXXXXXXXXXXXXXXXXXX
XXXXXXXXXXXXXXXXXXXXXXXXXXXXXXXXXXXXXXXXXXXXXXXXXXXXXXXXXXXXXXXXXXXXXX
XXXXXXXXXXXXXXXXXXXXXXXXXXXXXXXXXXXXXXXXXXXXXXXXXXXXXXXXXXXXXXXXXXXXXX
XXXXXXXXXXXXXXXXXXXXXXXXXXXXXXXXXXXXXXXXXXXXXXXXXXXXXXXXXXXXXXXXXXXXXX
XXXXXXXXXXXXXXXXXXXXXXXXXXXXXXXXXXXXXXXXXXXXXXXXXXXXXXXXXXXXXXXXXXXXXX
XXXXXXXXXXXXXXXXXXXXXXXXXXXXXXXXXXXXXXXXXXXXXXXXXXXXXXXXXXXXXXXXXXXXX
>LLOTMP004838_2-Intron 1: PUTATIVE_protein_coding
GTAAGTGCAGTCATGCAACATCAAGATGATCTGGAGTGTCTGGTCTTTTTTGACAATCTTTCATTACAG
>LLOTMP004838_2-Intron 2: PUTATIVE_protein_coding
XXXXXXXXXXXXXXXXXXXXXXXXXXXXXXXXXXXXXXXXXXXXXXXXXXXXXXXXXXXXXXXXXXXXXX
XXX

>LLOTMP004839/ LlAamyA3
>LLOTMP004839-E1 exon :PUTATIVE_protein_coding
ATGTTCTGGAATCTTTTTTTGACAACGTTGGCCATAGCCCGTGGTCAATTTAACCCAAACTACGTACCAG
GACGTACGGTAATGGTCTTCCTTATGGACTGGAAGTATCAGGATGTCGCATTGGAGTGTGAGAGATTCCT
GGGACCGAAAGGCTTTGGTGGAGTCCAAGTTTCATCTCCAGCTGAGAATGATATTGTTACGGATCCTTTG
CGTCCTTGGTGGGAACGCTATCAAATTATTTCCTACATCATCGGATCGAGGTCTGGAAACGAAGATGATT
TTGCAGACATGTGTTACAGATGCAATAAAGTCGGAGTTCGTATCTACGTTGACGCCATTTTCAATCATAT
GGGGTCTCTACCAAACATG
>LLOTMP004839-E2 exon :PUTATIVE_protein_coding
TTGGAACTGCTGGATCAACAGCAGATGGACCAACTAAGTCCTATCCAGCTGTACCGTATGACGCATCAAA
CTTCCATCCTTCCTGCCCTCTTACTACATTCCAAGTTACCTTCATTGTTCGCAACTGTGAACTGTTTGGT
GCTCCTGATCTTGATCAAGCGCAACCCTATGTCCGTGAGAAAATCAAAGGATATCTCGATCATTTGATTA
CTCTCGGAATAGCTGGTTTTCGCGTTGATTCCGCCAAGCATATGTGGCCTAAAGACCTCAAGGCTATTTA
CAGCAGCCTAAGAGATCTTAATACAGATTTTGGTTTCCCGCCAAAATCTAGGCCTTTCGTTTTTCAAGAA
GTTATCGATGCAGGCGGTGGCTCTGAACCCATTAACAA
>LLOTMP004839-E3 exon :PUTATIVE_protein_coding
GTATGAATATTCACCAATTGGAGTTGTTACTGAATTCCTCTACTCATACATTTTGACTGACTTCTTCCGT
GGCAATCGAAATTTGAGCGATTTAGCCACGTGGGGTCAATCTGAGTGGAAGTTCCTTCCATCGAAGGCTG
CTCTGGTGTTTGTTGAGAACCACGACAACGAGCGGGCAACACCTCCTGTGCTTAACTACAAGGATGGCAA
ACCGTACTGGATGGCTGTTGCTTTTGAATTAGCTCATCCATACGGCTACCCTTATCTCATGTCAGATTTT
AACTGGACAACCTACGATCAGGGACCACCAATGGACGAAGATCAGAACATAATCTCACCAACAATCAATA
AAGATGGCAGCTGCGGAAGTGGATGGGTTTGCCAGCATCGTTGGAGGCAGATCTACAGCATGGTTGGCTT
TAGGAACCAAGTAGGCTACGCACGGTTTACTAACTTCTGGTCTAATGGCAAGAATCAGATTGCTTTTGCT
CGTCAAGGATGCGGTTTTGTTGCCTTCAACAATGATGATGTTGATCTGAATGAAGTGCTCCAGACTACTC
TTCCAGCAGGCACTTACTGTGACGTTTCCACAGGATACGCCAAAGCAGGTGAATGCACTGGAAGGACTGT
CGTTGTGGGAGATGATGGAACAGCCACCATTGAAATTTCTTCATCAGATGATATTGGATTCATTGCTATC
CATATTGGGCAGATGTTGTAA
>LLOTMP004839-Intron 1: PUTATIVE_protein_coding
GTGTAAGTTGAATCAGTTTAAAATGAAATCCAGGATCAAATCTTCTTTCCTGATTTCTTTTTTTTTTAG
>LLOTMP004839-Iintron 2: PUTATIVE_protein_coding
GTATAAACCTTCGACTTTTAAGTCTGATCGTTTTGACAGAATTTCTTTAAACTTTTTTACAG

>LLOTMP004841/ LlAamyA4
>LLOTMP004841-E1 exon: PUTATIVE_protein_coding
TGGGGAATAAAATGCGGATAGTTGTGTTGATTTTGTGCTTGGTTGCCCTGGGAAGTGGTCAGCATTGCCA
AACTTCTGGGATGGCCGAACGGGTATTGTGCATTTGTTTGAGTGGAAATTCCGTGATGTGGCTGATGAGT
GTGAACGCTTCCTGGCACCCAAAGGCTATGCCGGTGTTCAGTTGTCGCCAGTCAATGAGAACGTAATCAT
TCCCAATCGTCCATGGTGGGAACGCTACCAACCAATTTCCTACGTCCTCACAACACGATCTGGAAATGAG
CAGGATTTCTTAGATATGTCCAGAAGGTGCAATGCGGTGGGAATTCGAATTTACGTTGATGTTCTCCTGA
ATCACATGTCTGCAGATAATTATCCATCAGATGGAACTGCTGGTAACTGGGCAGATACATCACAACGTTG
GTGGCCAGCTGTTCCATATGGTCCCAATGACTTCAATCCTCGCTGCGAAATTCACAACTGGGACGATCCA
TGGGAGATCAGGCAATGCGAACTAGTTGGTCTGCATGATCTCAATCAGGGCACTGAACACGTACGTAGTA
TGTTGGTGAACTTCCTGAATCACCTTGTCGAACTGGGCGTTGCTGGTTTCCGCGTTGATGCTGCAAAGCA
CGTCTGGCCTCATGATCTAGAAGTAATCTACAATCGCGTCAATAATCTGAATACTAACTTCGGTTTTGCT
CCTGGAAGTCGCCCTTACATCTACCAGGAAGTTATTGATCTAGGTGGAGGTGGAATTCGGTATGAATACA
CACCTCTAGGAGCTATAACAGAGTTCAAGTTCTCCGCCGAAATTGGAGGAGCCTTCAGACGTTACAACGA
TCTGCGCTGGTTGCGGAACTTCGGTCCTGAATGGGGCTTTGTTCCATCTGATGTTGCTCTGGTATTTGTT
GACAATCACGACAACCAGCGTGGACATGGAGCAGGTGGTGAAGCCGTTCTGACCTACAAACAGCCTAGAC
AATACAAGATGGCAACAGCCTTCACAATGGCCTGGAACTACGGAACAATTCGTCTAATGTCGTCATTTGC

CTTCGATCATGGAGATCAGGGACCACCTCAGGATGCCAATGAGAACATCATTTCCCCATCAATCAATCCT
GACAACACTTGTGGCAATGGATGGGTATGCGAACATCGCTGGAGGCAGATCTACAACATGATTGGTTTTG
CTAACCAAGTCAAGGGAACATCAGTTAACGACTGGTGGGACAACGGCCATAACCAGATAGCCTTCTGCCG
AGGAGATCAAG
>LLOTMP004841-E2 exon: PUTATIVE_protein_coding
GCTTCATTGCTTTCAACAATGAGGATCACAACTTCAGTCATACACTGCAAACCTGCTTACCAGCTGGAAC
GTACTGCGACGTCATTTCCGGTGAAAATACCGGAAATGGTTGCTCAGGCGTCACAATCACAGTAAACTGG
GATCAAACAGCTCACATTGTCATCCCTGTTGATCACTACGACGGTGTTATTGCCATTCATCGTGGTCCAA
CATCGAGGATCGGTTAAACAACAGCGTCAGTAAACTTCCCAGTCAATTTCGTGATACATATGACGGAATT
TACTCATCCAAAAGTCTGCGCAGTCTTTTGAGCAGAAGATCGTACTGAATACTACTCCACTTGGATTCTG
AAGTCTGAAAATTAAAGAAAATGATGAAATAATTTAAAAGAAA
>LLOTMP004841-Intron 1: PUTATIVE_protein_coding
GTATGAAAATGGCTTGAAAATTGGCTAAACTCTGACTTGTAACTTTAATGAACTTTAATCCTAAACTTAT
TTACAG

>LLOTMP004880_1/LlAamyB1
>LLOTMP004880_1-E1 exon: PUTATIVE_protein_coding
CGTATTTACCCAAAATTATCATTAAAAGTGAATGACCAAAGAATTTTTTGGCACCTTTCCTCGGAGTATA
AAAGACGGTAATCACGTTTCATTTCTGCAGTATGACTTTTTGATTCCATTAAATCAGTCATGAAAGTTTT
AGTGATATTCTTAGCCCTTGTGGCCTTAGCTAAGGGTCAGTTTAATCCTTACTACGAGGATGGTCGAACT
GTGATGGCTTTCCTTATGGACTGGAAGTACTCAGACATTGCTCTGGAGTGTGAGAGATTCCTTGGACCGA
AGGGGTACGCCGGTGTCCAGGTTTCATCCCCATCTGAGAATGCAATTGTAACAGATCCTCTGCGTCCTTG
GTGGGAACGCTATCATGTTGTATCGTACATCATTGGATCCAGATCAGGGACTCAGGATGATTTTGCCGAC
ATGAGTGCAAGGTGCAACAAATGCGGAGTTCGTGTTTACGTCTTTACCATCTTCAATAATATGGGAGCGT
TTCCAGATATGGT
>LLOTMP004880_1-RA-E2 exon: PUTATIVE_protein_coding
TGGAACTGCTGGATCTACAGCAGATGGACCATCTAAATCCTACCCAGCTGTACCTTATGACAGTACAAAC
TTCCATCCATCCTGTCCCATTCAATCTTACCAGCATGCCTTCATTGTACGTAACTGCGAAAATGAAGGTA
CTCCTGATCTCGATCAATCCCAAGACTACGTCAGGGAACAAATTGTTGCCTATCTCAACAATTTGATAAG
CCTCGGAGCTGCTGGCTTTCTCATTGATTCAGCCAAGAATATGTGGCCAACAGATCTGAAAGCTATCTAC
AGTAGCCTCTCGAATTTGAATGAAGACTACGGATTTTCTCCCAACTGCCGGCCATTCATCTACCAAGATG
TCATTGAGTTAGGCAAGGAAGCTGTTAACAA
>LLOTMP004880_1-E3 exon: PUTATIVE_protein_coding
ATATGAGTACCTTCCCTTAGGTGATGTGACTGATTACAAAGTTTCATATAGCCTTAATAGTATCTTCAAC
GGCAATAAATCGCTCAGTGTTTTAGCCACTTGGGGAGAATCTCTCTGGAACTTTATTCCATCGAAAAGTG
CTGTAGTTTTCGTAGAAAATGAACAAAATGAACGCGAAGGATTCTACTTTGGACAAGTCCTTAACTACAA
AGATGGAAAACCCTACATAATGGCTATTGCTTTCCTCTTGGCTCATCCATACGGTAATCCACGCATCATG
TCAGACTTTGACTGGTCAGACTCCGATCAAGGACCTCCAATGGATGATAATCAGAACATCATCTCACCGA
CTATCAATAAAGATGGTAGTTGCGGTGATGGATGGGTTTGTCAGCATAGATGGCCTCAGGTTTGCAGCAT
GGTTACATTCAGGAATCAAGTGGGTAATTCCAATTTCTGCAACTTCTGGTCCAACGGCAACAATCAGATC
GCCTTTGCGCGTGAAGGACATGGCTTCATTGTCTTCAACAATAACGACGGGAAACTCTGGCAGACTCTGC
CGACAACCCTTCCGGCTGGGACCTACTGCGACGTCATCACAGGTTATGTTAAAAATAATGAATGCACTGG
AAACAGCATTGAGGTAGCAGACGATGGAACAGCAGAATTCATCATCTACTCTGATGATCCAAATGGCGTG
ATCGCCATTCACATTGGACAAATGTTGGAATGA
>LLOTMP004880_1-Intron 1:PUTATIVE_protein_coding
GTAAGTTGAACAGCTTTAAAAGAGTCCTTGTTTTCCTGAAACAGTCTTCATGGTTTTTTCAAACTTACAG
>LLOTMP004880_1-Intron 2: PUTATIVE_protein_coding
GTAATTAAGAGTAGTTCTTTTGAAAACAATGAGGGAAAACTAAAATGCCTTCTCTTTCAG

>LLOTMP004880_2/LlAamyB2
>LLOTMP004880_2-E1 exon: PUTATIVE_protein_coding
ATGAGAAGTGCGTTAATTTTCCTTGCCTTTGTGGTCTTAGCTCGGGGACAGCATAACCCAAATTTCGTTC
CTGGACACACTGGTATAGTTCATCTATTCGAGTGGAAATGGGTGGATGTTGCTGCTGAATGTGAAAGATT
TCTCGGACCTATGGGCTTTGGAGGAGTTCAAGTATCCTCTCCAGCTGAAAATGCCATTGTGAGGGATCCT
CTGCGTCCTTGGTGGGAACGCTATCACATCATTTCGTACAAACTTGTAACGCGATCTGGGAATGAGGAAG
AATTTGCCGATATGTGTACAAGATGCAATAAAGTCGGAGTTCGTATCTACGTTGATACGGTTATCAACCA
CATGGGTTCTCTTCCGAATATGAT
>LLOTMP004880_2-E2 exon: PUTATIVE_protein_coding
TGGAACTGGAGGATCAACAGCAGATGGACCTAGCAAGTACTACCCAGCTGTACCCTACAACGTTGATCAT
TTTCATACATCTTGCCCAATAGAGAACTGGCAAGATGAATATGAAATTCGTTATTGTGAATTATTCGGTG
CTCCTGACTTGAATCAGGGAATGGGCTATGTCAGAACTACAATTGCGGAGTACATGAATCACCTGATACG

TCTCGGAGTAGCTGGTTTCCGTATTGATGCAGCTAAGCACATTAGTTCTTCTCATTTATATTTACAACT
ATTGATTTAACATACTTAAACGACTTAAATCCAGATCACGGATTTCCCGCCAGAACAAAACCTTTCATCT
ACCAAGAAGTCATAGACTTTGGCTGGGATGCTGTTTCAAA
>LLOTMP004880_2-E3 exon: PUTATIVE_protein_coding
GTACGATTACACCTACATAGGTACGGTAACGGAATTTATGTATTCATACTACATCAGCCGAGCCTTCCGT
GGTAACGATTTACTGAGGAATTTGGCAACTTGGGACCAACTTGGGGTTTCCTAAACTCCTCATATGCTC
TTGTTTTTGTTGAGAATCACGACAATGAACGCGGTGATGGAGCTGGAGGGCAGGAGATTCTCTCCTACAA
GGATGGCAAGCAATACCGTATGGCCGTTGCTTTTGCCCTAGCTCACACCTATGGTATCCCACGCATCATG
TCCTCTTTCGCCTTCACTGAGAGAGATATTGGCCCACCAATGGATTCAAATGAGAACATTATCTCACCCA
GTATTTACCCGGATGGAAGTTGCGGAAACGGTTGGGTGTGTCAGCACAGATGGAGGCAGGTAGTCAATAT
GATTGCCTTCAGGAATATTTCGGGGAATGAAAATATCACAAATTGGTGGACCAATGACAAAAATCAGATT
GCCTTTGCGAGGCACCAGCGTGGCTTCATTGCTTTCAATAACGACGCAATGAATATGAATGTGAGCCTGC
AAACGTCCCTACCGGCTGGAATCTACTGCGATGTTATATCAGGGAATTTAGTGGATAAACTCTGCACAGG
GAAGAACATAACCGTGAGAGGGGATGGAGTTGCCAGGATTCTTATAATGAAAGATGAAGAAGATCAAGTT
GTCGCTTTTCATGTTGGTCAATGA
>LLOTMP004880_2-Intron 1: PUTATIVE_protein_coding
GTAGGAGAGTTTCTTAATAGTTTTAATATAAGAGCTGCTAAGAGAGTATTTAATTTTGTTTATTTCTAG
>LLOTMP004880_2-Intron 2: PUTATIVE_protein_coding
GTACGTTGATAGTGTATATAAAATCAGAATTAGGATATGGCCCTTAGAGCCTATCTGTCAGTAAACTTTT
AGTACTGATCCTAGAAAAGATCCAAGACATGGTCTGAGACAGATCTCACAACGGCTTTTCTTTAAATTTC
AG

>LLOTMP004880_3/ LlAamyB3
>LLOTMP004880_3-E1 exon: PUTATIVE_protein_coding
ATGCATTTTATCAGTCTCGGTGGCGGAATTCTTTTGGTGCTGGTGCTTCATGTTGCGGATGGTCAATTTG
ATCCACACTTCCTCCCTGGAAGAAGTGTTATTGTGCATCTGTTTGAGTGGAAATTCTCCGATATAGCCGC
GGAGTGTGAGAATTATTTGGGCCCAAATGGATTTGGGGGCGTCCAGGTGTCCCCGATAAATGAATGTCTT
GTATCACCTGAGAGAGCGTGGTGGGAACGGTATCAACCCGTGTCGTATGCTATTGTAAGTCGATCAGGGG
ATGAAAAAGAATTTGCGGAGATGGTTAAACGATGCTACGAGGCTGGGGTACGTGTTTACGTTGACGTCAT
CTTCAATCACATGGCATCTGGGGAGGGTGAAGTTGTTGGAACTGGAGGAAGCTTGGTCTACCCAGAAGAG
CGTCTCTACCCGCACGTACCCTACGGTCCAGAAGACTTCAACCCCCATTGCGTCATTGAGGACTATCAGG
ATGTAGATCAGGTGCGAAATTGCGCTCTTGTCTCCCTTCCAGATCTCAATCAGAAGTCCGATAATGTAAA
GAGGAGTGTCATTGAATTCCTGGATCGTCTCATTGATCACGGTGTTGCGGGATTTCGGGCAGATGCATGC
AAGCACATGTGGCCGGAGGATATTAAATTCCTCTTTGGCAGTACGAAGAATCTAAGTCCTGAATTTGGAT
TCCCCGACAAGGCTAGACCATTTCTCTACCAGGAAGTTATTGACCTGGGCCAGGAACCGATATCCAA
>LLOTMP004880_3-E2 exon: PUTATIVE_protein_coding
AAACGAATACACCTCCATAGGCGTTGTTACGGAGTTCCTATTCTCAGCTGAGATTGGAAACATCTTCCGG
TACAAGAAACTCAAGGAATTCATGAAATGGGGTACAGATGAGAGATTCCTGCGATCTGATAGAGCTCTTG
TATTTGTTGAGAATCACGACAATGAGCGTGGGCATGGAGCAGGTGGAAAGGATATTCTAACGTACAAAGA
TGGCAAACGCTACCTCCTGGCCGTTCTCTTTACAATTGCCCATCCATACGGCATCCCACGCATCATGAGT
TCGTACGACTTCTCGAATAGTGACGAAGGCCCACCGGCCAATGCAAAAGGTGAAATAATCTCACCTGTTT
TCAATGACAGAGGTCTCTGTACGAATGGTTGGATCTGTCAACATCGCTGGTTAGGCGTTGCAAGTATGGT
GCAGTTCCGTAATGCTGTTGCCGGTAGTGGTATCGTCAACTGGATGGACAACGGAGAACAGCAATTTGCC
TTCTGTCGTGGAGATCTGGGGTTCGTCGCCTTCAACGGCTACACCATGTCCCATCTTAATCAACAGTTAA
GGCTGACCTACCACCTGGCATCTACTGACGACGGTAATCAGCGGAGAAGTCACACTAGATGGCTGTACTG
GGCTCGAGGTTGTTGTCAAAAATGACGGTTATGCCAACATTTTCATTCCAGGCGACTCCCCAACAGGTGT
TCTTGCCGTTCACCTGGGCTCTGCCTATATTATCGATTAA
>LLOTMP004880_3-Intron 1: PUTATIVE_protein_coding
GTATGTTCCTACGTCTTAATCTGATGTTCCTTTGTTTCCTAACTTCCTTTAAATTCTCACCCATCAATAG

>LLOTMP004881_1/ LlAamyB4
>LLOTMP004881_1-E1 exon: PUTATIVE_protein_coding
ATGAAAGCCACGATCTTTGCTTTACTTTCCCTGCTAGCCCAAACTAAGGGCTATTTTAACCCCAATTTTG
TACCAGGTCGTAGTGGGATTGTGTGGCTTTTTGAGTGGAAGTTCGCGGATATTGCTTTGGAATGTGAACG
ATTCCTCGGACCGAATGGCTTTGCAGGTGTTCAAATTTCATCCCCAGCTGAGAATGCAATCGTTACGAAC
CCCTTGAGGCCATGGTGGGAGCGCTACAACCCAGTCTCCTACATCATTGCCTCGAGATCAGGCAATGAAC
AGGATTTTAGTGATATGGTTACTCGTTGCAATAATGTCGGAGTACGGACTTATGTTGAAATCATCATGAA
TAACATGGGGGGTTATCCTAATATGGT
>LLOTMP004881_1-E2 exon: PUTATIVE_protein_coding
AGGAACTGGAGGATCAACAGCAGATGGTCCAAGCAGGAGTTTCCCAGCTGTTCCCTACACATCTGCGGAT
TTTCATGCAACATGCTCCATAACTACAATGCTCCTGCCTTTCCTAATACGCAATTGTGCCGTTGAAGGCT

TACCAGATCTTAATCAGAGCGGAGCACATACCAGAGATATGATTGTGGGTTACCTTAATCATCTAATTTC
ACTCGGCGTTGCAGGATTTCGTATAAACAGTGCAAAGTTCGTCTGGCCGTCTGACCTTAAAGTTATCTAC
GACAGGACTAATAATTTGAATACAAACTACGGCTTTGCTCCCAATAGTCGTCCGTTTATCTTCCAGGATG
TTGCAGACTATGGACTTGATGCGATTTCCAA
>LLOTMP004881_1-E3 exon: PUTATIVE_protein_coding
GTATGAATACTCCTCAATGGCGGTTGTTTCGGAGTACAAGTTTCTCCTCAATCTTGGTCGAATTTTCCGC
CATCTGGATGTTTTGAGTGTCCTAACTAACTGGGTGCAGACTTTTGGACTTCTTCCATCAGATGCGGCTT
TAGTCTTCGTTGACAGTCCCGATAGCGAGCGTGGGAGTCAAGGAAGTGAAATCCTAACCTACAAAGATGG
CAAGGCGTACATTTTGGCTCTAATCTACATGTTAGCCGTCCCCTATGGCCATCCGATAATTATGTCTTCC
TACGAATTTAAGGATGTGAATTCTGGTCCACCAATGGATGCAAACCAAAATATTGTCTCGCCTACAATAA
ATCCCGATGGAAGCTGCGGGAGTGGTTATGTGTGTCAGCACAGATGGACACCGGTGTCCAGTATGGTGGC
TTTCAGTAACAATGTTGGCGATGCTCCAGTTACAAATGTGTGGACAAATGGTCAAAATCAATTGGCTTTC
GCTCGGGATGGAGTTGGTTTTGTTGCTATTAACAGCAACGCAGAGACTTTGGATGTAAGTGTACAGACTG
GTCTTGTAGGTGGAGTATACTGCAACGTCCTCGGAGGACCTATAGTTGATGGAACTTGCGCT
GGACAGACTGTTGTTGTTGGAGATGATGGAATTGCTGCAATTACCATAACAACACAAGACAACCTCGGAG
CTATTGTACTTGATGTTAATCAACAACTACTTAATGGTGAAGCAAAGAAATTTAAACAAGAACATCCGTC
CTTGTATTAA
>LLOTMP004881_1-Intron 1: PUTATIVE_protein_coding
GTAAGTAAGAACCAAGATATTATGTCAGAAGGATACAAAGAAGGCTTCTTTACAG
>LLOTMP004881_1-Intron 2: PUTATIVE_protein_coding
GTAATATTCAGACCAGCTTTTTCAGAAAAATAGAAAAGTCTAGTATTTTATATTTCTAG

>LLOTMP004881_2/ LlAamyB5
>LLOTMP004881_2-E1 exon: PUTATIVE_protein_coding
ATGAAGTTCTTCATGATACTCTTGGCCCTTGTGGCCTCCACTAGGGGCCAGTTTAATCCGTACTTTTGGC
CTAACCACAATACTATTGTCTACCTCTTGGATTGGAAGTACTCAGACGTTGCTCTGGAATGCGAGAACTT
CCTTGGACCGAAAGGCTTTGGTGGAGTTCAGGTATCGTCTCCTTCAGAAAATGCCATTATTACGGATCCC
TACAATCGTCCATGGTGGGAACGCTTCCATGTTTTGTCCTACATAATCGGCTCGAGATCTGGCAATCAGG
ATGACTTTGCAGACATGACTGCTAGATGCAACAAGTGCGGAGTTCGCGTTTTTGTAGATGCTATCTTTAA
CGAAATGGGTGGTTTACCAGATATGGT
>LLOTMP004881_2-E2 exon: PUTATIVE_protein_coding
TGGAACAGCTGGATCTACTGGAGACGGCCCATCAAGGACTTTCCCTGCTGTTCCCTATGACAGCTCAAAC
TTTCATCCACCTTGCCCTATTCAATCCTGGCTACATGCCTTCATCGTTCGAAATTG
>LLOTMP004881_2-E3 exon: PUTATIVE_protein_coding
XXXXXXXXXXXXXXXXXXXXXXXXXXXXXXXXXXXXXXXXXXXXXXXXXXXXXXXXXXXXXXXXXXXXXX
XXXXXXXXXXXXXXXXXXXXXXXXXXXXXXXXXXXXXXXXXXXXXXXXXXXXXXXXXXXXXXXXXXXXXX
XXXXXXXXXXXXXXXXXXXXXXXXXXXXXXXXXXXXXXXXXXXXXXXXXXXXXXXXXXXXXXXXXXXXXX
XXXXXXXXXXXXXXXXXXXXXXXXXXXXXXXXXXXXXXXXXXXXXXXXXXXXXXXXXXXXXXXXXXXXXX
XXXXXXXXXXXXXXXXXXXXXXXXXXXXXXXXXXXXXXXXXXXXXXXXXXXXXXXXXXXXXXXXXXXXXX
XXXXXXXXXXXXXXXXXXXXXXXXXXXXXXXXXXXXXXXXXXXXXXXXXXXXXXXXXXXXXXXXXXXXXX
XXXXXXXXXXXXXXXXXXXXXXXXXXXXXXXXXXXXXXXXXXXXXXXXXXXXXXXXXXXXXXXXXXXXXX
XXXXXXXXXXXXXXXXXXXXXXXXXXXXXXXXXXXXXXXXXXXXXXXXXXXXXXXXXXXXXXXXXXXXXX
XXXXXXXXXXXXXXXXXXXXXXXXXXXXXXXXXXXXXXXXXXXXXXXXXXXXXXXXXXXXX
>LLOTMP004881_2-E4 exon: PUTATIVE_protein_coding
ACAAGAAGGGAACCGTGTGACAGTCCTTATGTATGCCAACATAGATGGCCTCAGGTTTGCAGTATGGTTA
CATTCAGGAATCAAGTTGGTGACGCCAATTTCTGTAACTTCTGGTCCAACGGCAACAATCAGATCGCCTT
TGCGCGTGAAGGACAAGGCTTCATTGTCTTTAACAATAACGACAAGAAGCTGTGGCAGACTCTGCAGACA
ACCCTTCCTGAGGGAACCTACTGCGACGTCATCACAGGTTATGTTAAAAATGATGAATGCACTGGAAGGA
GCATTGAGGTTGCAGACGATGGGACAGCTGAATTCATCATCTACACTGATGATCCAAATGGG>LLOTMP0
04881_2-Intron 1: PUTATIVE_protein_coding
GTAAGTAAAGGAGATTCTTATGTAAGACTCTTTAGGTGCCTTTTGTGATCAGAACGATCGATAGCTCCAA
AAACCGCTTATATTATACTTGACAG
>LLOTMP004881_2-Intron 2: PUTATIVE_protein_coding
XXXXXXXXXXXXXXXXXXXXXXXXXXXXXXXXXXXXXXXXXXXXXXXXXXXXXXXXXXXXXXXXXXXXXX
XXXXXXXXXXXXXXXXXXXXXXXXXXXXXX
>LLOTMP004881_2-Intron 3: PUTATIVE_protein_coding
XXXXXXXXXXXXXXXXXXXXXXXXXXXXXXXXXXXXXXXXXXXXXXXXXXXXXXXXXXXXXXXXXXXXXX
XXXXXXXXXXXXXXXXXXXXXXXXXXXXXX

>LLOTMP004881_3/ LlAamyB6
>LLOTMP004881_3-E1 exon: PUTATIVE_protein_coding

ATGAAGCTCTTCGTGGTATTCTTGGCCCTTGTAGCCTTCACCAGGGGCCAGTTTAATCCGTACTTTTGGC
CTGGACACAATACTATTGTTTACCTCCTGGACTGGAAGTACTCAGACGTTGCTCTGGAATGTGAGAACTT
CCTTGGACCGAAAGGCTTTGGTGGAGTTCAAGTATCGTCCCCTTCAGAAAATGCCATTATTACTGATCCC
TACAATCGTCCATGGTGGGAACGCTTCCATGTTTTATCCTACATAATCGGCTCGAGATCTGGCAATCAAG
ATGATTTTGCAGACATGACTGCTAGATGCAACAAATGCGGAGTTCGCGTTTTTGTAGATGCTGTCTTCAA
CAATATGGGTGGTTTACCAGACATGGT
>LLOTMP004881_3-E2 exon: PUTATIVE_protein_coding
TGGAACAGCTGGATCTACTGGAGACGGCCCATCGATGACTTTCCCTGCTGTTCCCTATGACAGCTCTAAC
TTTCATCCACCCTGCCCTATTCAATCCTGGCTACATGGTTTTATTATTCGCAATTGCGAAGATCCCGATT
TCTTGCCTGTTCCTGATCTCGATCAATCCCAAGACTACGTCAGGGAACAAATTGTTACCTATCTCAACAA
TTTGATAAGCCTTGGAGCAGCTGGTTTCCGTATTTTTGCAGCTAAGCACATGTGGCCAACGGATTTGGAA
GCTATCTATAGTAGTCTTTCGAATCTGAATGAGGACTACGGATTTTCTCCAAACTGCCGACCATACATCT
ACCAAGATGTCATTGATTTGGGCGGTGAAGCCGTTAACAA
>LLOTMP004881_3-E3 exon: PUTATIVE_protein_coding
GTATGAATATATCCCCCTAGGGCTTGTGTCTGAATACAATGCGTCACAGAGTCTTGCAGATATCTTCCGT
GGCAATCGACCGTTGAGTGTTTTAGCCACTTGGGGAGAGGCTGAATGGAATTTTGTTCCATCCGCAAATG
CAATTGTTTTCGTGGAAAACGAACAAAATGAGCTTGACACATGGTACTTTGGAAGAGTCCTCAACTACAA
AGATGGTAAACCCTACATAATGGCTATTGCTTTCCTCTTGGCTCACCCATTTGGCAATCCACTCCTTAAT
TCGGACTACGAGTGGACTGATCCCAACGAAGGTCCCCCAATGGATGATGATCAGAACATTCTTTCGCCGG
TTATTAACAAGAAGGGAACCTGTGACAGTCCTTATGTATGCCAACATAGATGGCCTCAGGTTTGCAGTAT
GGTTACATTCAGGAATCAAGTTGGTGACGCCAATTTCTGTAACTTCTGGTCCAACGGCAACAATCAGATC
GCCTTTGCGCGTGAAGGACAAGGCTTCATTGTCTTTAACAATAACGACAAGAAGCTCTGGCAGACTTTGC
AGACAACCCTTCCTGAAGGAACCTACTGTGACGTCATCACAGGTTATGTTAAAAATGATGAATGCACTGG
AAGAAGCATTGAGGTTGCAGACGATGGAACAGCTGAATTCATCATCTACACTGATGATCCAAATGGGGTT
ATTGCTATTCATACTGGACAGATGTT
>LLOTMP004881_3-Intron 1: PUTATIVE_protein_coding
GTAAGTACAGATCAATTGATTCCTGTGTAAAATACTGGCGTTGTATTCCGTGATCAGGATGTTCCTCCTC
GTTATGTAGAGTCTAGGAAAGGGTGCTAACTAAGTGTTTTAGACTGTAGGTGTATATATTGTGTATTTTC
TATGTGCCCTCCAATATTTAGGTTAGTTTTTCATTAAGTCTTGTCAGAAGTGAATAGGTTTGTTAAGTGC
CCAAGGCTGTAAAGGGGTCTGAACTTATTTTCTGTTAAATTATCAATTTTATACTTGACAG
>LLOTMP004881_3-Intron 2: PUTATIVE_protein_coding
GTAAGCTAACAAGCATATCTGAATAGACTTTCAAACAAAGCTAAAATACTCCTACTTGTAG

>LLOTMP004882/ LlAamyB7
>LLOTMP004882-E1 exon: PUTATIVE_protein_coding
AATTCTCATAAGTCTTTAGTATATTTCTGAGTTTTGATCGTTGAATTTCATTTGAAATGAAAATCTTTTG
GATTTTCTTCTTGGCCACAGTGGCCACTGTTCGTGGTCAATTTGATCCGCACTACGTATCAGGTCGTAGT
GTCATGGTCTTCCTTATGGACTGGAAGTATGAAGACGTTGCTCTGGAGTGTGAGAGATTCCTCGGACCTA
TGGGCTTTGCTGGTGTTCAGGTTTCAGCTGCTTCTGAGAATGATATTGTTACAAGTCCACTTCGTCCTTG
GTGGGAACGCTATCAAGTTGTCTCATACAAGATCGGTTCGAGATCTGGAGATGCAGACAGTTTTGCAGAC
ATGTGCACCAGATGCAATAACGTTGGGGTACGCGTCTACGTTGATGCCATCTTCAACAACATGGGGGGTA
TGGGGATTATGCA
>LLOTMP004882-E2 exon: PUTATIVE_protein_coding
TGGAACAGCCGGATCATTGGGAGATTCAATAACAAGAACCTTCCCTGCTGTCCCCTACACTATTCTAAAT
TTCCATAATCCACCATGTTCGATAACAGACTACAACAACACGCAACAGGTTCGCAATTGCGATCTTGTAG
GTGCTCCGGATCTTGATCAATCTCAACCCTATGTGCGAGATAAAATTGTAGGTTATCTGAATCACCTAAC
TTCCCTGGGTTGCGCTGGTTTTCGCATCGATTCAGCCAAGCATATGTGGCCAACGGATCTCCAGGCCATC
TATAGCAGTGTCATAGACTAAATACAGCCCAAGGATTTCCAGCTGGCAGCAGATCATTCATCTACCAAGA
AGTCATTGATTCTGGCCATGAAGCAATTAAGAAGTATACXXXXXXXXXXXXXXXXXXXXXXXXXXXXXXX
XXXXXXXXXXXXXXXXXXXGAT
>LLOTMP004882-E3 exon: PUTATIVE_protein_coding
GCAATGAACCTCTGAGCACCTTGAGTACTTGGGGTCAATCAAACTGGGGATTCCTTCCATCGGATCGTGC
TTTGGTGTTTGTTGAGAATCACGACAACGAACGTGGTCAATACGAATCAGCAGCGCTCAACTATAAAGAT
GGCAAACCCTACAAAATGGCTGTGGCCTTCGCTTTAGCACATCCATTTGGAAATTTACGCATCATGTCCG
ATTTTGACTGGACAACCTACGATCAGGGACCACCAATGGATGCCAATCAGGATATTATTTCACCCACTAT
CAATGCAGATGGTAGCTGTGGAAGTGGATGGGTATGTCAGCATCGTTGGAGGCAGATTTACGGTATGGTT
GGCTTCAGGAATACCGCAGGTACAACAGAAGTCACCAACTTCTGGTCCAACGGCAACAATCAGATTGGTT
TTGCTCGTCAAGGACGTGCCTTCGTGGCTTTCAACAACAACGACGCGGATATGAATCAGACTCTTCAGAC
AACCCTTCCAGCAGGCACGTACTGTGATGTCATAACCGGCGTTGCGGAGAATGGAGCCTGCACAGGAAGA
ACCATTGTTGTAGCAAATGATGGAACGGCTAACTTTGTTATCCTTACATCAGATGCAGATGGTGTGATTG
CCATTCATGTTGACCAGAAATTGTAA

>LLOTMP004882-Intron 1: PUTATIVE_protein_coding
GTAAGTGCGGGTATACGGACAGATGGTCTGGAGTGTCTGGGCTGTTTTGGCAATCTTGTCTTGTTATCAT
ATTAACTAAATTTCTGTTACAG
>LLOTMP004882-Intron 2: PUTATIVE_protein_coding
GTTACTGAATTCCTCTATTCGTACTACCTTACTGATATCTTCAAAG

>LLOJ004885/ LlAamyB8
>LLOJ004885-E1 exon: Putative_protein_coding
ATGAAGCTCTTCGTGGTATTCTTGGCCCTTGTGGCCTTCACTAGAGGACAATTTAATCCGTACTTTTGGC
CTAACCACAATACTATTGTCTACCTTTTGGACTGGAAGTACTCAGACGTTGCTCTGGAATGCGAGAACTT
CCTTGGACCGAAAGGCTTTGGTGGAGTTCAAGTATCGTCCCCTTCAGAAAATGCCATTATTACGGATCCC
TACAATCGTCCATGGTGGGAACGCTTCCATGTTTTGTCCTACATAATCGGCTCGAGATCTGGCAATCAAG
ATGATTTTGCAGACATGACTGCTAGATGCAACAAATGCGGAGTTCGCGTTTTTGTAGATGCTATCTTTAA
CGAAATGGGTGGTTTACCAGATATGGT
>LLOJ004885-E2 exon:Putative_protein_coding
TGGAACAGCTGGATCTACTGGAGACGGCCCATCAAGGACTTTCCCTGCTGTTCCCTATGACAGCTCAAAC
TTTCATCCACCTTGCCCTATTCAATCCTGGCTACATGCCTTCATCGTTAGAAATTGCGAAAACGCAGATT
ACTTGCCTGTACCTGATCTGGATCAATCTCAAGACTACGTCAGGGAACAAATTGTTACTTATCTAAATAA
TTTGATAAGTCTCGGAGCTGCTGGTTTCCGTATTCTTCAT
>LLOJ004885-E3 exon: Putative_protein_coding
XXXXXXXXXXXXXXXXXXXXXXXXXXXXXXXXXXXXXXXXXXXXXXXXXXXXXXXXXXXXXXXXXXXXXX
XXXXXXXXXXXXXXXXXXXXXXXXXXXXXXXXXXXXXXXXXXXXXXXXXXXXXXXXXXXXXXXXXXXXXX
XXXXXXXXXXXXXXXXXXXXXXXXXXXXXXXXXXXXXXXXXXXXXXXXXXXXXXXXXXXXXXXXXXXXXX
XXXXXXXXXXXXXXXXXXXXXXXXXXXXXXXXXXXXXXXXXXXXXXXXXXXXXXXXXXXXXXXXXXXXXX
XXXXXXXXXXXXXXXXXXXXXXXXXXXXXXXXXXXXXXXXXXXXXXXXXXXXXXXXXXXXXXXXXXXXXX
XXXXXXXXXXXXXXXXXXXXXXXXXXXXXXXXXXXXXXXXXXXXXXXXXXXXXXXXXXXXXXXXXXXXXX
XXXXXXXXXXXXXXXXXXXXXXXXXXXXXXXXXXXXXXXXXXXXXXXXXXXXXXXXXXXXXXXXXXXXXX
XXXXXXXXXXXXXXXXXXXXXXXXXXXXXXXXXXXXXXXXXXXXXXXXXXXXXXXXXXXXXXXXXXXXXX
XXXXXXXXXXXXXXXXXXXXXXXXXXXXXXXXXXXXXXXXXXXXXXXXXXXXXXXXXXXXXXXXXXXXXX
XXXXXXXXXXXXXXXXXXXXXXXXXXXXXXXXXXXXXXXXXXXXXXXXXXXXXXXXXXXXXXXXXXXXXX
XXXXXXXXXXXXXXXXXXXXXXXXXXXXXXXXXXXXXXXXXXXXXXXXXXXXXXXXXXXXXXXXXXXXXX
XXXXXXXXXXXXXXXXXXXXXXXXXXXXXXXXXXXXXXXXXXXXXXXXXXXXXXXXXXXXXXXXXXXXXX
XXXXXXXXXXXXXXXXXXXX
>LLOJ004885-Intron 1:Putative_protein_coding
GTAAGTATATAAGGAGTATATTCTTATGCAAGACACTTTAGGTGCCTTTTGTGATCAGAACGTTCGATAG
CTCCAGAAACCGCTTGTATTATACTTGACAG
>LLOJ004885-Intron 2:Putative_protein_coding
XXXXXXXXXXXXXXXXXXXXXXXXXXXXXXXXXXXXXXXXXXXXXXXXXXXXXXXXXXXXXXXXXXXXXX
XXXXXXXXXXXXXXXXXXXXXXXXXXXXXXX

>LLOTMP005909_1/ LlAamyC1
>LLOTMP005909_1-E1 exon:PUTATIVE_protein_coding
ATGCATTTTATCAGCTTCGGTGGTGGAATTCTTTTGGTGCTGGTGCTTCATGTTGCGGATGGTCAATTTG
ATCCACACTTCCTCCCTGGAAGAAGTGTTATTGTGCATCTGTTTGAGTGGAAATTCTCCGATATAGCCGC
GGAGTGTGAGAATTATTTGGGCCCAAATGGTTTTGGGGGCGTTCAGGTGTCCCCGATAAATGAATGTCTT
GTTTCCCCTGAACGAGCATGGTGGGAACGGTATCAACCCGTATCGTATGCTATTGTAAGTCGATCAGGGG
ATGAAAAAGAATTTGCAGAGATGGTTAAACGTTGCTACGAGGCTGGGGTACGTGTTTACGTTGACGTCAT
CTTCAATCACATGGCATCTGGGGAGGGTGAAGTTGTTGGAACTGGAGGAAGCTTGGTCTACCCAGAGGAG
CGTCTCTACCCGCACGTACCCTACGGTCCAGAAGACTTCAACCCCCATTGCGTCATCGAGGACTATCAGG
ATGTAGATCAGGTGCGAAATTGCGCTCTTGTCTCCCTTCCAGATCTCAATCAGAAGTCCGATAATGTAAA
GAGGAGTGTCATTGAATTTCTGGATCGTCTCATTGATCACGGTGTTGCGGGATTTCGGGCAGATGCATGC
AAGCACATGTGGCCGGAGGATATTXXXXXXXXXXXXXXXXXXXXXXXXXXXXXXXXXXXXXXXXXXXXXX
XXXXXXXXXXXXXXXXXXXXXXXXXXXXXXXXXXXXXXXXXXXXXXXXXXXXXXXXXXXXXXXXXXXX
>LLOTMP005909_1-E2 exon:PUTATIVE_protein_coding
AAACGAATACACCTCCATTGGCGTTGTTACGGAGTTCCTATTCTCTGCTGAGATCGGAAACATCTTCCGG
TACAAGAAACTCAAAGAATTCATGAAATGGGGTACAGATGAGAGATTCCTACGATCTGATAGAGCTCTTG
TATTTGTTGAGAATCATGACAATGAGCGTGGGCATGGAGCAGGTGGAAAGGATATTCTAACGTACAAAGA
TGGCAAACGCTACCTCCTGGCCGTTCTCTTTACAATTGCCCATCCATACGGCATCCCACGCATCATGAGT
TCGTACGACTTCTCGAATAGTGACGAAGGCCCACCGGCCAATGCAAAAGGTGAAATAATCTCACCTGTTT
TCAATGACAGAGGTCTCTGTACGAATGGTTGGATCTGTCAACATCGCTGGTTAGGCGTTGCAAGTATGGT
GCAGTTCCGTAATGCTGTTGCCGGTAGTGGTATCGTCAACTGGATGGACAACGGAGAACAGCAATTTGCC

TTCTGTCGTGGAGATCTGGGGTTCGTCGCCTTCAACGGCTACACCATGTCCCATCTTAATTCAACAGTTA
AGGTCTGCCTACCACCTGGCATCTACTGCGACGTAATTAGCGGGGAAGTCACACTAGATGGCTGTACTGG
GCTTGAGGTTGTTGTTAAAGATGACGGTTATGCCAACATTTTCATTCCAGGCGACTCCCCAACAG
>LLOTMP005909_1-Intron 1:PUTATIVE_protein_coding
AAXXXXXXXXXXXXXXXXXXXXXXXXXXXXXXXXXXXXXXXXXXXXXXXXXTTCCTACGTCTTTATCTT
ATGTTCCTTTGTTTCCTAACTTCCTTTAAATTCTCACCCATCAATAG

>LLOTMP005909_2/ LlAamyC2
>LLOTMP005909_2-E1 exon: PUTATIVE_protein_coding
ATGAAGTTTTTGATTGGATTTGGAGTAGTTCTGATCCTGGTAGCTGTTGCTGCGCAGTACGATCCGCATT
ATCTTGACAAGAGAACTGTAATGGTTCATTTATTTGAATGGAAATGGAACGACATTGCCAAGGAGTGTCA
GGATTATCTAGGACCAAATGGGTTTGGTGGAGTTCAG
>LLOTMP005909_2-E2 exon: PUTATIVE_protein_coding
GTTTCACCGGTTAATGAGAATTGGGTTTCAGGAGAACGTGCCTGGTTCGAACGCTATCAACCAATATCAT
ACAAACTAACCACACGATCTGGAACTGAGGACGAATTTGCCGCAATGGTAAAAACCTGCCGACAAAATGG
CGTTCGAATCTTCGTTGACATTGTGGTCAACCACATGGCTTCAGGTGCTCTTGAAGATACAATTTATGGA
ACAGGTGGATCTGAGGCACATCCTGGTCCTTTTGACTATCCAGCTGTTCCGTATGAAAAGAATGACTTTC
ATCCTGATTGTAGCATCTCAGACTACCAGAATGTGTATCAAGTTAGAAATTGTCAGTTGTCTAGCTTGAG
GGATCTCAATCAAACTATTCCCTATGTCAGAGAGAAGATCCTAGACTTCCTCAATCATTTAGTAGACTTG
GGAGTAGCGGGCTTCAGAATTGACGCAGCCAAGCATATGGATCCGAAGGACTTGCGATACATCTACAATC
ATATAAAGAAATTAAATAGGACGCTGAGTTTCAGAGCAGGAGACAAAGCATTTATTTTCCAGGAAGTTAT
TGATCTAGGAGGAGAAGCTGTATCATC
>LLOTMP005909_2-E3 exon: PUTATIVE_protein_coding
ACGTGAATATATATCGCTGGGCGTTGTAACAGAGTTCAAGGCATCCGATGATCTTGGAAAGCTTTTCCGG
GGACAAGTTGCTCTGTCGACCTTGGAAAGATGGGGTCCACAATATGGGCTTCTGCCTTCAAACCGAGCTC
TTGCCTTTGTTGAGAATCACGACAACGAACGTGGGCACGGAGCTGGCGGAACAAATATCCTAACGTACAA
GGATGGGAAAATCTACACAATGGCCGTTGTATTCAACTTAGCCCACTCCTATGGGGTTCCACGAATGATG
AGCAGCTATGAATTTAACGATCCAAGCCAGGGACCTCCGCATGATGACAATAACAACATTCTAACTCCTG
AATTCTCTGCAGATGGTAATTCCTGCACTAATGGTTGGGTTTGTCAGCATCGTTGGCGTCCTATGAGGAA
TATGGTGAAGTTCCGGAATATCGTCGGTCGGAAGCCAGTTAGGAAGTGGTATGACAACGGAAGCAATCAG
ATAGCCTTCTCCAGAGGCAATCAGGGCTTTGTCGCCTTTAACATGGACATTGTTGACTTTAACCAACAAG
TTCCAACGGATTTACCAGACGGGGTATATTGCGACGTTATTACAGGTGAGAAGAATGGCAATGAGTGTAC
TGGAAAGGTAGTTATTGTGAGCAAGAGGAAGGCTGCTGTTATCCTAAGGGCAGATGACGACTACGGAGTT
CTGGCGATTCATTCAGAGTCTAAGTTGTAAGAATTATGCTGAAATGTTTGCTCCAACAATTCAATGACTT
CGGTGCTTGGAAGATTAAGATGAAACTTTTTTGTTTGAGATAGTTTCAGCTAACCTATTTCTATTATTTT
TTTATTTTAATAAAGAAGATGACTTGCTGTAGTA
>LLOTMP005909_2-Intron 1:PUTATIVE_protein_coding
GTAAGTAAAGAGGAAACTAACTTAAAGACTATAATTCACAAGACTGACTTTACAATGAAGACCTTCATGT
TCCGCTTTCTTCATGTTCAGTAAAGGACTAATATTTCCTAACGAATCCTTATTTAGAGTCTCTGAACTCT
GAAGATCAACTCTCTCAACAATACGGAGTTTGAAGCCCTTTTTAGAGGATCTAGGCCCGTTGTTATGAGA
CAGACTGTTAAAACAAACTGCTACAACTAGGCCATCATAACATGCAACAAAGATTACAGAGAAAGCTTCT
CTTCATTCTTCCAG
>LLOTMP005909_2-Intron2:PUTATIVE_protein_coding
GTGAGTAGAAGACTAGTAGCGGTTTAAGTATTCCCGATTTTTGTTCAATTTAATATCATTTCAG

Maltase
>LLOTMP000566/ LlAglu1
>LLOTMP000566-E1 exon: PUTATIVE_protein_coding
XXXXXXXXXXXXXXXXXXXXXXXXXXXXXXXXXXXXXXXXXXXXXXXXXXXXXXXXXXXXXXXXXXXXXX
XXXXXXXXXXXXXXXXXXXXXXXXXXXXXXXXXXXXXXXXXXXXXXXXXXXXXXXXXXX
>LLOTMP000566-E2 exon:PUTATIVE_protein_coding
AATATAGGTATTACCCAGAGACTCCAATATGTAAAGGATCTAGGAATGAGCGGCACATGGTTGTCTCCTA
TCTTTAAATCGCCAATGGCTGATTTTGGCTATGACACAGCTGACTATACGGCGATTCAACCTGAGTACGG
AACTATGGAAGACTTCGAAGCACTAATTAAAAAAGCCAATGAAATTGGAATAAAGATTATCTTGGACTTT
GTACCGAATCATTCGAGTGATCAACATGAATGGTTTAAAAAGAGCGTTGATAGAGTACCAGGCTATGAGG
ATTTCTATGTGTGGCATCCTGGTAAGATTGTTAATGGACAGCGCCAGCCACCTAATAACTGGGTTTCTGT
CTTCCGCGGAAGTGCTTGGACATGGAATGAAAAGCGCCAAGAGTACTACTTCCATGCTTTCTTAAAAGAA
CAACCAGATTTGAACTACAGAAATCAAGCGACTGTGGATGCAATGAAGGAGACTCTGCGATTTTGGATGC
AAAAAGGAGTATTTGGATTTCGTATTGATGCTGTGCCATATCTTTTCGAAGTTCCTGCAGATTCAAATGG
GAACATTCCTGATGAACCCTTAACCGGGCCTAGTTGTCCAGACCCTACACATGACTGCTACACGCAGCAC
ATTTACACTCAAAATTTGGATGAAACTTTCGATATGGTATACCAATGGCGGGAAGTTGTGGATGAATTTG

CAAAGCAAACTGATAACGTACCAAGGATTCTAATGATAGAAGCTTATACGCCATTGGAAAATATGAAGAG
ACTCTATGAAGATGGACATGGACGGGAAGGTGCTCAGCTTCCCTTCAATTTTGAGCTGATCAGCAAACTT
AACGGAAAGTCAACCGCTAAGGATTTCAAAGACGTAATTGATGGATGGATTAGTCGTCTGCCTCAAGGAA
AGGAAAACAATTGGGTCCTAGGGAATCATGATAATAAGAGAATTGCATCGAGATTTGGGATTGATCGGGC
TGATCTCATAAATATACTTCTGCAGACACTTCCCGGGATGGCGGTTACCTACAAT
>LLOTMP000566-E3 exon: PUTATIVE_protein_coding
GGTGAAGAACTAGCTTTGAAGGATGTCTATATCAGTTGGGAAGATACCATAGATCCTGCTGGTTGTAATA
CAAGTCCTGATGTTTACGAGCAATATTCAAGAGACCCAGTGCGAACTCCTTTTCCCTGGAATGCCAATAA
AAATGCTGGCTTCTCTAATTCTGACAAAACTTGGCTTCCTGTCTCATCTGATTACAAGGAAGTTAATGTT
GAAGCACAGGAAAAGGCAACCCGTAGTCACCTCAAGTCCTTCCGAAAACTGACAAAGATGCATCAACATG
AAAAAGCTTTCACCCAGGGGCAACTTTTGATGAAACTTGTTGGAACGGACATCCTGGCGTACGAAAGGAG
GGTACCTGGTACTAAACCTGAGGAAAACTTTGTGATGATCTTGAATTTTAGCAACAATACTCATAAAGTC
AACATCCCACAACTTTTCCCGCAAATGTCACAAATGTATCGCATCGAAGTTGTTTCTATGCACAGCCTCA
GTCATAAGGAAGGG
>LLOTMP000566-E4 exon: PUTATIVE_protein_coding
GACACAATTGACGGGAAGAACTTTGAAGTAAAACCCAATGAAGCTTTCGTACTTCGTGGAGGAGCTCCAA
GACTTTATTCATATTTGATTTTATGTTTCACAATGTTCTCCGCATTCTTCAACATCAACTTAACCCGTGC
CTGA
>LLOTMP000566-Intron 1:PUTATIVE_protein_coding
XXXXXXXXXXXXXXXXXXXXXXXXXXXXXXXXXXXXXXXXXXXXXXXXXXXXXXXXXXXXXXXXXXXXXX
XXXXXXXXXXXXXXXXXXXXXXXXXXXXXXXXXXXXXXXXXXXXXXXXXXXXXXXXXXXXXXXXXXXXXX
XXXXXXXXXXXXXXXXXXXXXXXXXXXXXXXXXXXXXXXXXXXXXXXXXXXXXXXXXXXXXXXXXXXXXX
XXXXXXXXXXXXXXXXXXXXXXXXXXXXXXXXXXXXXXXXXXXXXXXXXXXXXXXXXXXXXXXXX
>LLOTMP000566-Intron 2:PUTATIVE_protein_coding
GTGAGTTTTCAGACCTTTATGACCTTCTGATATTCTACCATTTACAATTTCATTTTTAAAGTTAAATCAT
AGTTTAAAGCTAATTTTAATCAATTTATAATTAATTTTTCTTTTTTGTTTGCTAAACGTTACATTTTTTT
TTTGGACTTTAG
>LLOTMP000566-Intron 3:PUTATIVE_protein_coding
TAATTGAAGAATTTTATACCTTAATCCTAATTTGGAATATTCTAATTTTACTTCACTCTTTCTTTCCAGG

>LLOTMP008156/LlAglu2
>LLOTMP008156-E1 exon: PUTATIVE_protein_coding
GTTGTGGGAATATCTCTGGGGACTTCAAAGCTACACAATGAGAGTGATCATTTTTGTTTTTCTGGTGATT
TCTGCCACTACTGAGGGTGTCTGGTACAATAATGGGAATTTCTATCAAATATATCCCAGATCCTTTATGG
ACTCTAACAATGACGGTGTGGGTGATCTGAAAG
>LLOTMP008156-E2 exon: PUTATIVE_protein_coding
GTATTACCCAGAGACTCCAATATGTAAAGGATCTAGGAATGAGCGGCACATGGTTGTCTCCTATCTTTAA
ATCGCCAATGGCTGATTTTGGCTATGACACAGCTGACTATACGGCGATTCAACCTGAGTACGGAACTATG
GAAGACTTCGAAGCACTAATTAAAAAAGCCAATGAAATTGGAATAAAGATTATCTTGGACTTTGTACCGA
ATCATTCGAGTGATCAACATGAATGGTTTAAAAAGAGCGTTGATAGAGTACCAGGCTATGAGGATTTCTA
TGTGTGGCATCCTGGTAAGATTGTTAATGGACAGCGCCAGCCACCTAATAACTGGGTTTCTGTCTTCCGC
GGAAGTGCTTGGACATGGAATGAAAAGCGCCAAGAGTACTACTTCCATGCTTTCTTAAAAGAACAACCAG
ATTTGAACTACAGAAATCAAGCGACTGTGGATGCAATGAAGGAGACTCTGCGATTTTGGATGCAAAAAGG
AGTATTTGGATTTCGTATTGATGCTGTGCCATATCTTTTCGAAGTTCCTGCAGATTCAAATGGGAACATT
CCTGATGAACCCTTAACCGGGCCTAGTTGTCCAGACCCTACACATGACTGCTACACGCAGCACATTTACA
CTCAAAATTTGGATGAAACTTTCGATATGGTATACCAATGGCGGGAAGTTGTGGATGAATTTGCAAAGCA
AACTGATAACGTACCAAGGATTCTAATGATAGAAGCTTATACGCCATTGGAAAATATGAAGAGACTCTAT
GAAGATGGACATGGACGGGAAGGTGCTCAGCTTCCCTTCAATTTTGAGCTGATCAGCAAACTTAACGGAA
AGTCAACCGCTAAGGATTTCAAAGACGTAATTGATGGATGGATTAGTCGTCTGCCTCAAGGAAAGGAAAA
CAATTGGGTCCTAGGGAATCATGATAATAAGAGAATTGCATCGAGATTTGGGATTGATCGGGCTGATCTC
ATAAATATACTTCTGCAGACACTTCCCGGGATGGCGGTTACCTACAAT
>LLOTMP008156-E3 exon: PUTATIVE_protein_coding
GGTGAAGAACTAGCTTTGAAGGATGTCTATATCAGTTGGGAAGATACCATAGATCCTGCTGGTTGTAATA
CAAGTCCTGATGTTTACGAGCAATATTCAAGAGACCCAGTGCGAACTCCTTTTCCCTGGAATGCCAATAA
AAATGCTGGCTTCTCTAATTCTGACAAAACTTGGCTTCCTGTCTCATCTGATTACAAGGAAGTTAATGTT
GAAGCACAGGAAAAGGCAACCCGTAGTCACCTCAAGTCCTTCCGAAAACTGACAAAGATGCATCAACATG
AAAAAGCTTTCACCCAGGGGCAACTTTTGATGAAACTTGTTGGAACGGACATCCTGGCGTACGAAAGGAG
GGTACCTGGTACTAAACCTGAGGAAAACTTTGTGATGATCTTGAATTTTAGCAACAATACTCATAAAGTC
AACATCCCACAACTTTTCCCGCAAATGTCACAAATGTATCGCATCGAAGTTGTTTCTATGCACAGCCTCA
GTCATAAGGAAGG
>LLOTMP008156-E4 exon: PUTATIVE_protein_coding

GACACAATTGACGGGAAGAACTTTGAAGTAAAACCCAATGAAGCTTTCGTACTTCGTGGAGGAGCTCCAA
GACTTTATTCATATTTGATTTTATGTTTCACAATGTTCTCCGCATTCTTCAACATCAACTTAACCCGTGC
CTGA
>LLOTMP008156-Intron 1:PUTATIVE_protein_coding
GTAATTCAGTGCATGTGACTTTGTGTAATTTAGATGTGGTTCAGTCAATATAAAAGTCAAAAGAAGAAAT
TATTAATCATCAAGTCTCCGGTTTAATTTCGCGGGGATTTGTTGCGCTTCGAGCAAATTTAAGATACAAA
AAAATTATGATGATTTATAAGTATATATAGAAGAAAGATAGAAGATCTATGCGCTGTTATTCTATAAAAA
TTAGGCCGTTTTTTCCGAATAGTGAAAACTTTTTTTTAAGAAATAATGGGGGATTTGGGGATGGAAGTTT
TTAGTTTTTTTCATATTATTTTATGGTACTCACCATAAAATACACACCAAATTTAAATTGGATTACAACG
ACTTGTATCTTTTAAACAATATAG
>LLOTMP008156-Intron 2:PUTATIVE_protein_coding
GTGAGTTTTCAGACCTTTATGACCTTCTGATATTCTACCATTTACAATTTCATTTTTAAAGTTAAATCAT
AGTTTAAAGCTAATTTTAATCAATTTATAATTAATTTTTCTTTTTTGTTTGCTAAACGTTACATTTTTTT
TTTGGACTTTAG
>LLOTMP008156-Intron 3:PUTATIVE_protein_coding
GTAATTGAAGAATTTTATACCTTAATCCTAATTTGGAATATTCTAATTTTACTTCACTCTTTCTTTCCAG

>LLOTMP002257/ LlAglu3
>LLOTMP002257-E1 exon: PUTATIVE_protein_coding
ATGAAGCTTCTTGGACTTTTATTGGTCACTGTGTTGACGGTGGAAGGGCATTTGCTGGGTGAACGGCAGT
CTTCAGAGAAAGCCGATTTACGGCATGATCATCCAGAATTGGATTGGTATGAACGAGCAAATCTCTACCA
AATCTACCCGAGATCCTTCATGGATAGCGATGGGGATGGAATTGGAGATTTAAATGGCATCACAGCGAAA
TTGGAGCATCTTCAGGAGTCTGGGATCTTTGCAACATGGCTCAGTCCAATTTTCAGCTCCCCCATGCGAG
ATTTTGGCTATGATATTTCAAATTTTACAGAAATTAATTCCGACTATGGTACCATGGAAGACTTCCTGCA
ACTTCTGCATGAAGCACGAAGGCTCAATATTCGTCTCATCTTGGACTTTGTGCCCAATCACACGAGTGAT
CTGCATGAATGGCTTGTAAAGTCCGAGGACAATGATCCTGAATACCGAGACTACTACGTATGGAGGGATG
CACGGTATGTGGGGGAAGAGAGACACCCACCAAATAATTGGCTTTCCGTCTTCCATGGACCTGCGTGGAC
GTGGAGTGAAAAACGGCAACAGTACTACCTTCATCAGTTTGCCAAAGAGCAGCCAGATTTGAATTTTGAA
AATGAGAAAGTGGTGGAAGAGATGACGAATGTATTGAAATTTTGGCTGGATATTGGTGTCGACGGCTTCC
GTGTGGATGCCATCAATCACCTCTTTGAGGACCCCAGTTTCGCCGATAATCCACCTAATCGTGAGATTTA
TGATCCAATGGACTATAGCCACTTTGACACAATTTACAGTAAAGATCTTCCAAAGTCCTATCAGCAGGTG
TATGATTGGCGCAAATATCTCGATAGCTATGCTGAAGAGCACGGCACGGATACC
>LLOTMP002257-E2 exon: PUTATIVE_protein_coding
AGGGTCCTCCTGACTGAGGCATACACCAACATCACGAATACGATGAAGTGGCAAATGTCAGAAGATGGTA
CGCAGAAGGGTGCACATTTCTCATTTAATTTTAACTTAATTATGGAGCTCAAGACTCTCCCCGATGAGCT
CAATGCGGCAGATGTGAAGGCAACCATTGATGAGTGGCTTGAACACCTCCCAGATGGAGTTCCACCAAAT
TGGGTG
>LLOTMP002257-E3 exon: PUTATIVE_protein_coding
CTTGGCAATCACGACCGCCCACGCGTTGCATCGCGTTTTGGTGTGGATTTGGTGGACAATATGAATATTC
TCGTGCAAACCCTCCCTGGAGTTGCGGTGACATACTACGGTGAGGAGATTGGAATGGAGGATTTCCGTGG
AATCTCCTGGGAGGATACACAGGATCCTCAGGCGTGTGGTTCCAATGAAACAGTCTACCAATTGTATACG
AGGGACCCTGTACGAACCCCAATGCAGTGGGATGATACCCAAAATGCGGGCTTCTCCACAAATCCCTCCC
CGTGGCTACCTGTACATCCAGACTATGTGCAAAATAACCTGGCACAGCAGAAGGTGGCTGAAACGAGTAC
ATATAAATTGTTCCTGGATTTAGTAAAGCTCCGCATTGATCATGCCCTAGAGTATGGAGATTTTAAGTCA
GAAGCTCTAAGCAATGGAGTCTATGCCTACAGTCGGAATATGGAGGGACATGAAAGTCTCGTGGTGGCAC
TCAATTTCGCCAGTACAGAGGCAACAGTTGACCTTACTCCCGTCCTTCTTGAGAATTCACATTCAGAAGG
TCTAGTCGAATTGGCTACAACCAAGTCCATTCATAAGAGA
>LLOTMP002257-E4 exon: PUTATIVE_protein_coding
AGTGATGTCGTTAACTTGAGGACCCTCACACTGCAGCAGTACGACGGTGTCATTCTACGAGTTAACTCAT
CCGCTGCAACAATCTTCATTTCCTCATTTGCTCTTCTTCTTGTTGTACTGAAAACACTCCTTTACTAA
>LLOTMP002257-Intron 1:PUTATIVE_protein_coding
AAGTAAGCTCGTTGAACTTTCTCAAGTACTCATTTTGCTATATATTAGTTGGTATACCACATATGGGCCT
ATTTCTTACAAGTCTGATTAACGAAAGATTGAAAAAAAAAGAATATTTGTTGGATATAAATTTGGTTGAT
GAATTTTTATTAAAAAAATTTAGCAAATTAACTACAAAATTAGTCAAAAAATTATTACTGACTGATTAAT
TGTTTTTTTCTTCTTTTAGTAAGTTAAAGCTTAACATAAAACTTTTCAAATTCAATGCTCTTTCTTGGGT
ATTACTTAATTAATTTTATTTAAAATAGATTCAAATTAAAAGTCTTCTTCAGCTCTAAAACTCTCAAGTA
CTTTGGGAATTTTTAAGTCCATTCTTTGGGGCTCAAAAGTACATAAAAATTATTTTAGAAAATGACGAAT
TTCCCAACAATTTTGTGATTGTTCCAAAAAATATCAATTCTACAATCTACATCCTCTCATACAAAAGATA
AATCAAATCACAACGCATAGGAAGCTCCGCGTTGTGGTACACCAACTCACATAAGTCGTGGACATATTAT
CAGTGATTTGTTCAATTGCTTTCAATATT
>LLOTMP002257-Intron 2:PUTATIVE_protein_coding

GTATGCTCATGATTGAAATTCAAACAAAATCATTTTAATTAAATTTAAATTCAATCCCAATATCTCTCAA
ATTTTCCACATAG
>LLOTMP002257-Intron 3:PUTATIVE_protein_coding
GAGTAAGTTTTTTTAAAAAGAAAATTAATCTCATTAACTCATTGAAATAATTTTCTTTTGCTAATAATT
AACTTTTTTTCTAATTTTT

Amino acid transport protein
>LLOTMP006803/ LlCD98hc
>LLOTMP006803-E1 exon: PUTATIVE_protein_coding
ATGGTGCGCGAGAAGGTTGACATGTCTGAGGAGGGAGCCGATGAGAAGATGCTTGGCGCCGGTGAGGAGG
AACAGCAGAAACTGGCAAAGAAGGAGGAGGTAAAATTCATCAGTGGCGATCAGCAGAATGGCGATGCAAA
GATCGATATTGGAGCCATTGACAAGACATTCACGGGATTGACAAAGGAGGAACTCATGAAGTACGCCAAT
GATCCCTTCTGGATTCGCTTGCGTTGGTTCCTCTTCATTTGCTTCTGGGGCCTCTGGGCTGGGATGCTTG
TTGGAGCTGTTCTCATTATTATTTATGCACCAAAATGTGCAGCTCCTGAGCCACTGTCATGGTGGAAGCA
GGGACCACTGGTGAAGATTGATACACCCACACCAGATAATATTCAAATTGAAGGCATTAAGGGACTAGGC
GTGAAGGGTGTCATCTACGAAGTTCCCGCTGATGAGACGTACAATATTGGAAGTGATCCTGCTATTTCTG
ATGCACTGAAGAAGTTGGCGGCATCTTTCGCTGCCTATGGCATTAATTTAATTGTTGATCTCACTCCGAA
TTTCGTTTCGCGTGGAGATCCATTGTTTCAGAAAGCTCTCAATAATCCTGATGTAAGAAGTGCATTTGTT
ACACGCGAAGGACACCAAGTTCCCAATAATTGGCTCTCATTGGTCAATGGAAGTGCATGGACAAATGAAG
GGAATCTCATCTTTCTCCAGCAATTTGGGCATGATCGCTTTGATCTTCAACTCAATGATCCCGTTGCTCT
GACAAAACTAAAGGGAGTCCTCAAGGAGGTGGTTTCTCTCGGAGCTAAAGGCATTCGTCTGGCCAATACG
AAGCACTTTATTGTTGATCGAGAACTTAAGGACAATGTACCATCGAATAATCCAAACTACGATCATACCC
AGTATGGTTTCTGGACGCACGCTCATACCACATATCAAGATGGATTGGGTGATCTCCTGTATGATTTAAA
GGCGTACGTTCACAATATTACAAACTCCGAAGGTTTTCTCGCAGCATCCGATGACATTGATCGTCCGGAA
GTTTATATGTCGTCCAGAGGAGTACAATCTCTGGATATGATTCAGCTTGGACGAGTAACCAATCTTCTGG
CCAATAGTACCAGTGCTGATCATATCTACAGGGAGCTACAGAATGTGTGGAAGAGTACAGTTGAAACAGG
GAAGCATTTCTGGCTTCAATGGAACTATGTGAAGCAGGAACTTCTGCAACAAATCACAATTTCCGAATAC
AACATCTTCCTCCAGCTCATCCCTGGTGTTCCAGTGTTCAATGTAGATGCTTCCAAAGCAAAGGCGGAGG
ACTACTCTACGGAGGCATTCCACAAAATCCGCACTTCACCATCCTACATGCACGGGAGCTTTGATCTCTA
TCGAGATGTCAACAGCACCGTTATTGGCTATTCCAG
>LLOTMP006803-E2 exon: PUTATIVE_protein_coding
GACAAAGTCCGGAAATCCAGGCTTCTTTGTAGTGTTCAATCCCACTGAGCAGTACGTGAATGCCAACTTT
TCCAATGTTGTTGGGATAGCTGAGGAATTGACTGTGCACACTCTCAGTACCAATTACAATGCCACAGA
TGTGGCTGTAAA
>LLOTMP006803-E3 exon: PUTATIVE_protein_coding
GGGAAAGGTGTCCAGTTCATCAATTCCTGTTTCCAGATACTCTGCCATAATCCTAACATACGTCCCAAAA
GCTTAA
>LLOTMP006803-Intron 1:PUTATIVE_protein_coding
GTAAAACAGAAATCCCGCTTTATATAGTTGACTCATATTTTCTGCTGCTAACAAGACTAATCAAACTAAT
CATTTCACATGTTCTTTCCTCTCTGTGGCATTAACCATTTCTCACTAAAACATGGCTAACAGAATTTGGT
AAATGATTTCACTAACACTTTTTTTTTAACCATCTGGCAATGAAAGTTCTTACTCTATTGTTGTTTTATA
CTTTTAG
>LLOTMP006803-Intron 2:PUTATIVE_protein_coding
GTGAGTTTCTCTTCTTACTTCTCAAAAAATGATCCATTTTAACATATTTTTTTTTAAAATTTCTTTCAG

>LLOTMP008629/ LlNBAT1
>LLOTMP008629-E1 exon:PUTATIVE_protein_coding
ATGAATAATTTGGGTATTTCGACGGATCCTGCTCTCCTTGGGGTGGATTTGCCCTCATCACTCACGACCT
CACCGTCTGTGTCAACCTTCATGCCCGAAGAGGATGCATCCATATGTCCACTCCTCCCAACAACACCCAG
CCCACCGCCAATGGATTTTATACATCCCCTCACGCCAAGTACGGGGATTGATGAAGGAAATAATGAAGAT
CTCGGTGGTGAGTTTCAAG
>LLOTMP008629-E2 exon:PUTATIVE_protein_coding
GTGATGATGCTCAAGCAGATTCAAGCTCTTCAGGAAGTAGCTCTGGCATTGGGATTCAAGTCACTGCCAA
TGGGCCAGGATCTCTCTTCAATAAGCACGCATATCAGCATTTGGGCAGCAAAAATGGAGACATTACGCAA
>LLOTMP008629-E3 exon:PUTATIVE_protein_coding
GACAATGGTGTTACCCAAACAGGAGTCTTCAGCATCCCCATGACAAAGGATACACCGTCTTTTGTGAACT
GGAACTGGCCTCTCATCCGCAAATGTACCTTCTTCCTCTTCATGTCCGGTCTCTTTGCAATGTGTGCCAT
TGTTGTTGCCATGATGTTCAAATTACCCAAATCCTGCAATCCCGAAGTACCCTGGTACAAGGGAGCTGTC
TTCTATGAGATCTTCCCGGCGAGTTTTCAGGATTCCAATGGGGACGGTTTGGGTGATTTAAAGGGCTTGG
CGAGTCGCATTGATTACCTCGAGAGTTTGGGCGTTGGAGCTGTACGGTTGAATTCAATATTCCCCGCCAA
GCACTATCCGGATCACTTCCAAAACGTCACAACTCTCCTGGACATTGATGAAATTCTGGGAAATCCGAGG

GATTTAACGTATTTGGCGCGTATACTGCAAAGACGGAATATTTCATTGATCCTGGACCTTCCCATTTATC
CTCTTGTTAAGCATCTCGCTGTAATTCGTGAGAATTTCACGGAAGCAACAAACTCAACTGACACAGAAGA
TGAGGAAGTGTTCCAGGATAGTGACACTTCGGAAGATCCTGTACTTACAGCTATGCGATTTTGGCTCTCA
ATGGGTGTGGATGGATTCTATGTGAAAGGACTCGAGAACTACGCAGGTGATCCGTATTTAATTGAGAATC
TTCAAGAGTGGAAATTCGCTTTGGGCTCAGATCGGATTCTCATGGTTAGTAAACTCGTCTTTGACTCTGT
GGATGATGACACGGCGGAGAAGGTGAGGCATTGCGTGGATTTGGTGGATGTTTTCGTGGATGTCTCCAAT
GGGACAAAAGTCATTGCTGAGAAGGTTCAATCAATCCTCAATTCTCGCTTCCTGGCACCTGGTTTTGGTC
CATGGATTCACTGGAGTTTGGGTGGTGTAACCGAGAGAAGATTGGCCCAAGGAACAAGCTCAAATATTTC
TCTAGCAGCCACACTCATGCAGCTCATGCTTCCAGGAACTCCGAGCATTTTCTACGGAGACGAAGTTGCT
CTGCAGGAAGTTTATGATCCTCTCGGGGAGCACGAAGAGTCCAAGCATCTGCATCATCTCTCAACAATGG
TCTGGGATTCGGAGATTCAGTTCACGGTGAAGGAGCATTTGCCTTGGCTACCTCGGGGAGCCTTTGTGGC
TTTCCATCATTTTGAACATGTGGCAGGGATGATAAGACTTCGTGGAATTTCTCCTTCCATCTACCAGAAT
GCCGTCATAAAGGAACATCAATCGGTTCTCAATACTGCTGTGAG
>LLOTMP008629-E4 exon:PUTATIVE_protein_coding
ATACAGCAAAAATGACATTTTGATCCTCGAAAGATGGTACCCCAGACGAAATACCTTCACATCAATCTCA
AATTTTGGAAGTAAATCCCTCGTCCTTGATCTCTCTGGGATGTTCTATGCGGGAGAAATAATGCTGGGAT
CCCTCAAGGGGGAAAAGGTCTTCTTCAGCAGTATCCAAGTGAAGCCAATGGAGACGATTATTGTAAAGCT
GGACAAGTAA
>LLOTMP008629-Intron 1:PUTATIVE_protein_coding
GTGAGTTTGTCTTGATTTTTAACATTTTCTTAACTTTTTAATTAAATTCTCTTTTTTCCATGAAG
>LLOTMP008629-Intron 2:PUTATIVE_protein_coding
GTACGTCTTATGCTAATACATACGCACATAAAATCAATTTTATCAGACCTTTGATCAAAATTTGATGAGA
AATGGAAATGTTTTAGAATTGAATTTTGTAGGAAAGATTTTCTTTGTAAGATTTTTTTTTAAATGACTCC
GCGAACAAGATTTTCAAAATTTTGATTAAAAAATCATAATTTCATAGCAAAGACAAATTTTATTTTAACT
ATAAAATAGTCAGGACACATGTTGTGCTATAAAAATTCTGATATTTTGACTAAATATCTAATGAAATTAA
AATAAAAAGGATTTAAAGTACAAATCAAAAGGACGTGTTAGTTAAGGATCTTTTTCAATATTTTCAAGAC
CTAATAAACTAATCTAGACAATTACCCAAAGAATATTTCTATAAAAGATAATTAATTAATTAATTAAATT
TTAATTTTAG
>LLOTMP008629-Intron 3:PUTATIVE_protein_coding
GTAATTATTTTAAAGGATATTTTTGAAGAATAATCAAAGTATTAGAAATAATTTTAAAGAATCTATTCGA
AGGATTCGGTATTATATAAAATGTTTAAAATATTTTAGAAGAATCTTCAAAACATTCGGAATATTTAAAG
TATTCGGAATATATTTGAAGAATATTCAAGGGATTCGAAATATTTAAAGAATATTCAAAGTATTCAGAAT
ATTTAAGAAGAATATTTTCATTAAAGAGAATATTTATGAAGAATATTTCCGAAAATCAAATTATTTTAAA
GAATATTTTCGCAATATTTTATATAAAACATAATTTTGACCCAACCTGCAGGAAATTGAAGTTTGTACAC
TTTTCCTTCCCGGAACAGTTGAATGAGCTTCCCGGAAAAAGATTTAAGTGGCTGGAAATTGTGTAGTTAT
CCTGCCCATTGCACCAGAATATTTTTAAGGGATCTTAGGAAACTCCTTTTTAACACCCGGCTGATCCTTG
ATGTCATCTTTGGGTCAAAATCTGTCCATTTAGGAAGTCCCTTTAGCAATTTCGTGAAGCATTTTTTTAA
ACTCAGACTTTGACCGAAAGATAACATCAAGAATCAGCCGGGTGTTGAAAAGGAAGTTCCTAAGATCCCT
TGAAACTTCCTGATATAATGGGCAGGAAGACTACTCAACCTCTAGGCACTTACGGCACTTTCCAAGAACA
TATCAAACCGTTCCGGGAACAAAAAAGTCTATAAACTTTAATTTGCTCCAACCTGCTTCAATTTCTAATC
CATTTTTTTAAAGAATTTAAAGAGAAAAATTAAAATAAAATTCTTAAAGCTTCGTTTAAAAAAAAAACAA
ATAAAACACATTAAATGTTTTTTTTTCTAAATTCCATTTCAG

1,4-alpha-glucan-branching enzyme
>LLOTMP005533/ LlAGB1
>LLOTMP005533-E1 exon:PUTATIVE_protein_coding
ATGGATCCCATGAAGGTGGAAGTGCCGGAGATTGACAAATTGTTTGCCATCGATGGTTACCTGAGGCCAC
AAGAGAGGGAATTGCGAAGAAGG
>LLOTMP005533-E2 exon:PUTATIVE_protein_coding
GCATGGCGTGATCAAGGATTGGCTAAAGAAGATAGATGGGAATGAGGATGGGGGTGTGGATGGATTTTCA
CAGGCCTACAAATACTATGGGATTCACATTCAGCCCGATAATGCGGTGATTGCCCGCGAATGGGCCCCTG
GGGCGCAACAACTCTACCTCACCGGTGATTTCA
>LLOTMP005533-E3 exon:PUTATIVE_protein_coding
GATGATTGGCAGTGGGAAGCCACACCGTACACAAAGCTCGAATTTGGGAAGTGGGAATTGAAGATCCCAC
CACGAGAGGATGGCTCGTGTGCCATTCGGCACCTTTCGGAGATTAAGGTGATCGTTCGCACGCAAGAGGG
CACCCTCGTTGATCGCCTAAGTCCTTGGGCAAAGTACGTCCTCCAACCACCGAAGGAGGCTAATCAGGGT
ACGAACTTCAAGCAATACCTTTGGCATCCGCCGCCACATGAGAAGTACATGTTCCGCTATGGACGCCCCC
AGAAGCCCCAATCGCTGCGCATCTACGAATGTCACGTTGGCATTGCCACGGAGGAGCTTGGTGTGGGGAA
GTATCGGGATTTTGGGGATAAAATCATTCCGCGAATTGTCAAGCAGGGCTACAATGCCATTCAGGTGATG
GCAATTATGGAGCATGCGTACTACGCTAGCTTTGGGTACCAAGTGACGAGCTTCTATGCTGCTTCCAGTC
GCTACGGGACACCTGATGAGCTGAAATACATGATTGATATGGCCCACAAGGCTGGTCTCTACGTTCTCCT

TGACGTTGTGCATTCCCACGCGAGTAAGAATGTTGCCGATGGGCTCAACCGCTTTGACGGCACCAATACG
GGCTTCTTCCATGATGGCGCCCGCGGGGAGCATCCCCTCTGGGACAGTAGACTCTTCAACTACACCGAAT
ACGAAGTTTTGCGTTTCTTGGTGTCCAATCTCCGGTGGTGGCATGACGAGTACAACTTTGATGGGTACAG
ATTTGACGGCGTTACGTCAATGCTGTACCATTCACGTGGTGTGGGGGAGGGTTTCAGTGGGGACTACAAT
GAGTACTTTGGCCTGAATGTGGATACAGATGCTCTCGTTTATTTGGCCATTGCCAATGAGATTCTCCACA
AACTTGACAAGGATATCATTACAATTGCTGAG
>LLOTMP005533-E4 exon:PUTATIVE_protein_coding
GACGTGTCTGGGATGCCAACAATGTGTCGTCCTGTCTCCGAAGGTGGCATTGGGTTTGACTATCGCCTGG
GTATGGCCATTCCTGACAAGTGGATTGAGTATCTGAAGGAGAAACGTGATGACGACTGGAATATG
>LLOTMP005533-E5 exon:PUTATIVE_protein_coding
GGTAACATCGTTCATACCCTAACTAATAGACGATGGATGGAGAAAACCGTTGCTTATGCTGAATCTCACG
ATCAGGCACTCGTTGGGGATAAAACTTTGGCTTTCTGGCTGATGGATAAGGAGATGTACACGCACATGTC
GACCTTGTCGGAGCCTCCGTTGATCATTGATCGTGGCATTGCTCTGCACAAAATGATCCGTTTGATCACG
CACTCCCTCGGCGGGGAGGCGTATTTGAATTTTATGGGAAATGAATTTGGTCATCCGGAATGGTTGGACT
TCCCGCGTGTTGGCAACAATGATTCCTACCACTACGCTCGACGTCAGTGGCATCTGGTTGATGATGAAAT
GCTCAAGTACAAGTACCTCAATGAATTCGATCGTGCCATGAATGGGTTGGAGATGAAGTACGGCTGGTTG
GCATGTGATCCGGCCTACGTGAGTTGCAAGCATGAGGATGACAAGGTGATTGTCTTCGAGAGAGCTGGAC
TCCTGTTTGCCTTCAATTTCCATCCATCGAAGAGTTTTACGGACTACAGGTTGGGCGTTGAAGTTGGTGG
CTTGTACAGGATTGTCCTGAGTACGGATGATCCCCTCTTTGGTGGTATGAATCGTGTTGATGTGAAATGT
GATCATCTCTCAACGCCCGAAGGGTTATGCTGGAAGACGGAATTTCATTCAAGCCTACCTTCCAGCACGA
ACTGCCTTCGTATTCGCCAAGTTTGATTAG
>LLOTMP005533-Intron 1:PUTATIVE_protein_coding
GTGAGCGTAACCGGAAGTCTGTTCTGTTTTCCCCAAAAAAGGATGAAGAATCTCCACTGATTCCACATTC
TGTGAGAGATGCTTCAATTTTTTTCCGTTGCGGGCATTTCCCAATGTCACGGCAAGGTCAATTTTTGGCT
GCTATCGCAAAATGAATTTATATCAATAAAAAAAAAATAGAAAATTGATGTTTTTTCTCTTATTGCCAG
>LLOTMP005533-Intron 2:PUTATIVE_protein_coding
GTGAGTTCCTTTTAGTCGCTCTTAGGTGAATTCTTTATGTGAATTTTGGTTATTTAAAGAATTTGTGTAA
GAAAACGGTTAGACATAAATCCAATAAGACTAAATATAGGGGAGAATGGCCTAAATTCGACAATGAGATT
TTTTTTTAATTTAATAATTTGTATAATGTAAAATAAACTGCACAAAAAGTTGTGAATCGTAGGGTAATGA
TAAACGATAAGGGCAAATAAAAAGGCGATAAGTCAATTATAAATGATTTAATATAATAACTTAAAATTTT
GAACAATTATCCAAAAAAAAAAGAACTTAAGGTGATTTATTTGCATATTCGCGCATAAAGGCTTTATATG
CTGCATACTTCTCCTTCACTTCCTCCACTTCCCGCTGCAGCTCGAAATTTCTCGCTGGAGGAGGAGATTC
TCCTCCTGAAGACGTGCGTTGATCTCCAGAAACTTATTGTTGCTGTTCTTTATTAAAAAATTAAATTAAA
AAATTGATACGTCATCTAACAGAGCCAGCAGAGCCTTATTGCACTCCTTCGCCTGGTTGATGGCTTGGTC
GAACTCCTTTTGCACCTCTTTTGGATCCATCTTTCCTCCTTTTTTTGACTAATCAAAATTTTCTTCCTTA
CAACGGGCCTAATTCAGCGACCAAGAAATCCTTGAAATCATGAAATTGCTGTACAAAACTCAAAGATTTC
AAGGATTTCTTGGTCGCTGAATAAGGCCCGGATATATCCTTCCTTTTGACTGTGATTTTTTTTAATTAAA
TTTTTACTCTGTACTATTTACTGTCATTATAAATGCCAGATACCTGGATGTTATTTATGTAGCCGTCCAG
GAGGTTTTGGATTGTCTGCATCGACAACCGCCTTTTTGTCCTCTGTCACCTCGTCGAAAAAAGCTTCTAG
CGCTTCACGATTCATGTTTTGCTCTATATCTTTGAAACCGCCACATAAATTTTTAAGATTTTTTTTTTGG
CTAAAAGGTGTGTCTGTCAGTTAAGATATACTAAAATTTAATCAAAATGTATCGTCAAGTTTTTGAGATA
TTAATCGAAAAATTGTCGAAAACTCATCGAAAATTTTGTTTTTGATTGTAGGCCCCCTAGCGGTAACTTT
TAAAACTTCCTATGTACAGATAATTGTAGACCTTCTTGAGATCTTCCACTTCTTAATGTTTTTCCCCACT
CTTGATGTTATTCAACACCTTTTGATCTCATTCACCACTTTTTGATTTCTACACACCACATTTCCATTCA
ATTAATCTCAAGGCGAAAAATCATAAAATTATGCAAATAGCTTAATAAAAAAGGGTCTAAAATTCGTTCA
AATGATTTAAATAATGTAAAAATTCCACCTCCA
>LLOTMP005533-Intron 3:PUTATIVE_protein_coding
GTGAGTTTGAGAATCTCATTTGCAGCCCTTCGTTGACTCTCCTTTGTTCCTCAG
>LLOTMP005533-Intron 4:PUTATIVE_protein_coding
GGTAAGTTCCGCAACCTAAACGATTTTTTGTTGTTGTATTCGTTAATTTTTAAATCTTATTCACCACTTC
TTGATCTTTTTCACCGCTTTTTGACCTTCTTCACCACTTTTTGACCTTTTTCACCACGTTTTGATCAATT
CACTACATGTTCAATTTATTGTCGATATTCTTGTCTTGATTTTATTCACAGGCTGTGCGTTAATTATTCA
ATCTTATTCACCATATTTTAATCTTAATTCACCACGTTTTGATTTATTTACCCCTTTTTTAATTTTTTAT
ACTCTTAAATATTCTTGTCTTGACTTTATTCACCACTGTTCGATTCTAGGATAAAAACTAGACAGTCTTA
AATATCTTGTTTTCTTAATATTAACCTTTGAATGCGGAGGCCTTGGTAGTTACGTAGAATGCGAAGGTGG
GGTCGTTGAACGACCCCAAAAAGAAGGCCAATTTTCTCCCAAACTATACAACCTGGAGTTGTCGGGTTAG
TGCCTATAGATTCCTTATATAGTCCTCTTGCACCCTGCACCACAAATTCGCCGCCCTAAAATACTTAGAA
GGAGATATTAGGGAAAAACATTXXXXXXXXXXXXXXXXXXXXXXXXXXXXXXXXXXXXXXXXXXXXXXXX
XXAAAACTAAGAGAACTTAAGATGAAATCTTTCTTAACTTTTAAGAGAACTTAAGAGGAAGTCTTTCTTA
ACTTTTAAGAGAACTGAAGATAAAATTTTTCCTAACTTTTAAGAGAGCTTAAGATGAAGTCTTTGTTAAG
TTTTAAGAGAACTTAAGAGGAAGTCTTTCTTAATTTTTAAGAGAGCTTAGAATGAAGTCTTTCTTTACTT

TAAAGAGAACTAAAGATACAATTTTTCTTAACTTTTAAGAGAGCTTAAGATGAAATCTTTCTTAACTTTT
AAGAGGACTTAAGATGAAGTCTTTCTTAAGTTTTAAGACAGAGGAATTATCAGCAATGATAGCATCCCCT
TTAATAAATTATTAAAACTTATGATTCTCTTAATTTTCTTAAGTGAAGCTTAAAATCTTTAATTTTTACA
AAATTACTTTAATTTTCTTAAGCAAAAGTTACGAATTTTAAGATTTCATGAGATTTTTAAAGTTTTTTTT
AATTATCTCAAAATTTCTTAAGTTTTTTTAGGCGAAAATTAAGAGTTCTTAAGATTTTAAATGTTTCTAA
AGATTCTTTTTAAAATTTTCTTTGGAAAAGCTATGAATTTTTAAAAAGTATTCGCGAGAGTTAAGATTTA
TTAAGTCAGGCTGAATCCGAAGATTTTTACGATGAATATTAATTTAACCCAATTCAGTCTGACTAAAGAA
ATCTTAAATTTTTCTTAAGAAAACTTAGGAAATCTTTAAAGAATCTTTAAAGAAAACTTAAGAAATATTT
TCTAAGTTTTAAGAGAACTTTGAACTTTAGAAAACTTAAGAACTTTACTTAATATTCCTTAAATTTTCTT
AAGCGAACTTTCTTCAATACTCTTCAGCGAAAGTTAAGAGTTCTAAGGATTTTTTTTAATAATTGTTAA
TAATTTATAATTGTTTATTCACATTCCATTAAGATTTTACATACTTTATACAAATTTTCTCTACAAATTC
AATTTGCGATATTTTCGTAAATTTCTCAAAATTTCTTAAGTTTTCTTATAGCAAAAGTAACAAATTTTAA
GAATTTTAAGAATTTTAAGATTAAAAAAAATTCTTAAGAGAAAATTAAGAGTTCTTAAGATTTTTAAAGA
TTATATCAACCCTAATCTGATTTTTTTTCTTCTA

Glycogen debranching enzyme
>LLOJ008312/ LlGDE1
>LLOJ008312-E1 exon:PUTATIVE_protein_coding
ATGGGGCATCAGGATGTATTGAGTTTGGCCATTTCACACGGTGAGGATAAGGAAGGAACGCTCTTCAGGC
TCAAAAGGGGTACAATTCTGCACATTGTTCCTGGACCTAATCTCCTTGGCAGGCACATCGCATTGTACTG
CAACTATCCACAAAATGGTGAGAAA
>LLOJ008312-E2 exon:PUTATIVE_protein_coding
GGTTTTGAAAGAACAAAATATTCAACTCTAATTTGGCACACAAGTTCAGGTCAAAAGCTCACAAATGCGG
CTCAACCCTTTGTCGAAGTTAATGATCTAGATATTTACTGCGAAATAGTGGCTAACAAATCAGGAACTTT
TCATTTCTACTTTAGCTTCCAAGAAAG
>LLOJ008312-E3 exon:PUTATIVE_protein_coding
TCCAAAAGAACGAGAAGGTTCCCTTTACGTTCAAGTTGAACCAAAGATTAAAGTCGGACCGAAGAATGCA
GAACGTGAGATTCCCCTTGACTCCATACGATGCCAGACTGTGATGGCAAAATGTCTGGGGCATTTGGAGA
CATGGGAAGCTAAGTTGAGGGTTACTCATGAGAGTGGCTACAACCTCCTTCACTTCACACCCATTCAGAA
GCTAGGAAACTCCCGTTCGGGATATTCTCTTTGCGATCAACTCAGCGTCAATCCAGACTTCGGTCCTAGT
GCAAATTTTGATAAAGTCGCAAAAATCGTTAAAAAATGTCGCGAAGAATGGGGCATTGCCTCTATCTGTG
ACATTGTCCTCAATCACACAGCCAATGAATCTCCCTGGCTAAAGGAACATCCAGAGACAACCTACTCATG
CAGTACTTGTCCACACCTTAGACCTGCTTTCCTGCTCGACAGCGTCCTTGCAATGGCGTCGAGTGATACA
GGAAATGGTCTTCTGGAAACTTTCGGTGTACCTGTTGAAATTGATCGTGAAGATCATATCAACGCATTGC
GTCATCAACTGCATTCAAACTACCTTCCCAAGGCTAGAATACATGAACTGTATCAGTGCAATGTGGAGGA
GTATGTTAAGAAATTCAGTGAAGAAATGCGCAAGAGACCTCCGCCAAAAGCTCCAAAGGAATCCCAATCA
AAGGAGACAATCGTCTTGAAGCAGGATCCTGCCTATAGACGCCTTGCTTGTACAATTGATTTTGAGAGTG
CTTTTGAGATCTTTAATGTTTTTAG
>LLOJ008312-E4 exon:PUTATIVE_protein_coding
GAATGACGCCTTTGATGAAGATACAAGATTGAGGAAATGCGCTGAAGCCTTTAGGCAACACTTGGAGGAG
TTAAATGAAGTTGTTCGTTGTGAAATAGCTGAACATCTTCGTTCAGCCGTTGAAAATTGTTTATCCGGTC
TCCGATATGAAAGGATTCAGGATGATGGTCCTAGACTACGGGAGATCAGCATCAAACATCCACTGTTCTG
TAGATACTTCACAGATGGTGATGTTACGTGTAAAACAATTGAGGATATTGAAGCTCTCATGTACGGGGAT
TCGGCAAAGTATCTTATGGCTCACAATGGATGGGTTATGAATGGGGATCCCCTGAAGGACTTCGCTAGGC
CCCAGCCAACAACTGCGAATGTCTACATTCGACGGGAGTTGATTGCTTGGGGTGATAGCGTTAAGCTCAA
GTATGGGGATAAACCCGAAGATTGCCCATACCTGTGGGATCACATGAAGAAGTACGTTGAGATAACTGCT
AAAATTTTTGATGGAGTTCGTCTTGATAACTGCCATTCAACTCCGCTGCAT
>LLOJ008312-E5 exon:PUTATIVE_protein_coding
GTTGCAGAGTATCTTCTGGATGCAGCACGAAAGATTAATCCTGAACTCTACGTTGTTGCGGAACTCTTTA
CAAATTCCGACCATACGGATAATATTTTTGTCAATAGATTAGGGATTACATCGCTGATCAGGGAGGCTCT
TTCCGCATGGGATTCCCATGAAGAAGGGAGACTTGTTTATCGTTACGGAGGAGCACCTGTTGGGGCATTT
TTCAGCTCTCCCAAGAGACTCCTGGCGCCGTGCATTGCTCATGCTCTCTTTCTCGATCTTACACACGACA
ATCCGTCCCCGATTCAGAAACGATCGGTCTTTGATCTGCTCCCCTCAGCAGGATTGGTTTCAATGGCATG
CTGTGCAACTGGAAGTACAAGAGGATATGACGAACTCGTCCCTCATCAT
>LLOJ008312-E6 exon:PUTATIVE_protein_coding
ATTCACGTCGTTGACGAAGAAAGGGAGTATCAGGAGTGGGGTAAGGCTGTTGATGCGGAAACTGGAATAA
TTTCTGGCAAGAAAGCAATTAATCTTCTTCATGGAGAACTAGCTGAGAAGGGTTTCAGTGAGGTATTTGT
TGATCAAATGGACTACAACGTTGTTGCTGTTACCAGGAGTTGTCCTACTACGAGAGAAAGTGTCATCCTG
GTTGCACACACCTGCTTCAGTTATCCAGATCCATACAGTGGACCTACAAATGTTAGATCTCTTAGATTTG
AAGGGCATCTTGAGGAGATTATCCTTGAAGCAGGAGTTACTCACAA
>LLOJ008312-E7 exon:PUTATIVE_protein_coding

ATCTTCAAAGCCATATGACTGTCCATATAGGTATGAGAAAGATGAGAAATATATCAATGGACTCACAGAG
TATCAGTTGAAGATTGTTGAGCATATTCCTCTGGAGAAATCAACAATTTTCAGCACAGAAATGGTTAAAG
ATGGCAACATAACGCAATTGAATTTTAAGAATTTAACTCCCGGATCAATTGTTGCTGTTCG
>LLOJ008312-E8 exon:PUTATIVE_protein_coding
AGTTTCCCTGCATGAGCGAACTCGTCCCCATTTTGCCAATGTTCAGAAACTCGTGGATGCTTTTCACTTC
CAAAAAGGCCAGATCTTCAGTGATCTCCAGAAAATTGTGTCCAAACTCAATCTCATCGATCTCAATCGTG
CTCTCTACCGTTGCGATGAAGAGGAGAAGGATATGGGAATGGGGTCTGGAACTTATGACATTCCCGGATT
TGGGCGGTTGGTTTACAGTGGAACTCAGGGATTTGCATCAGCTTTGAGTGTTATTGGGCCTAATAATGAT
TTGGGGCATCCATTCTGCGATAATCTCCGTCGTGGGGATTGGATGATTGACTACATACGCGATAGATTAG
CTCGTTGGTCTGGGACTAAAGCTCTTTCAGAATGGTGGGCAACCAATACGGCATCCTTGAAGGAAATGCC
TCGTTATCTGATTCCCAGCTACTTCGATGTTATCCTCACGGGAGTTAATGCTCTTCTCCTGGAACAGAGT
GTAACGCTAATGTCTGATTTTGTCCGGAAGGGAAGTTCGTTTGTGCAATCTCTGGCCATGGGATCAGTTC
AGTGTGTTGCTGAATGCCCATCAGCTAATCTTCCGGCTCTTAGTCCGCAAACAAAGGCTCCCAAGCCGCC
AAATCAGTGTGCAACTATGTCAGCGGGATTGCCGCATTTTTCAACAGGATACATGAGATGCTGGGGCAGG
GATACCTTCATCTCCCTCCGGGGACTTCTTCTCCTAACCGGAAGGTTCGATGAAGCACGCTACATGATTC
TTGGATTTGGTTCCTGTCTAAGGCATGGACTTATCCCGAATCTCCTCGATGGTGGCTACAAAGCAAG
>LLOJ008312-E9 exon:PUTATIVE_protein_coding
ATTCAACTGTCGCGATGCCATTTGGTGGTGGCTGTACTCCATTAAGTATTTCGTGGAGGAGGCTCCAAAA
GGAGTTGAAATCCTCACGGAAAAAGTCTCCAGGATATTCCCAATGGATGACAGTGAAGCCCGCAAGGCAG
GGGAATGTGATCAAGCTCTCTGTGATGTAATCCAGGAAGCCATCACTGTACACTTCCAGGGTCTTGTTTA
CCGTGAAAGGAATGCAGGTCCTGCCATTGATGCGCACATGACGGAAAAGGGCTTCAATAATCAAATTGGT
GTTCATCCTGACACGGGATTTGTCTTTGGGGGAAATGATGCAAATTGTGGGACGTGGATGGATAAAATGG
GATCGTCAGAGAAGGCAAGGAATCGCGGAGTTCCCAGCACACCACGAGATGGATCAGCTGTGGAGTTGGT
TGGTCTCCAAATGGCCACCCTCAGGTTCCTGCAGAAGATGAGCACTGAAGGGAAATTCCCCCATAAATCT
GTTGAAAGAACATCCAAGAATG
>LLOJ008312-E10 exon:PUTATIVE_protein_coding
GAACCAAAACCGTTTGGACGTACAAAGAATGGGCTGATAGGATAAAAGCGAATTTTGAGAAGAATTTCTT
CGTGGGACAAGGGGAAATACCTTTGGCTAACAAGAGAGGGATCTACAAGGATTCATGCGGAGCTACGCAG
ACGTGGCAGGACTTTCAACTTCGTCCCAATTTTCCAATTGCAATGGTTGCG
>LLOJ008312-E11 exon:PUTATIVE_protein_coding
GCTCCTGAATTATTTGACCCAAAGCACGCATGGGAAGCTCTCGAACAAGCTCGAAAATACCTTCTTGGTC
CGCTGGGGATGAAAACTCTCGATCCTCAGGATTGGGGATATCACGGCGACTACGACAACTCCAATGACTC
TGAAGATCCAAAAGTAGCTCATGGAGCAAACTACCATCAAGGCCCT
>LLOJ008312-E12 exon:PUTATIVE_protein_coding
GAATGGGTTTGGCCAATTGGATTTTACTTGAGGGCTCGACTGATCTTTGCCGCACAGAATAATCGCCTGA
AGGATACTGTTGCTGAAACATGGGCCATCCTCACAGCTCATCTCAAGGAGGTGAAGTCCTCCTGGTGGCG
AGGACTCCCTGAATTAACAAACTCCAACGGAAGCTTCTGCAGTGGTTCCTGCACAACACAAGCATGGAGT
ATGGCAACTGTCCTGGAAGTTTTGTACGATCTGGAGCGTCTTAAATGTAAATCTTAG
>LLOTMP008312-Intron 1:PUTATIVE_protein_coding
ATTTCTTTAAATTCATTACCTAAAGCATGAGGCATTTGCTCTAATTTTAGCCAGTATGATGGTACAACAT
CAACAACAACTACAAAATGATCTTGTTGATCATGTGTTGATCAAGTCTAGTCATCAGGGTTGTTTACATT
TTTTTTGTCAAGATCTTACTATGTATGTTGCAACTTGTTATCTATTAATTATAGTTATTTTATTAACATG
TGAAATAACAATTTATAGCGTAAATTGGTCGTAACATTTTTTAAGGCTTTATTTGAAATTAATCTAAACT
TTCAATAACAAACTTAGGGTCTTTTTACAAATTAATTATCATTAAATAAAAATTTAATTTGTCTGACAGT
CACAGCTTTTTCATACAGCTTTTTTTCTTTAAACTGAATTTATTTACATTTCTTCAATAAATTTTAATTA
TTCCACGCAAAGCTTTTTGATAGAGACATTTATTTTGATTGCTTTTAATAAAACATGCTATAAAATTCAC
GTGGTTGTATTCTTGACCCAAATAAATACTTAAAAAAAAAGTTATTTCAGTGGAAAGTTTTAGTAAGATT
TAGTAAAATTTTAGCGTGAAAGAAGCTTAGAATCATTTTTTTAATTAAAGATATTCTTATAACTTTTCAA
AAAATTATTGAAAAGAGCGATAGAGTTTAGAATAGATTCCATTTGCATTTATGAGCTCATCGTGAGTTCC
CATCTCGGTGATCTTTCCTTGATCCATCACACAAATAAGATTAGCGTTTCTAATGGAGGATAGTCTGTGA
GAAATGATCAAGCAGGTTCTGTTCTCACTTGCTCTATTCAGAGCATCTTGAACTGTCTTCTCACTTTCTG
CATCCAAAGCTGATGTTGCTTCATCCAGCAGGAGGACTTTTGGCTTCCTGATAAGAGCCCTTGCAATGGC
TATCCGTTGTTTTTGTCCTCCCGATAGAAGAGTTCCTCGTGATCCAAGACGTGTTGCATAGCCCTAAAAA
TTCGTTTTTTATATCAAATTAAGTCTTAAATTATTTTTGTGAATGTTGTAACTTACAAGAGGTAGTTTTG
TGACAAATTCCTTATGTATTTGAGCTTCTTTTGCTGCTTCAATAACTTCCGTCAGAGGTACATCTCGTGA
ATTGTCACCGTAGGCTATATTTTCAGCAATAGTACAGTCAAAGAGAACAGGCTCCTGACTTACAAGACTT
AGGTAGGCACGAAGCTTAGAAAGAGAAAATTCCGTCGTTGCTGCACCATCCACAGCTACTTTTCCCTCAT
TAGGATCGTAGAAGCGAAGAAGCAGTTGAAAACATGTTGATTTTCCACATCCTGAAGGACCCACAATAGC
TACGGTTTTCATTCCATCAACATGGAAGTCTAGACCTTTAAGGATTTCCGTTTCTGGTCGATTTGGATAA
CTAAATTTTATATTTTTGTATTCAATATCTCCAGAGAAAACCTAGAAAATACAAAGTTTTGTTGAAAGAT
CTTCTTTCTTAAAATTGCTTAGTCTTACCATTTGGCCGTCATGCTTCATGCTCTGAGCAGTTTCTGACGA

CTTTGTTGAGGTGTCTATAAAGTCCATGATATTTGCTGCAGACGTTAGAGCAGAATTAACATTTGGAGCA
GTATTGAGAACAATTCCCATCATCCATGCTCCATAAATTATAGCTTCGATTACCCTAAAAGGATAAAAAT
CTTATTCAAATCTTGCCCTTATTCTTAGATCCTTCTTGTATTTACTTACATTAAAGCATCAATTAAGGAT
ATTTCTCCGGTTGATATCAAATGTCCAGCGTAGATAAAAGTAACGCCGTATGCAAAGATACTCATTGAGA
TATTTAGACTATATGCTAGACCTCTATATTTGGTACTCTTCCTTGCTTCTCCTTCTGCTATTTCAATCTT
TTCAATAAATCTCCGAAGTACGTGTTTCTCCTGACCAAGGGAGGCTACAGTTCTTACGCTCATAATTGCT
TCAATTGCAATCCTTGAAGCATTTTCCATTGTTTCCTTCTCCTTTGCTGCGCTTGATTTTGCATAGAGAG
ACTCAAAAGCAGCAACAAAGATGAGCAAAGGTACGGTTAGGGAGACAACAAGAGTAAGCTTCCAGAAAAC
AACTGATCCTACTATAACGCCAATTAGAATGGATGAAGTAGCTTGGAGGATTCCAGCTAAACGAATCCCC
GTAGCTCCTTGAACATTAGCACAGTCAGTGGAGAGTCTAGCAGATAATGCGCCAACGGCATTGCTTGGAT
GATCATACCAAGCTATATTCTGCTTTATAGCAGTTTTGAAACACAAATTCCTAAGAAGAGAAGTAAGACG
AACTCCAGCTTTCCCGAAAACATACGTTTGGGTTACTGAAGCTATTCCAGTTACAGTAGCTAGAATGAGG
AAGTTTATTCCACATTGAAATTGAACCACATGAATCCTGTCTAGGTCTGTTGTTTCAAAAGCTTCAAAAA
TTTCCGCATTTAATACACCACTTATGGGGTATGATGCCCCAACGATGATTGAGCAGATGCATCCAATAGC
TAAGAGCTTCCAATCCTTCAAACTTATTCTTATAAGGCGCTTCATTGTATTTTGCTTCATTTTCAGAAGC
TCTTCTTTGTCTTTGACACCTACAATATCCAATTGTCCAAAATCTTTTGATGGAAATTCACTTTCTTCAT
CAATTTTATCGGTATTATCAAATTGTAGACCATCATTGAGATCTGAGAATGCAACCTCTTGGCTAGCCAT
TCGTAGATCATGGTAGATTCCTTTTTGAAGCATTAATTCATTATGCGTTCCAACTTCTGCGATTTTTCCC
TGATCCAGGACTATTATAAAGTCAGCATTTCTTATGGTTGAAAGTCGATGGGAAACAATTAGAGTTGTAC
GACCTTTTAGAGCCTTATCCAGAGCAGTCTGGACTAATTTCTCCGACTGAGCATCCAAGGCTGAGGTTGC
TTCATCAAGAAGCAAAATTGCTGGGTTCCTAACAATGGCTCTTGCAATGGCAATTCTCTGTTTCTGCCCT
CCAGACAGTTGGGCTCCCTTTTCTCCAACCACAGTGTCGTATCCTTGCGGTAGCTTTGTAATGAAACTGT
GACAGTTTGCTATCTTGGCTGCATCTTCAATTTCACTTTGAGTTGCTTCAGGATTTCCAAAACGAATGTT
TTCTGCAATGGTTGTTGAGAAGAGGACTGGTTCCTGATTAACTACGCCTATTTGGGATCTCAAGAATCCT
AAATTGTACTGTTGGATGTTGACCCCATCTACAAGGATTTCCCCCTGAATTACAAAAAAAATTAAATAAA
ATGAAAGAAGAGTTTAGCGTCTTCTCCTTAATATTATCTAAGATACTTACATCATTCACATCGTAAAGTC
GTTGAAGGAGTTGCAAACACGTAGACTTTCCACACCCAGAATGTCCAACAATAGCCACCGTCTGACCACT
GTTGGCACAAAAACTGACTCCTTTCAATACTTTTGTGTCTTTCCGCGATGGAAATTGGAAATGAACCTTT
TTAAATTCAATGTTTCCTTTTAAGTATTCAACCGTCTTTCCTTTTTCACTAAACGGATTAATTTTAGATT
TACGATCAATAACTTCATGAATACATTTTATTGCTCCTTTTGCTGATGCTATAGTTTCCAAGCTAGGAAT
TGTTAATGTAACAAATTGCGCTCCGTTAACCATGCAGAAGACAACAGTTACAATGGAAATTAAGTTGTAG
TTCCTCTGATCTTCAGGTAATTCAAATTCATCAATCATTAGCTGTATACCGTAGCAAAAAGCCGCACCAA
AACATGCAAATACCAGAAACCACATAAGGCCACTTTGAAGTCCAGTAAATAGACTCTTCTTAATTCCTGC
TGCTTCTGCTATCTTGAGATTCTCAGCATACCTCTGGGATTCTTTAGTTTCACCATTAAAAGCTTTAACT
GTTCTAATTGAATCGAATACTTGTTCTGCTATTGCTCCAGCTTTTGAGTAGCTCTGTAACTCTTTTTCCG
TTAAGCGTTTTGAGGATTTTCCAACATAAATGGAAAAACCAATAATTAGAATTGTGTAGACTGCTGTCAT
AGCAAGAGCTAATTTCCATCCTACAAGAAAAGCTATAACAACTTGCCAGATAAAAGCTGAGAAGAGGAAA
ACTGTTGTTACGAGCTTTTCACCAATAGCATCCTTTATCAAGTCAATATTGCTCGTTATATTGCTCGCAA
TATCCATTTCCGTCCTGGTGTCAAACCACGCCATATCCTGACTGAGGATAGATTGAATGAAAAGTTTACG
GAGTCGTGTAGTTTGTCTAAAAGCTAGAATATTTATCATGTAAACTGCATGCGTTAGGACAACAATGATT
GCTACAACAATCGCAACATTTAAGTAGAACATAGACATAGCATCAGCACTCATAGCTTCTGAAATTTCCA
TGCTGGTTGCATTTGTTAGAATTTTTCCACCTCCAAAGTAATTAAGTAGGTTGATTTTTGTCGTTGTTGT
ATTGGCTTTTGTACGATCAATTGTAATTCCAAGAACTTCAGCAAAACCAATCGCAGATAGACCAATTCCC
AAGGAAACCACAGACACAAGAAAGAGATTCACAACTGTAGCCAATTTTTCTGTAAACGTGGCATAGCGAA
ACTGAAATTTTATATTTAAAAATCTTTGTTGTAAAAAGAAAAATATTTAGAATTTATCAATATTAAAACA
CTCACTAAATGATGAAAATCAGAGGAAAAATTTAGAATTTTGGTCTTTTTCATTTTCTCTAAATTTTCAC
AAGTATCACAAGAAGTAAGATATTTTATAAGTTGAATTTCTTCAGTCAACAGCAGATCCACAACTGAATA
AACCTCGCAACTTGCAGAGAGAGCAAAATTTCTTAATTGGTTAATTTACATTTACACTTATAATAAAAAA
TTTTTGTGCAGTATGTTTAATCTTTTTCGTGTTAATATTTTTTACTAAAATCACGCACAGTTGATTCATT
TTACTTATTCATTCATACGTCATACGTGCATCTTCTTACGTGTTGAGCTTTTTTATAAATGAATATTTAA
AATTCTACTACGGTTTTCCTTAAAGATTTTCTTTTTATTTTTGTCAGATTCTAAT
>LLOTMP008312-Intron 2:PUTATIVE_protein_coding
GTAAATTATTTCGTTAATTACTCAATTATTCAGTATTTTAAACTGTTGTAACTTTCTTGAATCAG
>LLOTMP008312-Intron 3:PUTATIVE_protein_coding
GTAAGATTCATAATTTTAGGTAGACTAGGATTGTTGTCTGACTATCCTTTTTTACCAG
>LLOTMP008312-Intron 4:PUTATIVE_protein_coding
GTAAGTTTAAAGAAAACTGCTCAAAATCTTTAAGATCATTTTCTAAATTTGATAATTAAAG
>LLOTMP008312-Intron 5:PUTATIVE_protein_coding
GTAAAATATTTAAGAAGACTTCTCTTTTCTTTGTCTTGACTGAAAAAATATTTTTTCATTAG
>LLOTMP008312-Intron 6:PUTATIVE_protein_coding
GTATGAGTCAGATGAAGGATCAATCCTAAACTTTTCTAACATCTTTTTGGGTGATTCTTATTGCAG

>LLOTMP008312-Intron 7:PUTATIVE_protein_coding
GTGAGGAAGAATCTTTACGCATCCTTCTTCCTTCTTGTTCATTTTTTGTGATTCTTTTCAG
>LLOTMP008312-Intron 8:PUTATIVE_protein_coding
GTGAGTTGTGAACAAGAAGAGAATTTAGCAAAGATTTAACATTTTCTTATGAATTTCCAG
>LLOTMP008312-Intron 9:PUTATIVE_protein_coding
GTAAGAGGATCTTTTTTAATTTCTTATTTAAATTGTTCTTTTAATAGAATTAATTTGCTCCAG
>LLOTMP008312-Intron 10:PUTATIVE_protein_coding
GTAAGATTCTTAAAAATTAATTAATTAATTAATTTAAAAACTATTATTTTATTAATTCTTTTAG
>LLOTMP008312-Intron 11:PUTATIVE_protein_coding
GTAAGCCCATAAGCTAAATAATTCTTAAATTAGTAAATTAATCGACTTCCTTTTTCATTTTTAAG

L. longipalpis Glycoside Hydrolases from family 31
Glycosidase NET37
>LLOJ001847_1/ LlGlyMyo1
>LLOTMP001847_1-E1 exon:PUTATIVE_protein_coding
ATGTTCCCATCGATGATAAAGAAAAAGAAGAAATCAAAGGACTTCAAGATAAAGGCCTTTGTTGCTTGTT
TGTTCCTGATCATCGTCTTCCTCGTGGGATATGCGTACATTATGTACAACCAGCAGGTGCTTGCTCGTTC
CTACTTTGATCGCGTAAAATTGAACAAGGGCCAGCGTGTGATTGGAATCTACAACGAAAAGGGTGTTCTG
ATGGTAACTGGTGTTTGGGTACGACTATACATTCAGAGAAAGCCTACCACTGCCTCACGAATAATACGCT
GGAAGATGGAAGTGTGTGTCTGGAATGGGATGGCAAGGCGAGGATGTATCTCAATTTTCCAGGAACTCAG
TCCATCTGTTCCCTGCTACACGATCCGTTGGCAATCACTGTCTCCGGATGTTTTACCTACGGATTGCTAT
GAGTCTTTTATGGGTCAAGGACATTGGTATGGAGGTGGCCTAACACGTGGTGGGAACTGGCCTCTGGAGA
CGGAATCCTTTCCATTTGCACCCTTCATCACGGGTGATGCTAAACGACAGCAGTGGGGAAATGTCCTGAA
GAGATACTTTATAAGTTCACGCGGTGTGGCAATTCAGGTGGATGAGAAATCCCCTCTATATGTCAGCATG
AACGATAACAAATCAGGCGAGATGTGCTTCCGTGCGAAACATGATCATTTTGCCTTTGTCAATCGCCTTA
CGCCACTTCCTGAGCTGAAATACAGAATTTGCACGAGCGACAATATGCGTCAGTTGCATCAGCAGATGAC
ACAGCAGAGTCTCTGGGATGGACTGAAAGAGCATGATATCAATGTGGTTCATTCTCTTCTGGAGGAGCCA
GTTTGGCAAATTCCATCTTCTGGCGGGAAAGGTATCACGGGAGACAGCATCCACAACTACACTGAGCGCG
TAATCGCACTGGGATTCCTCCGTTTAGGCCATGTGCTGGTCAATGAATTCTGGCAGAGACACATTGGAGA
CTTTACGCTGGAAACTGAACGATTCCCAAATCTCGAGGAGACCGTTACAATTCTCCATCGTCGTGGCTTT
AGGATTGTCTTCTCAGTTCAGCCATACATAAGCACGGATAGTGATAATTTCGCTCAGTCTGTAGCGCAGA
AACTTCTAGTCTACGAGAGGCAATCTGAGAGAAGTATTCCTGCCCTGACGAGGTATAAAAGTGTTGCAAG
TGCAGGAGTATTGGATATCACAAATAATGCGTCAATTCCTTGGCTCATTGAAAAGCTGGAGAAGATTCAA
AAAACTTACAAAATTGATGGATTCTACTTGGATTTTGGAACTTCTCAGAACATGCCCCACTATTATCAGT
GCAATAAAACCCTCTACAATCCGGATCAATATAAAACAATCTTCACAACTGCCCTCGAGGGTGTTATCAG
TGTTATTGGAGTATCAAGTGCAGTAACTGTTCCTCGACCACCTGCCTTTGTTAGCCTACCTCCGGTAAAT
TCATCCTGGGAAGGACTTCGAACTGTCGTGACCAGTGCTCTGACATACGGCATCATTGGATATCCATTCA
TAATGCCA
>LLOTMP001847_1-E2 exon:PUTATIVE_protein_coding
GTCCAATTGGTGGAGATTATCTGCTCCCTCCTTCAGCTTCCAATGAAACTGTATCATTCTACTTCATGGA
AGAGCCTCCACTTCCGGATCAAGAACTCTACTTACGTTGGATGCAACTGGCAACGTTCCTACCTGTGATC
AGATTTACTCATTTACCGTCAGAGTATAAGAGTGAGCTGATAATGGAAGCAGTAAAGGAATTGACGCTTA
TTCGGCAGAAGGCAGTCATTCCACTGCTCAAGAAGTACCTCAGTGATGCCATGAATGAGGGTCTACCACT
CATTAGACCTCTCTGGATGTTGGATGCTCAGGACACGGCTTGTCTCTATGTCAATGACGAATTTTCAATT
GGTGAGGATCTCATTGTAGCACCTATCCTCGAGAAGGGACAATTACAGCGAGAAG
>LLOTMP001847_1-E3 exon:PUTATIVE_protein_coding
TTTATCTGCCTCAGGGTGTCTGGAAAGATGGGATTGACGGTTCCCTGCGCAAAGGAAGCCGCTGGATTCA
CAACTACCGCGTGCCTGAGGACAAAGTGGCTTATTTTATGAAAATGCCCGACAATACGAGATTCTAA
>LLOTMP001847_1-Intron 1:PUTATIVE_protein_coding
GTAAGTTTAATGAATCTTTCTTTTCATCATCAAACAACACAGTAAAAAATTCTTAATTTATAG
>LLOTMP001847_1-Intron 2:PUTATIVE_protein_coding
GTAAGCAATATATCAAGTTCTGTAACAGACACATACTAAAATAACTTCTTTTCACACATTCCTTAAAATC
TAATTCATGTTAATTAAGAGCATGATTAACAATCAATCGAGTAATTAAAATATTCGCAGAATATGAATAA
TTTAGTCTTGCATTAATTTTGAGATCTTTTCAAGATCAAATGGTAGCTTCCTCTCAATTTTTCAAGAACT
CATGAATATCAATATAATCTAATTTGTATGTGACATGAGAAGACACTCTGCTGATTAATACGCTTGTTTT
CTTTGCAG

>LLOJ001847_2/ LlGlyMyo2
>LLOTMP001847_2-E1 exon:PUTATIVE_protein_coding
ATGATAAAGAAAAAGAAGAAATCAAAGGACTTCAAGATAAAGGCCTTTGTTGCTTGCTTGTTCCTGATCA
TCGTCTTCCTCGTGGGATATGCGTACATTATGTACAACCAGCAGGTGCTTGCTCGTTCCTACTTTGATCG

CGTAAAATTGAACAAGGGCCAGCGTGTGATTGGAATCTACAACGAAAAGGGTGTTCTGATGGTAACTGGT
CGTTTGGGTACGACTATACATTCAGAGAAAGCCTACCACTGCCTCACGAATAATACGCTGGAAGATGGAA
GTGTGTGTCTGGAATGGGATGGCAAGGCGAGGATGTATCTCAATTTCCAGGAACTCAGTCCATCTGTTCC
CTGCTACACGATCCGTTGGCAATCACTGTCTCCGGATGTTTTACCTACGGATTGCTATGAGTCTTTTATG
GGTCAAGGACATTGGTATGGAGGTGGCCTAACACGTGGTGGGAACTGGCCTCTGGAGACGGAATCCTTTC
CATTTGCACCCTTCATCACGGGTGATGCTAAACGACAGCAGTGGGGAAATGTCCTGAAGAGATACTTTAT
AAGTTCACGCGGTGTGGCAATTCAGGTGGATGAGAAATCCCCTCTATATGTCAGCATGAACGATAACAAA
TCAGGCGAGATGTGCTTCCGTGCGAAACATGATCATTTTGCCTTTGTCAATCGCCTTACGCCACTTCCTG
AGCTGAAATACAGAATTTGCACGAGCGACAATATGCGTCAGTTGCATCAGCAGATGACACAGCAGAGTCT
CTGGGATGGACTGAAAGAGCATGATATCAATGTGGTTCATTCTCTTCTGGAGGAGCCAGTTTGGCAAATT
CCATCTTCTGGCGGGAAAGGTATCACGGGAGACAGCATCCACAACTACACTGAGCGCGTAATCGCACTGG
GATTCCTCCGTTTAGGCCATGTGCTGGTCAATGAATTCTGGCAGAGACACATTGGAGACTTTACGCTGGA
AACTGAACGATTCCCAAATCTCGAGGAGACCGTTACAATTCTCCATCGTCGTGGCTTTAGGATTGTCTTC
TCAGTTCAGCCATACATAAGCACGGATAGTGATAATTTCGCTCAGTCTGTAGCGCAGAAACTTCTAGTCT
ACGAGAGGCAATCTGAGAGAAGTATTCCTGCCCTGACGAGGTATAAAAGTGTTGCAAGTGCAGGAGTATT
GGATATCACAAATAATGCGTCAATTCCTTGGCTCATTGAAAAGCTGGAGAAGATTCAAAAAACTTACAAA
ATTGATGGATTCTACTTGGATTTTGGAACTTCTCAGAACATGCCCCACTATTATCAGTGCAATAAAACCC
TCTACAATCCGGATCAATATAAAACAATCTTCACAACTGCCCTCGAGGGTGTTATTAGTGTTATTGGAGT
ATCAAGTGCAGTAACTGTTCCTCGACCACCTGCCTTTGTTAGCCTACCTCCGGTAAATTCATCCTGGGAA
GGACTTCGAACTGTCGTGACCAGTGCTCTGACATACGGCATCATTGGATATCCATTCATAATGCCAG
>LLOTMP001847_2-E2 exon:PUTATIVE_protein_coding
GTCCAATTGGTGGAGATTATCTGCTCCCTCCTTCAGCTTCCAATGAAACTGTATCATTCTACTTCATGGA
AGAGCCTCCACTTCCAGATCAAGAACTATACTTACGTTGGATGCAACTGGCAACGTTCCTACCTGTGATC
AGATTTACTCATTTACCGTCAGAGTACAAGAGTGAGCTGATAATGGAAGCAGTAAAGGAATTGACACTTA
TTCGGCAGAAGGCAGTCATTCCACTGCTCAAGAAGTACCTCAGTGATGCCATGAATGAGGGTCTACCACT
CATTAGACCTCTCTGGATGTTGGATGCTCAGGACACGGGCTTGCTCTATGTCAATGACGTAATTTTCAAT
TTGGTAGAGGATCTCATTGTAGCACCTATCCTCGAGAAGGGACAATTACAGCGAGAAG
>LLOTMP001847_2-E3 exon:PUTATIVE_protein_coding
TTTATCTGCCTCAGGGTGTCTGGAAAGATGGGATTGACGGTTCCCTGCGCAAAGGAAGCCGCTGGATTAC
AACTACCGCGTGCCTGAGGACAAAGTGGCTTATTTTATGAAAATGCCCGACAATACGAGATTCTAA
>LLOTMP001847_2-Intron 1:PUTATIVE_protein_coding
GTAAGTTTAATGAATCTTTCTTTTCATCATCAAACAACACAGTAAAAAATTCTTAATTTATAG
>LLOTMP001847_2-Intron 2:PUTATIVE_protein_coding
GTAAGCAATATATCAAGTTCTGTAACAGACACATACTAAAATAACTTCTTTTCACACATTCCTTAAAATC
TAATTCATGTTAATTAAGAGCATGATTAACAATCAATCGAGTAATTAAAATATTCGCAGAATATGAATAA
TTTAGTCTTGCATTAATTTTGAGATCTTTTCAAGATCAAATGGTAGCTTCCTCTCAATTTTTCAAGAACT
CATGAATATCAATATAATCTAATTTGTATGTGACATGAGAAGACACTCTGCTGATTAATACGCTTGTTTT
CTTTGCAG

>LLOTMP001881/ LlGlyMyo3
>LLOTMP001881-E1 exon:PUTATIVE_protein_coding
XXXXXXXXXXXXXXXXXXXXXXXXXXXXXXXXXXXXXXXXXXXXXXXXXXXXXXXXXXXXXXXXXXXXXX
XXXXXXXXXXXXXXXXXXXXXXXXXXXXXXXXXXXXXXXXXXXXXXXXXXXXXXXXXXXXXXXXXXXXXX
XXXXXXXXXXXXXXXXXXXXXXXXXXXXXXXXXXXXXXXXXXXXXXXXXXXXXXXXXXXXXXXXXXXXXX
XXXXXXXXXXXXXXXXXXXXXXXXXXXXXXXXXXXXXXXXXXXXXXXXXXXXXXXXXXXXXXXXXXXXXX
XXXXXXXXXXXXXXXXXXXXXXXXXXXXXXXXXXXXXXXXXXXXXXXXXXXXXXXXXXXXXXXXXXXXXX
XXXXXXXXXXXXXXXXXXXXXXXXXXXXXXXXXXXXXXXXXXXXXXGGTCCACAGCAAAAGTATCAGTAC
TGGCCAGTTGAGAAACTCGTCCTATCCAATTATTCCTACGTTACAAAGAAGCCGACAGTGCGGCTATTGC
TGAGAGATATTGGCTCAATTCGGAAGGAGCTTTTATTTATCTCTCAGACCGAGCTCCACTCTTCATTGAC
CAAAACAGCGTGGTCCACAGCAAAAGTATCAGTACTGGCCAGTTGAGAAACTCGTCCTATCCAATTATTC
CTACGTTACAAAGAAGCCGACAGTGCGGCTATTGCTGAGAGATATTGGCTCAATTCGGAAGGAGCTTTTA
TTTATCTCTCAGACCGAGCTCCACTCTTCATTGACCAAAACAGCATGATGGACAATCATTTGTGCTTCGT
TGCTAGCCTTGCACTGCCCTACAATCCACGTGCTCAGACGTATGATTTTGAGTACAGCATCGGGGTGGCT
TCTGATCCGAGAAAAGCTCACATGGAGGCTGTTCATCGGTTTCTGGGAAAGCCCTCAGGGGTGCCTGATA
GACTAATGGCAGCACGACCTATTTGGTCAACATGGGCCAGGTACAAGAGGGACGTTGATCAATATGTAGT
GATGGAGTTTGCAAAAGAAATCATCGACAACCGCTTCACAAATAGTCAATTTGAGCTTGATGATGATTGG
GAAATTTGCTACGGAGCTTTGACTTTTAGCACTACAAAATTCCCCAATATTCGCAAAACCGTTCAAGATC
TAAAGTCAATGGGATTTAGGGTAACGCTGTGGATTCATCCGTTCATTAACAAGGGCTGCAACCCATGGTA
CAATGAAGCTCTCACAAATAG
>LLOTMP001881-E2 exon:PUTATIVE_protein_coding

ATACTTTGTTCTCGATCACAATGGCAAAGAGGATACTCAGTGGTGGAATAGCGAACAAGGTGAGGCTGCG
TACATTGATTTTACAAATCCTGAAGCTGCAAATTGGTACACATCGAGATTGCATAAGCTCCTCGAAGACA
CAGGCATTGATAGCTTTAAGTTTGATGCTGGTGAAAGTAGTTGGCAACCTGAT
>LLOTMP001881-E3 exon:PUTATIVE_protein_coding
GATCCAGTACTACCCTCTACTTCCCTGGATCAGCATCCATTGAAAATTCAAACAGACTACATAATGCATG
TTGCTCAATTTGGCCCAATTGTGGAAGTTCGATCGGGTTTCCGTAATCAATTCCAGCAGATTTATATGCG
AATGATTGACAAGGATTCCCTTTGGAGCTGGAACAATGGTCTCCCAACTTTGATCACGACGCTCCTTCAG
CTCAATATGAATGGATATCCTTTGGTTTTGCCCGACATGATTGGAGGCAACGGCTACGAGGGAGCACCTT
CGAGGGAAATGTTTATTCGCTGGCTTCAAGCTAATGTTTTCATGCCCAGCCTTCAGTTCTCATACGTTCC
ATGGGACTACTCGGATGGGGATGGCATTAAGATCCTCGATCTATGTAGGCATTTTGTTAAACTGCACGAA
GACTACACTGACTTGATCATGGAACGTTTCCAAAAAGCCGTAGACCATGGGGAGCCTGTTAATCCACCCA
TTTGGTGGGTTGATCCTAAGAATCCAGTAGCTCAAAGTATCTACGAT
>LLOTMP001881-E4 exon:PUTATIVE_protein_coding
XXXXXXXXXXXXXXXXXXXXXXXXXXXXXXXXXXXXXXXXXXXXXXXXXXXXXXXXXXXXXXXXXXXXXX
XXXXXXXXXXXXXXXXXXXXXXXXXXXXXXXXXXXXXXXXXXXXXXXXXXXXXXXXXXXXXXXXXXXXXX
XXXXXXXXXXXXXXXXXXXXXXXXXXXXXXXXXXXXXXXXXXXXXXXXX
>LLOTMP001881-Intron 1:PUTATIVE_protein_coding
GTAATTTTTTGAAGGAGCTTTGCAGCAAAATCAGAATGTTTAAATTCAATTCTTCTCATTCAG
>LLOTMP001881-Intron 2:PUTATIVE_protein_coding
GTGAGTAGTTTAGCTAATAACATTAAGGATCTCGTTAACAGGGTTTACTGCATTGTAG
>>LLOTMP001881-Intron 3:PUTATIVE_protein_coding
XXXXXXXXXXXXXXXXXXXXXXXXXXXXXXXXXXXXXXXXXXXXXXXXXXXXXXXXXXXXXXXXXXXXXX
XXXXXXXXXXXXXXXXXXXXXXXXXXXXXX

>LLOTMP000840/ LlGlyMyo4
>LLOTMP000840-E1 exon:PUTATIVE_protein_coding
ATGGAAAAGAAGCGCGTAAAGGGAAACCCAAAGTATAAGTCCGCTTTTAAGTGGGGTACAGCTGGAATTA
TAGCCACTGTGATTTTCGTTCTTGTGGAGGTTTTTATAATTCACTGGGTGACACCATTTGATAATTTCCT
TAGGGTTCACTTCCCTAAAGCCGGAGTTCATGTGCAATTTGAGTGTGAACACATAGAGGATGGGGTAGCA
TTTAGTATTCACAAGAATCGCACCCTTGTTCAAATTGTCCGGATGGGCGACAATTTAGGATGGGATTTGA
GGGTTGAGGATCACGGAGGTGGGAAGTATGTTTTAAGGAGTCGCGAGGGATCAGTGAGCTTTAATACGGC
TGTTGATGAAGATGGTTTGGCTGTTTTTCACATCAATCAAACAATTCGTACAACGGATTTCGTTGAACAT
TGCTTTGATCTGGTGAATCCTGATGGGAATAATTGGTTTGCTGGTCCGCATAAATACTACCAGCATTGGC
CATCACAGGTTCTACAATTCACAGATGATGCCTACTTGCCGAAAGAGTCTTCACATTCATCCATATCCGA
GAGGTATTGGCTCACATCCAAAGGGATGTTCATCTACTTCAGTGACCGAACTCCACTATTCCTCAATCGC
AAACCCAACCATTTGTGCTTTGCTGCAAAGAAGCAAAATCCTTACTACACCTACGACACAGTTTTTGTCT
TTAATTATACAATTGGAATAGCTGCAGATGCCAGGAAAGCTCACAAGGCTGCAGTGGGTAGGTATTTGAG
GAAACCCCTTCATTATCCTGACAGGAGAGTAATTCAGCATCCAAAATGGATCCTCCGAAATCACAACATG
TCTCAAGTTCAGGAATATATCGACAATTTGCTGTACTACAATTTTAGCACATCCAGCATCATTCTGGATA
AATCATGGGAAACGTGCACAGGAGCTTTGGAGTTTGATCCGATCAAATTTCCCAATGTTGGGAGTATGAT
ACGATATTTTAGACGCCACAGAACCCATCTCACACTCACTGTGACTCCGTACATTCACGAGGATTGCAAC
CCCTACTTCCAGGATGCTCTCTCACGTGGATTTTTGGTCAAGACTCACCAGAATACCTATTTAAATTTCG
ATCGAACTGCTACTGTGGACTTCACACAGGTTGCTGCTAGACGGTGGTTTCTGGCACGACTCAAGGATCT
GCAAGCTCTAGGCGTTGACAATTTCTACTTTGAAGGTGGAGAGTTCGATTCAGCGCCAAATGATCCGAAT
TTCGAAGGAGTCTCAACGAGTTTGCATCCCATTCAGTACACCATTGATTCCCTAAGAGCATTGGCGGAGT
TTGATCACAACACAATTGTTCGAACGGGATTTTGCACCCAGGATCTTCCTCTTTTGCTCCGACTTCCGGA
TATGGATAATCGATGGGACATTCAAAATGGTTTAGATTCGCTTATTCCGAAAATATTGCAGACAAACCTC
AATGGATACTACTTCCTCATGGCTCCAATCGGAGGAACTACTTCAGAGGGTCTGACAAAGGAGCTCTACA
TACGCTGGATGCAAGCTGTGGTCTTCTTGCCATCAATGGCCTTTTCGACTCCTCCATGGGAGTTTGATGA
TGAAACCATTGAATTAGCTCAAAAATTCATTCGTCTTCATATGCGACATGTATCCTTATTTGTGGATCTG
TTTAAGTTGGCAAAATCGGATGGGGATCCTGTTAATTTACCTATTTGGTGGCTCTACCCGCAAAATTGGC
GTGCACAGGAAACTCATGATC
>LLOTMP000840-E2 exon:PUTATIVE_protein_coding
AGTATCTCCTGGGTGAGGACATTATCGTGGCTCCGGTGCTGAAGCCAAATTCTTTGGAAAGAGGAATATT
CCTCCCATATGGCATATGGCGCGATGGCAATGATAACAGTTCACTCTACCAGGGACCTCGGTGGTTACCT
AGCTATAGAGCCCCATTGGATGTCCTCCCATACTTTGTCCGAGCTCGCAATAAAAATTAA
>LLOTMP000840-Intron 1:PUTATIVE_protein_coding
GTAAGTAAAAGAAAGAAATATTAACGAACGAATTGTCCTTCAATTTACTTGTCTGTTTGTATCCTTGTTT
GTGTGGATCTCTGAAAGATGTTATGAATGCATGAAAATGAATAAAGTCCTTTACAGGAATCTGTTGAACA
ATAAATCTAATAATTAAAACCAAGTTTATTTGTTGAATTTAAATTAAATTAATTTATTGAAACAAGGATC
TAATATAATTTGTTTTGCTACACATGGTTTTGCGGATTAACATGGCTACAGAGGAATCATGGATATCTCT

TCTAGATAGATGTCCCTAATACATGATGACCTTTGGTTTGATTTCGTTTCTTATTTTTCAAGGCCAAGAA
TTCTTAATTTTTTTTTTCGTAGCAAATCCGCAATACATTTTTTTTCTAATTAATTTTAATAATTTTTTTT
GTCTTTTTTTCGTAG

Lysosomal alpha-glucosidase
>LLOTMP006451/LlLysAglu1
>LLOTMP006451-E1 exon:PUTATIVE_protein_coding
XXXXXXXXXXXXXXXXXXXXXXXXXXXXXXXXXXXXXXXXXXXXXXXXXXXXXXXXXXXXXXXXXXXXXX
XXXXXXXXXXXXXXXXXXXXXXXXXXXXXXXXXXXXXXXXXXXXXXXXXXXXXXXXXXXXXXXXXXXXXX
XXXXXXXXXXXXXXXXXXXXXXXXXXXXXXXXXXXXXXXXXXXXXXXXXXXXXXXXXXXXXXXXXXXXXX
XXXXXXXXXXXXXXXXXXXXXXXXXXXXXXXXXXXXXXXXXXXXXXXXXXXXXXXXXXXXXXXXXXXXXX
XXXXXXXXXXXXXXXXXXXXXXXXXXXXXXXXXXXXXXXXXXXXXXXXXXXXXXXXXXXXXXXXXXXXXX
XXXXXXXXXXXXXXXXXXXXXXXXXXXXXXXXXXXXXXXXXXXXXXXXXXXXXXXXXXXXXXXXXXXXXX
XXXXXXXXXXXXXXXXXXXXXXXXXXXXXXXXXXXXXXXXXXXXXXXXXXXXXXXXXXXXXXXXXXXXXX
XXXXXXXXXXXXXXXXXXXXXXXXXXXXXXXXXXXXXXXXXXXXXXXXXXXXXXXXXXXXXXXXXXXXXX
XXXXXXXXXXXXXXXXXXXXXXXXXXXXXXXXXXXXXXXXXXXXXXXXXXXXXXXXXXXXXXXXXXXXXX
XXXXXXXXXXXXXXXXXXXXXXXXXXXXXXXXXXXXXXXXXXXXXXXXXXXXXXXXXXXXXXXXXXXXXX
XXXXXXXXXXXXXXXXXXXXXXXXXXXXXXXXXXXXXXXXXXXXXXXXXXXXXXXXXXXXXXXXXXXXXX
XXXXXXXXXXXXXXXXXXXXXXXXXXXXXXXXXXXXXXXXXXXXXXXXXXXXXXXXXXXXXXXAATGTT
TTCAAGTTTGCATGTTTTAATTTTGATTTCCGTATTTCCATCATTTTAGATGCTACAACCGAAATGATGC
TACAACCGAAATGTTCCATGGAATTTATATAAACGCACCGTATCCAGTTGAAGTTGAAATCACTGAGAGC
TATTTAGTTATAGTTAGAGCTATTTTTGATACATCATTTCACATTGATGTACTATGTGGTCCAACAATCG
GTAATTTGATGGATCAGTTAATGGAGCAAACAGAACAAAATCTACATCCACTGCCAAATTGGTTTTTTGG
TTTTCACATTTGTGATACGAATATTACAAGAAATCTCACAGATTCAGTGATTGAGGTTCTGGAGTTACTT
TCCTCTGATTTG
>LLOTMP006451-E2 exon:PUTATIVE_protein_coding
CCATTTGATACTCATTGCATACGAGAACAGTTGCTGTGGTTGGGTGACGATATAAAGGGATTACCAAGCG
AG
>LLOTMP006451-E3 exon:PUTATIVE_protein_coding
TACTTCCAGTTGATCTCAAGAGTGCAGCTTATCATATGGCCAAAGCAAAAGGGATTATTAATGAGGCATC
CCACTTTAGAAGAGCCTTACATTGGCCGTATTGGCAAGACGAATGTGGTCTATGTGGACTGGGTTGGATC
AAATCAAGAAAATTTGTATGAATGGTCAAATCAATTCATGCCCAACAATTTATCTGCAGATGGATATATG
GTGCAGGCCAATTGGATGTGGGATCAAAGTGATGGTGAAAAAATACCCCACTCATTTCCATATATTTCAA
TGGATATGATAAATGCCAGCAAGAATATAGTACCGTGGTTTATTCGTCAAAGTGGCACAGATGGAAAGGC
CTCAATTTTCTCCCATAATTTTGCAGCATCAGCGCAGATTGACGTAATAAGGAAAGTCCTTGGAAATGAA
TCGTTTATTTTATCTGAAAGTTCGCTATTTGCAAATGTACCAATCTCTCGTAGACAAGTACCATCTACAT
GGACAGAATTGAGAAATTTA
>LLOTMP006451-E4 exon:PUTATIVE_protein_coding
GTTCGTCGTACGATGGGCCAATCGATTTCTGGAATTTACTTCAGTTCAGCAAATATTTGTGGAGATGGCG
AGACATGGACGGAAGAGCTATGTATTCGTTGGTATCAATTCGCTTCGCTATCACCTTTCTACCGAGTAGC
GTCCGCAAAGGCTCCAACATCATTTTCGAAATTCGCTCAAAGTTTGCTGAGAGCATCAACGGAGAGACGA
TACTCACTTTATTTGTACATTCATACTGTTGTGACAGAAAGAAGACCTCTTCTCACACCTTTATTCTATG
AATATCCTGACCTCATAGCATTGATGGACAATTTAACACACCAGGCGCTTGTCGGACCCTCTCTCCTGTT
TGCACCAGTACTGCTGCCTGGTGTTCAACATATCAATATTTTCTTCCCTGAGGTATACTACGAGATTGGC
GGTGGGCAGCAATTAATTGCAAATACGTGGGCTGAACTACCTGTTGTTGAGACAGACGCACCCCTTTTCA
TTCGTGCTGGACATATTGTTCCTATACAGCATACAAAG
>LLOTMP006451-E5 exon:PUTATIVE_protein_coding
AATGTTCGGTCAATAACAGTCATGCAAATGAAACCTATTCATTTATATGTCGCTCTGGGTCCATTGGAAA
ATGGCATAAGTACTGCAATAGGTAAAGTGCAATTTGATTCAGAATACAAAGTTACATTTAACGCGGCTTA
CACAACCTCATCAGAACAG
>LLOTMP006451-E6 exon:PUTATIVE_protein_coding
ACGCAACTAAAATTATCATCGTACTCTAAACTAACTTGTCGCACCACTACAAATCTCACCATAGAAAGT
>LLOTMP006451-E7 exon:PUTATIVE_protein_coding
CGATGCCGTGTTGAGGAGGGATTTCTCCCGGCAATTTTCGAGAAGTTTCAACCCCTCTCCTACXXXXXXX
XXXXXXXXXXXXXXXXXXXXXXXXXXXXXXXXXXXXXXXXXXXXXXXXXXXXXXXXXXXXXXXXXXXXXX
XXXXXXXXXXXXXXXX
>LLOTMP006451:LLOTMP006451-RA intron 1:PUTATIVE_protein_coding
GTAACTGAGCAG
>LLOTMP006451:LLOTMP006451-RA intron 2:PUTATIVE_protein_coding
GTGAGGAGCGCTATTAGTAAAATGAAATGGCTCGGAAAAAGTTTATTGCTCCCATGACTGCAG
>LLOTMP006451:LLOTMP006451-RA intron 3:PUTATIVE_protein_coding

GTAAGTATCTATAGAAAATATATTAGACTTATGTACCTATAACGCATATCTTTTTTACGTAAATATTTTG
AAATGTGCCGTTTTATAATATTAATTTCTAGTTTTTTTTTTTAAAGAAAATATTATATGTATTTAGGTTT
ACAGGTATATGAATGTACTTCACATGAATAGGCTTAGATGTAAAAGTTCATTTAAAAATACACAGCTAAA
TCCTTTTAGCTGTGATTCTTTTTATATTGTTGTGATGCCTGTGATGCCCAGTAAAGTTGGTTGTTTTAAG
TAAGAAAATCTTTGATTCAG
>LLOTMP006451:LLOTMP006451-RA intron 4:PUTATIVE_protein_coding
GTATTATTTGCTGCTGTATTATGTATAGAAACATTAACAAATTTCTATACATCATTTTATATTGGAATTT
CATATTGAATTGTATTGAATGTTAAATTTCATTTTAG
>LLOTMP006451:LLOTMP006451-RA intron 5:PUTATIVE_protein_coding
GTAAATTTATATTTTTGTAAAGATATTTTATCAATCCAATATATATAATATATATATTTAATTTCAATTA
G
>LLOTMP006451:LLOTMP006451-RA intron 6:PUTATIVE_protein_coding
GTAAAAATCTATGGTTCTGGTGTTTCGGATGTTGGCGTCTTTACATATTCTATAAAAGTGGGGCATAATT
TATGTGGTGGAAATTTAAACTTGACAGAAGATTTGAGTTTTCTAAATAAAAGAAATTCAATATAAGGATT
ACTGATTTTTTCTTTATGTATTAGCAATAGTATTTGTCACACGAAAAATTTATTGTATTGTACCTACTGT
ATACAGATTGTTTGTAATAAATAAAAAAAATGTATTAGTTTTCTCAATGTACATTGTATATATTCGCTTG
GCCCATTCATTATGAACCTATCTCAAAAACGCTACTTTTCAGGAGAGCCGATTTTCTAAAATGTATGAGA
AATTTACGTATTTTGCCTTTTTTCTGGCTCCAACTTACCAATTATCTCACACAATGATGGTATTCACTCA
CTATTACCATTTAGAAAGCACTGGCACCCCAAATTATGTAAAATAATTAGCAAAAAGTGCAAAAATATTG
CAAAATATGCACAGAAAATACTTTATAGGTTCTCCTTGCACGGAGTTTGAGAAAATTTTTATGTGCCGGA
GTGTTCGGCAAAGCTCGGGTCGACCGTCCTTGAATGTAAGCACTAGGTCTCTCTTTCTTTCCTGCGTGCG
CCGATAGATTCGTGTGCATCTTGTATGTGTGTGTGTTAGCTCCACAATTTCCTTCAGCGAGAATGAATTT
TTCGTCCCAAATGGAATTGTTGCTTCTCTGCCACCAAGGGGATTTGGAATCTCATCTCCATAACCCAATG
GATTATCACATCCTGGACAATTAGGGGGGGCTGGGGCAAAAATGTTTGTTTTTTATCAAATTCTGGAAAA
ATAGGGGATTTTGGGTCAAATTGGTGGGTTTTTCGCCGGAATTGTGGTCACTCCTCATTGGTAATGATGG
AGTGACCTCCGGATTGGGGATCTTCATCTCAGGAATATCAACTCCTGGACAATTAGGGGGGCTGGGGCAA
AATTTTTTATTTTTAGCCGGAATTGTGGTCACTCCTCATTGGTAATGATGGAGTGACCTTCGGATTGGGG
ATCTTCATCTCAGGAATATCAACTCCTGGACAATTAGGGGAGGCTGGGGCAATTTTTTATTTTCGATGCA
ATTGTGGTCACACCTCATTGGTAATGATGGAGTGACCTTCGGATTAGGGATCTCATCCCAGGAATATCAA
CTCCTGGACAATTAGGGGAGGCTGGGGCAAATTTTTTGTTTTTTGCCGGATTTGTGGTCACTCCTCATTG
GTAATGATGGAGTGACCTCCGGATTGGGGATCTCACCCCAGGAATATAAAATTCTGGAAAAATAGGGGAT
GTTGGGGCAAATTGGTGGGTTTTGCGCCGGAATTGTGCTCACTCCTCAGTGTTAATGATGACTAAAATAT
TTTATAAAAATTTTTTCAATAAACAAGAATATTTTCAAGCGGCTTCCTCAGAAAATCGCACTGCCTATAT
ATAACTTTTCTTAATTAAAAAAAAATATTGTAAAGCCCGTAAAGTTTTCGAGCAATACCAAACTTTTTAT
TTAAAATTTTTCTTCAATATAATAAAAAAATCGTCAAGCCCGTAGAGCCTTCGCAAAGAATCAAACTACTT
TTCTTTTCTATTGAGAAGAAATACCTTTTCTTCTCAATAAATTCACTCATTATTCTTAAAGAGTTCTTTC
CTTTTTCCTTTAAGACTCCCGAAAATTCTTTCAAAGAACAAAATAATTCTTAATTAAAGAAAGAATTAAA
AAAAGAAATGCCCTTTCTTCTCAATAAATTCACTAGTTATTCTTGAAGAGTTCTTTCCTTTTTCCTTTAA
ATTCCCGGAAATTCTTTCAAAGAACAAAATGATTTTTTATTAAAGAAAGAATTAAAGGAAGAAATACCTT
TTCTTCTCAATAAATTCACTAGTTATTCTTGAAGACTTCTTTCCTTTTTCCTTTAAATTCCCGGAAATTC
TTTCAAAGAACAAAATGATTTTTTGTTAAAGAAGGAATTAAAGGAAGAAATAGCTTTTCTTCTAAATAAA
TTCATTAGTTATTCTTGAAGAGTTCTTTCCTTTTTCCTTTAAATTCCCGGAAATTCTTTCAAAGAACAAA
ATGATTCTTAATTAAAGAAGGAATTAAAAAAAGAAAGGCCTTTTCTTCTAAATAAATTCACTAATTATTC
TTGAAGAGTTCTTTCCTTCAGGATTCCCGAAAATTCTTTAAAAGGACAAAATGATTCCTAATTAAAGAAG
GAATTAAAGAAAGAAAGGCCCTTCTCAATAAATTCACTAATTATTCTTGAGGAGTTCTTTCCTTTTTCCT
TCAAGATTCTTTATTAAAGAAAGAAATGCCTTTTTCTTAACAAATTCAGTAATTATTCTTAAATAGTTCT
TTTTTTTCATTTTTTTTAAATACCCCAATTTTTTTACAAGAACAAAATGATTCAAAAAAATTTGGATCT
AAAAAAAATGGTGAATTTTCTAAGAAGAGAAGGCATTTCTTTCTTTAATCCCTTCTTTTATAAAGAATCA
TTTTGTTCTTCTAATTTTTCTTTATTTTCCTATTAAAATTAAAATGGTTTCGTTCTTCTAAAGACTTTTC
AGATACCTATTTTGATAAAAATTAAAAGGGAAGGATCTCTTTAAGAATAATTAGTGAATTTTTTGAGAAA
AAAGGGAATTTCTTTCTTTAGTTCCGTATTACAGATAAAACTACTTTTTTCTTCTTAAAATTTTTAAGGC
ACTTTGATAAAATTTAAAAGAAAATAACTCTTTAGGAATAATTAGTGAATTTGTTGACCAAAAAAGTAGA
TTTCATTCTTTAAATCCTTCTTTGATTAAAAATTATTTTTATTCAGGCTTGAGAATATTTTTCTATTAAA
ATGTAGTATAGTTATTACCGAAGGCTCTACGGGCTTTACAATATATATTTTTTTTTAAATTAAGAGAAGTT
ATAAATAGGCAGTTTGACTTTCTGCGGAAGCTGCTTGTAAATATTCTTTTTATTGAAAATAATTTTTTTA
AAATATTTTAGTCACTTCATCATTGTAAATAAAAAGTGACCACAATTTCGGTGCAAAACCCTACAATTTG
CCCCAACATCCCCTATTTTTCCAGAATTTTATATTCCTGGGATGAGATXXXXXXXXXXXXXXXXXXXXXX
XXXXXXXXXXXXXXXXXXXXXXXXXXXXTTTAAACTTGACAGAAGATTTTGAGTTTTCTAAATAAAAGAA
ATTCAATATAAGGATTACTGATTTTTTCTTTATGTATTAGCAATAGTATTTGTCACACGAAAAATTTATT
GTATTGTACCTACTGTATACAGATTGTTTGTAATAAATAAAAAAAATGTATTAGTTTTCTCAATGTACAT
TGTATATATGTATCGACGCTCCCGAAGATTATGTGTTAAAAGATAGGAATATGGTTCATAATCTTCGGGC

ACGTCGGTATTTTTTTACAATGTACATACATATCGAGGGCTGCATTGTXXXXXXXXXXXXXXXXXXXXXX
XXXXXXXXXXXXXXXXXXXXXXXXXXXXXXXXXXXXXXXXXXXXXXXXXXXXXXXXXXXXXXXXXXXXXX
XXXXXXXXXXXXXXXXXXXXXXXXXXXXXXXXXXXXXXXXXXXXXXXXXXXXXXXXXXXXXXXXXXXXXX
XXXXXXXXXXXXXXXXXXXXXXXXXXXXXXXXXXXXXXXXXXXXXXXXXXXXXXXXXXXXXXXXXXXXXX
XXXXXXXXXXXXXXXXXXXXXXXXXXXXXXXXXXXXXXXXXXXXXXXXXXXXXXXXXXXXXXXXXXXXXX
XXXXXXXXXXXXXXXXXXXXXXXXXXXXXXXXXXXXXXXXXXXXXXXXXXXXXXXXXXXXXXXXXXXXXX
XXXXXXXXXXXXXXXXXXXXXXXXXXXXXXXXXXXXXXXXXXXXXXXXXXXXXXXXXXXXXXXXXXXXXX
XXXXXXXXXXXXXXXXXXXXXXXXXXXXXXXXXXXXXXXXXXXXXXXXXXXXXXXXXXXXXXXXXXXXXX
XXXXXXXXXXXXXXXXXXXXXXXXXXXXXXXXXXXXXXXXXXXXXXXXXXXXXXXXXXXXXXXXXXXXXX
XXXXXXXXXXXXXXXXXXXXXXXXXXXXXXXXXXXXXXXXXXXXXXXXXXXXXXXXXXXXXXXXXXXXXX
XXXXXXXXXXXXXXXXXXXXXXXXXXXXXXXXXXXXXXXXXXXXXXXXXXXXXXXXXXXXXXXXXXXXXX
XXXXXXXXXXXXXXXXXXXXXXXXXXXXXXXXXXXXXXXXXXXXXXXXXXXXXXXXXXXXXXXXXXXXXX
XXXXXXXXXXXXXXXXXXXXXXXXXXXXXXXXXXXXXXXXXXXXXXXXXXXXXXXXXXXXXXXXXXXXXX
XXXXXXXXXXXXXXXXXXXXXXXXXXXXXXXXXXXXXXXXXXXXXXXXXXXXXXXXXXXXXXXXXXXXXX
XXXXXXXXXXXXXXXXXXXXXXXXXXXXXXXXXXXXXXXXXXXXXXXXXXXXXXXXXXXXXXXXXXXXXX
XXXXXXXXXXXXXXXXXXXXXXXXXXXXXXXXXXXXXXXXXXXXXXXXXXXXXXXXXXXXXXXXXXXXXX
XXXXXXXXXXXXXXXXXXXXXXXXXXXXXXXXXXXXXXXXXXXXXXXXXXXXXXXXXXXXXXXXXXXXXX
XXXXXXXXXXXXXXXXXXXXXXXXXXXXXXXXXXXXXXXXXXXXXXXXXXXXXXXXXXXXXXXXXXXXXX
XXXXXXXXXXXXXXXXXXXXXXXXXXXXXXXXXXXXXXXXXXXXXXXXXXXXXXXXXXXXXXXXXXXXXX
XXXXXXXXXXXXXXXXXXXXXXXXXXXXXXXXXXXXXXXXXXXXXXXXXXXXXXXXXXXXXXXXXXXXXX
XXXXXXXXXXXXXXXXXXXXXXXXXXXXXXXXXXXXXXXXXXXXXXXXXXXXXXXXXXXXXXXXXXXXXX
XXXXXXXXXXXXXXXXXXXXXXXXXXXXXXXXXXXXXXXXXXXXXXXXXXXXXXXXXXXXXXXXXXXXXX
XXXXXXXXXXXXXXXXXXXXXXXXXXXXXXXXXXXXXXXXXXXXXXXXXXXXXXXXXXXXXXXXXXXXXX
XXXXXXXXXXXXXXXXXXXXXXXXXXXXXXXXXXXXXXXXXXXXXXXXXXXXXXXXXXXXXXXXXXXXXX
XXXXXXXXXXXXXXXXXXXXXXXXXXXXXXXXXXXXXXXXXXXXXXXXXXXXXXXXXXXXXXXXXXXXXX
XXXXXXXXXXXXXXXXXXXXXXXXXXXXXXXXXXXXXXXXXXXXXXXXXXXXXXXXXXXXXXXXXXXXXX
XXXXXXXXXXXXXXXXXXXXXXXXXXXXXXXXXXXXXXXXXXXXXXXXXXXXXXXXXXXXXXXXXXXXXX
XXXXXXXXXXXXXXXXXXXXXXXXXXXXXXXXXXXXXXXXXXXXXXXXXXXXXXXXXXXXXXXXXXXXXX
XXXXXXXXXXXXXXXXXXXXXXXXXXXXXXXXXXXXXXXXXXXXXXXXXXXXXXXXXXXXXXXXXXXXXX
XXXXXXXXXXXXXXXXXXXXXXXXXXXXXXXXXXXXXXXXXXXXXXXXXCTCTTATACCTTTTGTTGGGA
GATAACTACAAAATAGATATACATACATACATAACTCTCTCAAGGAGGAGAGTGAACTAGTATAAATGAA
GTATTTTCCATAAATTGTGTGGAAGCCTCGCTCTTTGAATCGCAACATATTGCCATTCTAGCATTCACAA
TCGAGTTAAATTGAGAGATGCATTGGGAGATGTAGAAGTGAATTAGATAAATTGAATTTTATCCGTTTCG
CTCTGTAGCCTAAACTAAATAATTGCCTTTAATTTGACGATGAGACGATGTGAATAATAATCGCAGTGGA
TGCACTCACAGCACGATTATATTCAACACACAAATGTGAATGACATGCCGCTGCTAGCAAGCAAAATGCA
TAATATAGGAAGTTTTCTTCAATGCAGAAATTGTGAACCCCACTCATAGCCCTCCAATTCTCAAACACAT
CACGCTCTTTTCTGCATAGATTGTCCAATTAAGCGGAGGCATATTGTATGTATCTGGTCGTTTTTTTTTC
ATCCTATACAACATCTCGTATTGTTCATGCCATTGCATCACATTAATTGGACCATAAATGTTCATTAAAA
TGAAAGCGATTCAGATCACAAAATGCTACGATATGAATGAAGTAACACATAAATTGCTTGTATACGGCTC
TGTCCCTCTAATCTCAACACTATTTGTCCCATGACAACGCATTCCGCAGCTTGATGAATACTTTCAGCAT
TTAGCATTCATAAAATTTCGTATACAACAAGCACAGTTCAATGGAATTGATGTATAAAATGATTTTGAAG
TGGAGGAAGAGATATGCAAAAACCATGATTATATTGTGACTGAAATTGAGGCTTTTGTGTATTTTGGATT
CATTAAAAGCATCCTGTGAATGATGTTGAACAGTGGTGTATTCAGGGGAGGGGGATTTGTGTGTCCAAAC
ACCAAATAACGTTAGATTGGTTTTTAGAGAGATCAAAAGAATTATTTTTAGGCATAAAATTATACGGAAA
ATTTTAAAAATTGGATTTACTAATGTTAATAGAATTTTTTTGACATTAGAAAAAAATTATTTCAAAGGTT
TTAAAAAAAGTTAAATGTTGGAAGAAACTTCAAACGTTGAAAGAAACGTCAAACGTTGAAAGAAACGTAA
AAAGATGAAAGAAACGTCAAAAGTTAGAAGAAGCATCAAACCTCGGAAGAAACGTCAAGCGTCGGAAGAA
ACGTCAAACGTCGGAAGAAACGTCAAGCGTCGGAAGAAACGTCAAGCGTCGGAAGAAACGTCACACGTCG
GAACAAATGTCAAATGTTGGAAGAAACGTCAAAATTTGAACGAAACGTCAAACATCAATCAAAAATTAAA
AGAGAAATGTTAACCATTAAAAAAAAAAACTTTTAAAGAAATGTTAATTGTTTGAAAGCAAATTCGAAAT
TTAAAAAAAAATGTCAACTTAAAAAACAGATTTGTTTAACATTACAAAAGGAACTTTAAACGTTTGAAGT
AACTCCAGTCAATAGAAAATAAACGTAAAACATCACAGAAACGTTAGAAAATAAATTCTAAACATGAGAA
ATAATGTCAAACAGTTGTAGGAAAACGTCTAACGTTTTAAGAGATGTCAAACATTAAAAAAAAAGAATGT
CATGAGTTTAAAAATAAACATCAATCGTTATAAAAATAAAATACTTGGTATGCCACGATTGTGGTATAAC
AATTAATTAGCTTTAAAGAAAAACGTCAAACTTTAAAAATGCAAAAAGGCTCCATAATTTGTTAATTGTA
ATACTAACAGTCGTTGTATGATTTAATTAATTAAATTTATTAGGTTGTGCCATAAATTTCCAGATTTCTT
ACCATAATGTACATTTTTTCTTAAAGGTTTTTTTTCTTAAGGAAATATTTTTAATATCTTTTATATTTAA
AAATTTCTTTCTATGATATCATCCTTTTGAATAAATAAATAAATTGAAAAAAAAATTTTGCGAATTTATG
GAAGAGATGGAAAAGCTTTCAATAGCTGCTCTTGTCACTAATTTTATATGCTAATAAAGTTTATTCTAGA
AATTCCATTCTAGCAGAGAATAAATCATTAATTCATTCAAAATATAAATTAATATTTATTTTGGGAATCT
TTAGATGAAAATTGACTCATTTATACTAAAAATCTTTGTTCTAGAAGACTTTCACAAGGTCAAATACATA

AAGAACCTTCTAGAACTGCCCCAGAATCCTCATATTTAGAAACATTCATGCCAAATTCTTATGAATGAAT
AATTCCTCACACACGTAAAAAAAAACTCTTCGCAGCATTTCTAGAAATATTATTTTGATTTCCAACACAA
AACTCCACGGAAAGAACCAAAAGAACCCACCTCAATCCACACATTCTTTTCCCATTCCCTCTCAGCACAC
AGTAGGTTGTTTTGTAATTAAAAGCGAAATGTATGGGTTAGAAGGTAAAATGAGTTTTCTTTTCCTTCAA
CCAATTTTATATGGGAGTGTGAGAGGAGTGCCTAAAAAATACAGAAAGCTCCTCTGTCTATTTATAAAAA
CAATTTAAGCCCAAACACGAAATTTCTGAGTTTGCGGAGAATGTTGAATTGTATAAATATGATTTCTCAC
AG

Neutral alpha-glucosidase
>LLOTMP003489/LlNAglu1
>LLOTMP003489-E1 exon:PUTATIVE_protein_coding
TGGCACTGCTGGTTGTATGGATGACGTTGTGGGAAAAAAAACCATAAATTGATGAAAAATTCAACAAGAT
AAAAAATTCATTGGAAAATTACGTGGAAAATAGTGAAAATACTCGACCGGAAAAGTTAAAAAGTGCTCAT
AGATAAGTCGGAGTAGCTGATAAG
>LLOTMP003489-E2 exon:PUTATIVE_protein_coding
ATGAGGAAACACAATGAGGTTTCTTCTTTTGGGGCTCCTGGGACTCCTAATTACCCTCACGGGGAGTGTT
GATAAGAATAACTTCAAGACATGCGACCAGAGTAGCTTCTGCAG
>LLOTMP003489-E3 exon:PUTATIVE_protein_coding
GCGCTGCCGGAAAGTTGATCCGGGAATCCCTTCTCCGTACGAAGTTCAATTGGGTACACTTAAGACATTC
CCAGATCACATTACGGTGGATGTGCTGAACAAAAATAACGAGCAGGTGTTTAATTTGAAAGTTGTTGGGC
TGAAGGGGAATAAATTCCACGTGGAAGTTGATGAGAAATCCCCGCTGAAGCCACGATATCGCGTTGTGGA
TGCCCTCATAGCACCCCCAGTTACTGAGAATGTCTCCGTGACGAAGGAAGAAGAAGGATCGGTTGTTGTG
AAATGCGGTGAGAATAGAGCTGTCGTCGTGGCATCACCATTCCGCATTGATTTCTACCACGGGGAAGTTC
TTGTGGTGTCAGCAAATGCCAAGGGGCTAATGAAATTTGAGCATTTGCGCAAGAAATCCGTACCTGTTCC
CGCCGAGGCTGCTGAAGAGGGTGGAGAAGGTGCCGCTGCGGCAGAGAATGAGGTGGAGAATCCCCAAACA
AATGAGGATGATGACCCCGGAGCATGGGAGGAGAATTTCAAGTCGCATCACGACAGTAAACCAAATGGCC
CAGAAGCTGTGGCTCTGGACTTTACATTCCCACAAGCTGAGGTACTTTTCGGGATTCCCGAGCATGCAGA
TTCATTCGCACTTAAACCCACTCTTGGCTCTGAACCTTACCGGCTGTACAATTTGGATGTGTTTGAGTAT
GAATTGGATAGCCCAATGGCCCTGTATGGATCCGTTCCTGTCATCTATGGGCATGGAGAGGGCAATACAG
CTGGAGTTTACTGGCAGAATGCCGCCGAGACGTGGGTGGATGTCTACAATTCCAATGCCAAGAAGAACGT
TATGTCGTCTATTGTGAATTTCGTATCGAGATCTCGTCAAGCTGATCCTCCGGCTGCTCATTTTATGTCT
GAAAGTGGAATTATGGACTACTACGTCCTTCTGGGGCCAACTCCCATGGAAACCTTCATGCAGTACGCAG
ATCTTACGGGTCCTGCACCACTTCCGCAAATGTTTGCCCTTGGCTACCATCAGTCACGTTGGAATTACAA
TGATGAGGCTGACGTAGCGGGGGTACATGATAAATTTGACGAGCATGACATCCCCATGGATACGATTTGG
TTGGATATTGAGTACACTGATGCGAAGAAATACTTCACGTGGGACGGACACAAGTTCCCCCATCCCCTGG
AGATGATCAGGAATCTCACAGAGCGCGGGAGGCATCTCACAATCATCATTGATCCCCATATTAAGCGAGA
CGGTGGGTACTTCTTCCACAATGACTGCACAGATCGTGGGTACTATGTGAAGAATAAAGATGGGCGAGAT
TATGAGGGATGGTGCTGGCCCGGAGCTGCGAGTTATGCGGACTTTTTCAGCCCCGACGTGAGGCAATACT
ACGCAGATCAGTACCAGCTTGATAAGTTTGACACAACAACGGCTGATGTGATGCTGTGGAATGACATGAA
TGAGCCATCTGTGTTTAATGGACCCGAAGTGACAATGCTCAAGGATAATATTCACTTTGGTGGCTGGGAG
CATCGTGATGTGCACAACCTGTACGGCCATATGCATGTTCTGGGAACATTTGAGGGTCTTGTGCGGCGCA
GTGGAGGTGTTCAGAGACCCTTCATCCTCACTCGTGGCCACTTTGCGGGCACACAGCGCTACGCAGCCAT
CTGGACGGGTGACAATACAGCCGAATGGGGACATCTCCAGGCATCCATTAAGATGTGCCTGTCTGAGTCA
GTTGCTGGCTTCTCATTCTGTGGCGCTGACGTTGGTGGCTTCTTTGGGAATCCCGATGCGGAGCTCTTTG
AGCGATGGTATCAAGCAGCTGCCTTCCAACCCTTCTTCCGGGCTCACTCCCACATTGACACGCGTCGTCG
TGAACCATGGCTTTGGCCTGAAGCAACCATGCTGGTTGTCCGTGATGCCCTTCGTCGTCGCTACAGCTAT
CTCCCCTTCTGGTACACCCTGTTCTATGAGCACGAACGCACAGGGCGCCCTGTGATGCGTCCCCTTCTCA
CACATTACCCCCTCGACAAGGAAACCTTCACCATTGACTACGAATACCTCCTCGGGGATATCCTCCTTGT
ACGCCCAGTCCTCCAGCAGGGTGTCTCCAAGGTGGATGTTTACTTCCCGGCCGTTGATGGGAAGAAGGAA
GGTGATATCTGGTACGATGTCGATGATCACCGCAAGATTACCGCAGCTGGTTTCGAATCCGTTCCAGTGG
ATAACTACAAGATCCCCGTCTACCAACGTGGAGGAACAATTCTTACGAAGAAGGAACGCATTCGACGTGC
AGCCACGCTCATGGCAAACGATCCAGTCACCTTTGTTGTGGCACTGGATAAGGAACATTCCGCCCGTGGG
ACGCTGTATGTGGATGATGAGAAAACATTCGAGTACCGCAAAGGGAAATACATCTACCTCAATTTGGAAT
TCAAAGACAATGTTATCAGTTGCAG
>LLOTMP003489-E4 exon:PUTATIVE_protein_coding
GAAAAATGATGAGAAGGCCAATTATGATACGAAATCATGGGTGGAACGCATTGTGATTGTCGGTCTGGAG
CACGTACCGAAATCTGCAACATTGAATGTGAGTGGAGAGCCATCGGTGACACTTGAGGTCTACAAACACG
GCGAGAGTGTCGTTGTACGGAAGCCGAAGGTTGTTTTGTCACGGAAGTGGTCAATTCAGCTCAACTATTA
G
>LLOTMP003489-Intron 1:PUTATIVE_protein_coding
GTAAGAATTTGTTGATTTAAAAGGAGTGCCAAAGAGAAAATAACAAAATCATTATTTTTGCAG

>LLOTMP003489-Intron 2:PUTATIVE_protein_coding
GTGAGAATAACTTTATTAACTTTAATTTTTCTCTTGAATTTGACGGGAAAATAATCTCTTTCTAAG
>LLOTMP003489-Intron 3:PUTATIVE_protein_coding
GTGAGTTTTTATTTATTTTTATTTATTGGGAGACATTGAAAAAAGAAGAACTTTCCACGTTTTTTATCTT
ATAATTCATCAGTTTTAAGATTTTTGGTACCTATTTTAGAAAATATTTCAAGAATTGGACAGTTTGTTTC
CTATTTTAATCGTGTTTTTAAAGGTAAAGATTTTTGAAAAAAAATTAATAAAATTATTTTTTGGAGTTCC
CAGTAAACAAAATTTGCTCAATATTGAGTCAATGTATATAGGTCTACATAGATGGTCAATTATTGATCAA
TTTAATTGATAGCTAATAATTGTTCAAATATTGATCGATTACTATCAAATCAGTGCTGTGCTGCGCATGG
AAACTGATTGAGAAGCAATTGATTAAAAAAAAATGGAGCAATTTATAAAAGAACGAGGTGAATATTTGTA
TAGAAAAGAAGGAACGACGATCACCAACAGACCTTTCTTTATTCATTATATAGATAAATCATAAATCATC
TCTTCTATATAATAAAGAAAGGTCTGTTTGTTGGTAATCGTCGTTCCTTCTTTTCTATACAAATCCACAC
CGTTTGACCGATCGCGATGAAATTTGGTACTCCCCGCGAAGCTTTATGATTTCGTAAATCATAAAACATA
AATCATAAATCATAAATCATAAAACATAAATCATAAATTATTAAACATAAATCATAAAACATAAATCATA
AATTATAAAACATAATTCATAAAACATAAAACATAAATCATAAAACATAAATCATAAAACATAACTCATA
AAACATAAATCATAAAACATAAATCATAAAACATTAATCATAAAACATAAATCATAAATCATAAATTATT
AAACATAAATCATAAAACATTAATCATAAAACATTAATCATAAAACATTAATCATAAATCATAAATCATA
AATCATAAATTATTAAACATAAATCATAAAACATAAAACATAAATCATAAATCATAAATTATAAAACATA
AAACATAAATCATAAATCATAAATTATAAAACATAAAACATAAAACATAAATCATAAAACATAACTCATA
AAACATAACTCATAAAACATAAATCATAAAACATAAAACATAAATCATAAAACATAAATCATAAAACATA
AAACTTCGCTCCTTCGCGGGTACTTGTTGCGCTTCGCGCTTTTAGAGAAAAAGTTAATTTGTGATGGAGG
TAGATTAGGGCCACTTATCAATGCCGGAAAAAAATTTTGCAAACAGAAGAAATAATTTTCATTGAACATT
GCCCTTCCTGATCCTATTAGGGATTGTAAAACATAGTCTTGTATTGTGGACTCAATATTTAAAATATAAC
TACCTATACAAAAAAAAAAACATTTTCAAATTCGAGTCTTTGGGTCAGCGTATGACTCAATGAACCTTAA
AGGGGACCTTCATTTAAATATGTTTCATTTTATGCAGAAAACTATTATTACAATACATTACAGCATACTT
TATTGTTTTTACCGACGTGATTATTCAAATTCAGTTTTCCGGGTTCTTTGTCGGATCGGTATGGATGGAG
ATTAAAACACCAGAGTXXXXXXXXXXXXXXXXXXXXXXXXXXXXXXXXXXXXXXXXXXXXXXXXXXXXXTAAA
ACATAAGCATAAAACATAAATCATAAAACATAAAACATAAATCATAAAACATAAATCATAAAATCAAAAA
TACATAAAACATAAATCAAAAATCAAAAATCATAAATCATAAAACATAAATCATAAAACATAAATCATAA
ATCATAAATCATAAAACATAAATCATAAATCATAAATCATAAATCATAAAACATAAATCATAAAACATAA
ATCATAAAACATAAATCATAAATCAAAAATCATAAATCATAAAACATAAATCAAAAATCAAAAATCAAAA
ATCAAAAATCATAAAACATAAAACATAAATCATAAAACATAAATCATAAAACAAAACATAAATCATAAAA
CATAAAACATAAAACATAATACATAAAACATAATACATAAAACATAATACATAAATCATAAATCATAAAA
CATAAATCATAAAACATAAATCATAAAACATAAATAATAAAACATAAATAATAAAACATAAATCATAAAA
CATAAATCATAAAACATAAAACTTCGCTCCTTCGCGGGTACCTGTTGCGCTTCGCGCTTTTAGAGAAAAA
ATTAATTTGTGATGGAGGTAGATTTGGGCCACTTATCAATCCCGGAAAAAAATTTTGCAAACAGAAGAAA
TAATTTTCATTGAACATTGCCCTTCCTGATTCTATGAGTCCTTGGGGATATCCTTTTGTGATCATAAAAC
AATTAGGGATTGTAAAACATAGTCTTGTGGGGGTACCCCCGCTGGCCCCAGGGGTGGGTTTAGCTAATTA
AAACACTTATTTATATAGATAATTCACTCTGCCTCTTTATTGTGCACTCTACACATAACCCTTCACCTGA
CACATCTATCTATCTCCCTCACTCCCAACACCCAGATCACCACATCTTGTATTGTGGACTCAATATTTAA
AATATAACTACCTATACAAAAAAAAAAACATTTTCAAATTCGAGTCTTTGGGTCAGCGTATGACTCAATGA
ACCTTAAAGGGGACCTTCATTTAAATATGTTTCATTTTATGCAGAAAACTATTATTACAATACATTACAG
CATACATGTTAATGAGACATACATAAAAAATAAGAGAGATTTGATGATCTCCCCCTCTTATTTTTTTTCC
TTGTTCTTTCACAG
