# Supplementary material for: Characterization of Glycoside Hydrolase Families 13 and 31 Reveals Expansion and Diversification of α-Amylase Genes in the Phlebotomine Lutzomyia longipalpis and Modulation of Sandfly Glycosidase Activities by Leishmania Infection
Source: Front Physiol. 2021 Apr 9;12:635633. doi: 10.3389/fphys.2021.635633 (PMC8063059; doi:10.3389/fphys.2021.635633)
Supplement: Supplementary File 2 — Protein sequences of L. longipalpis and P. papatasi belonging to GH13 and GH31 after sequence curation. [file Data_Sheet_2.pdf]

File 2. Protein sequences of *L. longipalpis* and *P. papatasi* belonging to GH13 and GH31 after sequence curation. The Xs represent missing amino acid in incomplete sequences.

L. longipalpis Glycoside Hydrolases from family 13

Alpha-Amylase
> LLOJ004838_1/ LlAamyA1
MKFCVFLLLTFVALARGQFDPHFVSGRSGIVHLFEWKWEDIAAECERFLGPNGFAGVQVSPPNENVIVTN
RPWWERYQPISYRIVTRSGNEQQFANMVYRCNNVGVRIYPDIVINHMAASHPVMMGTGGSTANVGNRDYP
AVPYSIHDFNPSCPITNYNDRYQVRNCELVGLPDLNQGVAWVRDRIVEYLDNLVALGVAGFRVDAAKHMW
PGDLEIIYGRVRELNVVHGFPVHARPFFFQEVIDLGGEAISKNEYTGFGVVTEFKFSAEIGRVFRGNDLL
HHLSNWGEAWGFLPSNRALVFVENHDNERGHGAGGDQILTYKNAKQYKMAVAFTLAHPFGIPRIMSSFAF
TNTEIGPPMDGNQNIISPSINADGTCGNGWVCQHRWRQIFNMIRFRNEAGTAGLTNWWSNGSQQIAFARA
GRAFVAFNNQGSNMNVNLQTSLPGGTYCDVISGNVSGGSCSGKTVTVNGDGTANIVIGAAEEDGVLAIHV
GARL#

> LLOJ004838_2/ LlAamyA2
MKILWIFFLAIVATVRGQFDPHYVAGRSVMVFLMDWKYEDVALECERFLGPMGFAGVQVSAASENDIVTS
PLRPWWERYQVVSYKIGSRSGDADSFADMCTRCNNVGVRVYVDAIFNNMGGMGIMKGTAGSLGDSITRTF
PAVPYTILNFHNPPCSITDYNNTQQVRNCDLVGAPDLDQSQPYVRDRIVGYLNHLTSLGCAGFRIDSAKH
MWPTDLQAIYSSVIDLNTAQGFPAGSRSFIFQEVIDSGHEAIKKYTLKKIVYIXXXXXXXXXXXXXXXXX
XXXXXXXXXXXXXXXXXXXXXXXXXXXXXXXXXXXXXXXXXXXXXXXXXXXXXXXXXXXXXXXXXXXXXX
XXXXXXXXXXXXXXXXXXXXXXXXXXXXXXXXXXXXXXXXXXXXXXXXXXXXXXXXXXXXXXXXXXXXXX
XXXXXXXXXXXXXXXXXXXXXXXXXXXXXXXXXXXXXXXXXXXXXXXXXXXXXXXXXXXXXXXXXXXXXX
XXXXXX

>LLOTMP004839/ LlAamyA3
MFWNLFLTTLAIARGQFNPNYVPGRTVMVFLMDWKYQDVALECERFLGPKGFGGVQVSSPAENDIVTDPL
RPWWERYQIISYIIGSRSGNEDDFADMCYRCNKVGVRIYVDAIFNHMGSLPNMVGTAGSTADGPTKSYPA
VPYDASNFHPSCPLTTFQVTFIVRNCELFGAPDLDQAQPYVREKIKGYLDHLITLGIAGFRVDSAKHMWP
KDLKAIYSSLRDLNTDFGFPPKSRPFVFQEVIDAGGGSEPINKYEYSPIGVVTEFLYSYILTDFFRGNRN
LSDLATWGQSEWKFLPSKAALVFVENHDNERATPPVLNYKDGKPYWMAVAFELAHPYGYPYLMSDFNWTT
YDQGPPMDEDQNIISPTINKDGSCGSGWVCQHRWRQIYSMVGFRNQVGYARFTNFWSNGKNQIAFARQGC
GFVAFNNDDVDLNEVLQTTLPAGTYCDVSTGYAKAGECTGRTVVVGDDGTATIEISSSDDIGFIAIHIGQ
ML#

>LLOTMP004841/ LlAamyA4
MRIVVLILCLVALGSGQHWPNFWDGRTGIVHLFEWKFRDVADECERFLAPKGYAGVQLSPVNENVIIPNR
PWWERYQPISYVLTTRSGNEQDFLDMSRRCNAVGIRIYVDVLLNHMSADNYPSDGTAGNWADTSQRWWPA
VPYGPNDFNPRCEIHNWDDPWEIRQCELVGLHDLNQGTEHVRSMLVNFLNHLVELGVAGFRVDAAKHVWP
HDLEVIYNRVNNLNTNFGFAPGSRPYIYQEVIDLGGGGIRYEYTPLGAITEFKFSAEIGGAFRRYNDLRW
LRNFGPEWGFVPSDVALVFVDNHDNQRGHGAGGEAVLTYKQPRQYKMATAFTMAWNYGTIRLMSSFAFDH
GDQGPPQDANENIISPSINPDNTCGNGWVCEHRWRQIYNMIGFANQVKGTSVNDWWDNGHNQIAFCRGDQ
GFIAFNNEDHNFSHTLQTCLPAGTYCDVISGENTGNGCSGVTITVNWDQTAHIVIPVDHYDGVIAIHRGP
TSRIG#

>LLOTMP004880_1/LlAamyB1
MKVLVIFLALVALAKGQFNPYYEDGRTVMAFLMDWKYSDIALECERFLGPKGYAGVQVSSPSENAIVTDP
LRPWWERYHVVSYIIGSRSGTQDDFADMSARCNKCGVRVYVFTIFNNMGAFPDMVGTAGSTADGPSKSYP
AVPYDSTNFHPSCPIQSYQHAFIVRNCENEGTPDLDQSQDYVREQIVAYLNNLISLGAAGFLIDSAKNMW
PTDLKAIYSSLSNLNEDYGFSPNCRPFIYQDVIELGKEAVNKYEYLPLGDVTDYKVSYSLNSIFNGNKSL
SVLATWGESLWNFIPSKSAVVFVENEQNEREGFYFGQVLNYKDGKPYIMAIAFLLAHPYGNPRIMSDFDW
SDSDQGPPMDDNQNIISPTINKDGSCGDGWVCQHRWPQVCSMVTFRNQVGNSNFCNFWSNGNNQIAFARE
GHGFIVFNNNDGKLWQTLPTTLPAGTYCDVITGYVKNNECTGNSIEVADDGTAEFIIYSDDPNGVIAIHI
GQMLE#

>LLOTMP004880_2/LlAamyB2
MRSALIFLAFVVLARGQHNPNFVPGHTGIVHLFEWKWVDVAAECERFLGPMGFGGVQVSSPAENAIVRDP
LRPWWERYHIISYKLVTRSGNEEEFADMCTRCNKVGVRIYVDTVINHMGSLPNMIGTGGSTADGPSKYYP
AVPYNVDHFHTSCPIENWQDEYEIRYCELFGAPDLNQGMGYVRTTIAEYMNHLIRLGVAGFRIDAAKHIS
SSHLYFTTIDLTYLNDLNPDHGFPARTKPFIYQEVIDFGWDAVSKYDYTYIGTVTEFMYSYYISRAFRGN

DLLRNLATWGPTWGFLNSSYALVFVENHDNERGDGAGGQEILSYKDGKQYRMAVAFALAHTYGIPRIMSS
FAFTERDIGPPMDSNENIISPSIYPDGSCGNGWVCQHRWRQVVNMIAFRNISGNENITNWWTNDKNQIAF
ARHQRGFIAFNNDAMNMNVSLQTSLPAGIYCDVISGNLVDKLCTGKNITVRGDGVARILIMKDEEDQVVA
FHVVKQG#

>LLOTMP004880_3/ LlAamyB3
MHFISLGGGILLVLVLHVADGQFDPHFLPGRSVIVHLFEWKFSDIAAECENYLGPNGFGGVQVSPINECL
VSPERAWWERYQPVSYAIVSRSGDEKEFAEMVKRCYEAGVRVYYVDVIFNHMASGEGEVVGTGGSLVYPEE
RLYPHVPYGPEDFNPHCVIEDYQDVDQVRNCALVSLPDLNQKSDNVKRSVIEFLDRLIDHGVAGFRADAC
KHMWPEDIKFLFGSTKNLSPEFGFPDKARPFLYQEVIDLGQEPISKNEYTSIGVVTEFLFSAEIGNIFRY
KKLKEFMKWGTDERFLRSDRALVFVENHDNERGHGAGGKDILTYKDGKRYLLAVLFTIAHPYGIPRIMSS
YDFSNSDEGPPANAKGEIISPVFNDRGLCTNGWICQHRWLGVASMVQFRNAVAGSGIVNWMDNGEQQFAF
CRGDLGFVAFNGYTMSHLNQQLRLTYHLASTDDVISGEVTLDGCTGLEVVVKNDGYANIFIPGDSPTGVL
AVHLGSAYIID#

>LLOTMP004881_1/ LlAamyB4
MKATIFALLSLLAQTKGYFNPNFVPGRSGIVWLFEWKFADIALECERFLGPNGFAGVQISSPAENAIVTN
PLRPWWERYNPVSYIIASRSGNEQDFSDMVTRCNNVGVRTYVEIIMNNMGGYPNMVGTGGSTADGPSRSF
PAVPYTSADFHATCSITTMLLPFLIRNCAVEGLPDLNQSGAHTRDMIVGYLNHLISLGVAGFRINSAKFV
WPSDLKVIYDRTNNLNTNYGFAPNSRPFIFQDVADYGLDAISKYEYSSMAVVSEYKFLLNLGRIFRHLDV
LSVLTNWVQTFGLLPSDAALVFVDSPDSERGSQGSEILTYKDGKAYILALIYMLAVPYGHPIIMSSYEFK
DVNSGPPMDANQNIVSPTINPDGSCGSGYVCQHRWTPVSSMVAFSNNVGDAPVTNVWTNGQNQLAFARDG
VGFVAINSNAETLDVSVQTGLVGGVYCNVLGGPIVDGTCAGQTVVVGDDGIAAITITTQDNLGAIVLDVN
QQLLNGEAKKFKQEHPSLY#

>LLOTMP004881_2/ LlAamyB5
MKFFMILLALVASTRGQFNPYFWPNHNTIVYLLDWKYSDVALECENFLGPKGFGGVQVSSPSENAIITDP
YNRPWWERFHVLSYIIGSRSGNQDDFADMTARCNKCGVRVFVDAIFNEMGGLPDMVGTAGSTGDGPSRTF
PAVPYDSSNFHPPCPIQSWLHAFIVRNXXXXXXXXXXXXXXXXXXXXXXXXXXXXXXXXXXXXXXXXXXX
XXXXXXXXXXXXXXXXXXXXXXXXXXXXXXXXXXXXXXXXXXXXXXXXXXXXXXXXXXXXXXXXXXXXXX
XXXXXXXXXXXXXXXXXXXXXXXXXXXXXXXXXXXXXXXXXXXXXXXXXXXXXXXXXXXXXXXXXXXXXX
XXXXXXXXXXXXXXXXXXXXXXXXKKGTVCDSPYVCQHRWPQVCSMVTFRNQVGDANFCNFWSNGNNQI
AFAREGQGFIVFNNNDKKLWQTLQTTLPEGTYCDVITGYVKNDECTGRSIEVADDGTAEFIIYTDDPNGV
IAIHTGQML#

>LLOTMP004881_3/ LlAamyB6
MLKFVVFLALVAFTRGQFNPYFWPGHNTIVYLLDWKYSDVALECENFLGPKGFGGVQVSSPSENAIITDP
YNRPWWERFHVLSYIIGSRSGNQDDFADMTARCNKCGVRVFVDAVFNNMGGLPDMVGTAGSTGDGPSMTF
PAVPYDSSNFHPPCPIQSWLHGFIIRNCEDPDFLPVPDLDQSQDYVREQIVTYLNNLISLGAAGFRIFAA
KHMWPTDLEAIYSSLSNLNEDYGFSPNCRPYIYQDVIDLGGEAVNKYEYIPLGLVSEYNASQSLADIFRG
NRPLSVLATWGEAEWNFVPSANAIVFVENEQNELDTWYFGRVLNYKDGKPYIMAIAFLLAHPFGNPLLNS
DYEWTDPNEGPPMDDDQNILSPVINKKGTCDSPYVCQHRWPQVCSMVTFRNQVGDANFCNFWSNGNNQIA
FAREGQGFIVFNNNDKKLWQTLQTTLPEGTYCDVITGYVKNDECTGRSIEVADDGTAEFIIYTDDPNGVI
AIHTGQML#

>LLOTMP004882/ LlAamyB7
MKIFWIFFLATVATVRGQFDPHYVSGRSVMVFLMDWKYEDVALECERFLGPMGFAGVQVSAASENDIVTS
PLRPWWERYQVVSYKIGSRSGDADSFADMCTRCNNVGVRVYVDAIFNNMGGMGIMHGTAGSLGDSITRTF
PAVPYTILNFHNPPCSITDYNNTQQVRNCDLVGAPDLDQSQPYVRDKIVGYLNHLTSLGCAGFRIDSAKH
MWPTDLQAIYSSVIDLNTAQGFPAGSRSFIYQEVIDSGHEAIKKYTXXXXXXXXXXXXXXXXXXXXXXXX
XXXXXXXXXXXXXXXXXXXXXXXXXCNEPLSTLSTWGQSNWGFLPSDRALVFVENHDNERGQYESAALN
YKDGKPYKMAVAFALAHPFGNLRIMSDFDWTTYDQGPPMDANQDIISPTINADGSCGSGWVCQHRWRQIY
GMVGFRNTAGTTEVTNFWSNGNNQIGFARQGRAFVAFNNNDADMNQTLQTTLPAGTYCDVITGVAENGAC
TGRTIVVANDGTANFVILTSDADGVIAIHVDQKL#

>LLOJ004885/ LlAamyB8
MKLFVVFLALVAFTRGQFNPYFWPNHNTIVYLLDWKYSDVALECENFLGPKGFGGVQVSSPSENAIITDP
YNRPWWERFHVLSYIIGSRSGNQDDFADMTARCNKCGVRVFVDAIFNEMGGLPDMVGTAGSTGDGPSRTF
PAVPYDSSNFHPPCPIQSWLHAFIVRNCENADYLPVPDLDQSQDYVREQIVTYLNNLISLGAAGFRILHX
XXXXXXXXXXXXXXXXXXXXXXXXXXXXXXXXXXXXXXXXXXXXXXXXXXXXXXXXXXXXXXXXXXXXXX
XXXXXXXXXXXXXXXXXXXXXXXXXXXXXXXXXXXXXXXXXXXXXXXXXXXXXXXXXXXXXXXXXXXXXX

XXXXXXXXXXXXXXXXXXXXXXXXXXXXXXXXXXXXXXXXXXXXXXXXXXXXXXXXXXXXXXXXXXXXXX
XXXXXXXXXXXXXXXXXXXXXXXXXXXXXXXXXXXXXXXXXXXXXXXXXXXXXXXXXXXXXXXXXXXXXX
XXXXXX

>LLOTMP005909_1/ LlAamyC1
MHFISFGGGILLVLVLHVADGQFDPHFLPGRSVIVHLFEWKFSDIAAECENYLGPNGFGGVQVSPINECL
VSPERAWWERYQPVSYAIVSRSGDEKEFAEMVKRCYEAGVRVYVDVIFNHMASGEGEVVGTGGSLVYPEE
RLYPHVPYGPEDFNPHCVIEDYQDVDQVRNCALVSLPDLNQKSDNVKRSVIEFLDRLIDHGVAGFRADAC
KHMWPEDIXXXXXXXXXXXXXXXXXXXXXXXXXXXXXXXXXXXXXXNEYTSIGVVTEFLFSAEIGNIFRY
KKLKEFMKWGTDERFLRSDRALVFVENHDNERGHGAGGKDILTYKDGKRYLLAVLFTIAHPYGIPRIMSS
YDFSNSDEGPPANAKGEIISPVFNDRGLCTNGWICQHRWLGVASMVQFRNAVAGSGIVNWMDNGEQQFAF
CRGDLGFVAFNGYTMSHLNSTVKVCLPPGIYCDVISGEVTLDGCTGLEVVVKDDGYANIFIPGDSPTGVL
AVHLGSAYIID

>LLOTMP005909_2/ LlAamyC2
MKFLIGFGVVLILVAVAAQYDPHYLDKRTVMVHLFEWKWNDIAKECQDYLGPNGFGGVQVSPVNENWVSG
ERAWFERYQPISYKLTTRSGTEDEFAAMVKTCRQNGVRIFVDIVVNHMASGALEDTIYGTGGSEAHPGPF
DYPAVPYEKNDFHPDCSISDYQNVYQVRNCQLSSLRDLNQTIPYVREKILDFLNHLVDLGVAGFRIDAAK
HMDPKDLRYIYNHIKKLNRTLSFRAGDKAFIFQEVIDLGGEAVSSREYISLGVVTEFKASDDLGKLFRGQ
VALSTLERWGPQYGLLPSNRALAFVENHDNERGHGAGGTNILTYKDGKIYTMAVVFNLAHSYGVPRMMSS
YEFNDPSQGPPHDDNNNILTPEFSADGNSCTNGWVCQHRWRPMRNMVKFRNIVGRKPVRKWYDNGSNQIA
FSRGNQGFVAFNMDIVDFNQQVPTDLPDGVYCDVITGEKNGNECTGKVVIVSKRKAAVILRADDDYGVLA
IHSESKL#

Maltase
>LLOTMP000566/ LlAglu1
XXXXXXXXXXXXXXXXXXXXXXXXXXXXXXXXXXXXXXXXXXXNIGITQRLQYVKDLGMSGTWLSPIFKS
PMADFGYDTADYTAIQPEYGTMEDFEALIKKANEIGIKIILDFVPNHSSDQHEWFKKSVDRVPGYEDFYV
WHPGKIVNGQRQPPNNWVSVFRGSAWTWNEKRQEYYFHAFLKEQPDLNYRNQATVDAMKETLRFWMQKGV
FGFRIDAVPYLFEVPADSNGNIPDEPLTGPSCPDPTHDCYTQHIYTQNLDETFDMVYQWREVVDEFAKQT
DNVPRILMIEAYTPLENMKRLYEDGHGREGAQLPFNFELISKLNGKSTAKDFKDVIDGWISRLPQGKENN
WVLGNHDNKRIASRFGIDRADLINILLQTLPGMAVTYNGEELALKDVYISWEDTIDPAGCNTSPDVYEQY
SRDPVRTPFPWNANKNAGFSNSDKTWLPVSSDYKEVNVEAQEKATRSHLKSFRKLTKMHQHEKAFTQGQL
LMKLVGTDILAYERRVPGTKPEENFVMILNFSNNTHKVNIPQLFPQMSQMYRIEVVSMHSLSHKEGDTID
GKNFEVKPNEAFVLRGGAPRLYSYLILCFTMFSAFFNINLTRA#

>LLOTMP008156/LlAglu2
MRVIIFVFLVISATTEGVWYNNGNFYQIYPRSFMDSNNDGVGDLKGITQRLQYVKDLGMSGTWLSPIFKS
PMADFGYDTADYTAIQPEYGTMEDFEALIKKANEIGIKIILDFVPNHSSDQHEWFKKSVDRVPGYEDFYV
WHPGKIVNGQRQPPNNWVSVFRGSAWTWNEKRQEYYFHAFLKEQPDLNYRNQATVDAMKETLRFWMQKGV
FGFRIDAVPYLFEVPADSNGNIPDEPLTGPSCPDPTHDCYTQHIYTQNLDETFDMVYQWREVVDEFAKQT
DNVPRILMIEAYTPLENMKRLYEDGHGREGAQLPFNFELISKLNGKSTAKDFKDVIDGWISRLPQGKENN
WVLGNHDNKRIASRFGIDRADLINILLQTLPGMAVTYNGEELALKDVYISWEDTIDPAGCNTSPDVYEQY
SRDPVRTPFPWNANKNAGFSNSDKTWLPVSSDYKEVNVEAQEKATRSHLKSFRKLTKMHQHEKAFTQGQL
LMKLVGTDILAYERRVPGTKPEENFVMILNFSNNTHKVNIPQLFPQMSQMYRIEVVSMHSLSHKEGDTID
GKNFEVKPNEAFVLRGGAPRLYSYLILCFTMFSAFFNINLTRA#

>LLOTMP002257/ LlAglu3
MKLLGLLLVTVLTVEGHLLGERQSSEKADLRHDHPELDWYERANLYQIYPRSFMDSDGDGIGDLNGITAK
LEHLQESGIFATWLSPIFSSPMRDFGYDISNFTEINSDYGTMEDFLQLLHEARRLNIRLILDFVPNHTSD
LHEWLVKSEDNDPEYRDYYVWRDARYVGEERHPPNNWLSVFHGPAWTWSEKRQQYYLHQFAKEQPDLNFE
NEKVVEEMTNVLKFWLDIGVDGFRVDAINHLFEDPSFADNPPNREIYDPMDYSHFDTIYSKDLPKSYQQV
YDWRKYLDSYAEEHGTDTRVLLTEAYTNITNTMKWQMSEDGTQKGAHFSFNFNLIMELKTLPDELNAADV
KATIDEWLEHLPDGVPPNWVLGNHDRPRVASRFGVDLVDNMNILVQTLPGVAVTYYGEEIGMEDFRGISW
EDTQDPQACGSNETVYQLYTRDPVRTPMQWDDTQNAGFSTNPSPWLPVHPDYVQNNLAQQKVAETSTYKL
FLDLVKLRIDHALEYGDFKSEALSNGVYAYSRNMEGHESLVVALNFASTEATVDLTPVLLENSHSEGLVE
LATTKSIHKRDDVVNLRTLTLQQYDGVILRVNSSAATIFISSFALLLVVLKTLLY#

Amino acid transport protein
>LLOTMP006803/ LlCD98hc

MTDKTERNSTKLNLEAQRLSASDSPFPKETYKTIPDLDHHQKAEKKLEDGLCDLLSHCVFCMVISDAVKN
SNMVREKVDMSEEGADEKMLGAGEEEQQKLAKKEEVKFISGDQQNGDAKIDIGAIDKTFTGLTKEELMKY
ANDPFWIRLRWFLFICFWGLWAGMLVGAVLIIIYAPKCAAPEPLSWWKQGPLVKIDTPTPDNIQIEGIKG
LGVKGVIYEVPADETYNIGSDPAISDALKKLAASFAAYGINLIVDLTPNFVSRGDPLFQKALNNPDVRSA
FVTREGHQVPNNWLSLVNGSAWTNEGNLIFLQQFGHDRFDLQLNDPVALTKLKGVLKEVVSLGAKGIRLA
NTKHFIVDRELKDNVPSNNPNYDHTQYGFWTHAHTTYQDGLGDLLYDLKAYVHNITNSEGFLAASDDIDR
PEVYMSSRGVQSLDMIQLGRVTNLLANSTSADHIYRELQNVWKSTVETGKHFWLQWNYVKQELLQQITIS
EYNIFLQLIPGVPVFNVDASKAKAEDYSTEAFHKIRTSPSYMHGSFDLYRDVNSTVIGYSRTKSGNPGFF
VVFNPTEQYVNANFSNVVGIAEELTVHTLSTNYNATDVAVKGKVSSSSIPVSRYSAIILTYVPKA#

>LLOTMP008629/ LlNBAT1
MNNLGISTDPALLGVDLPSSLTTSPSVSTFMPEEDASICPLLPTTPSPPPMDFIHPLTPSTGIDEGNNED
LGGEFQGDDAQADSSSSGSSSGIGIQVTANGPGSLFNKHAYQHLGSKNGDITQDNGVTQTGVFSIPMTKD
TPSFVNWNWPLIRKCTFFLFMSGLFAMCAIVVAMMFKLPKSCNPEVPWYKGAVFYEIFPASFQDSNGDGL
GDLKGLASRIDYLESLGVGAVRLNSIFPAKHYPDHFQNVTTLLDIDEILGNPRDLTYLARILQRRNISLI
LDLPIYPLVKHLAVIRENFTEATNSTDTEDEEVFQDSDTSEDPVLTAMRFWLSMGVDGFYVKGLENYAGD
PYLIENLQEWKFALGSDRILMVSKLVFDSVDDDTAEKVRHCVDLVDVFVDVSNGTKVIAEKVQSILNSRF
LAPGFGPWIHWSLGGVTERRLAQGTSSNISLAATLMQLMLPGTPSIFYGDEVALQEVYDPLGEHEESKHL
HHLSTMVWDSEIQFTVKEHLPWLPRGAFVAFHHFEHVAGMIRLRGISPSIYQNAVIKEHQSVLNTAVRYS
KNDILILERWYPRRNTFTSISNFGSKSLVLDLSGMFYAGEIMLGSLKGEKVFFSSIQVKPMETIIVKLDK
#

1,4-alpha-glucan-branching enzyme
>LLOTMP005533/ LlAGB1
MDPMKVEVPEIDKLFAIDGYLRPQERELRRRHGVIKDWLKKIDGNEDGGVDGFSQAYKYYGIHIQPDNAV
IAREWAPGAQQLYLTGDFNDWQWEATPYTKLEFGKWELKIPPREDGSCAIRHLSEIKVIVRTQEGTLVDR
LSPWAKYVLQPPKEANQGTNFKQYLWHPPPHEKYMFRYGRPQKPQSLRIYECHVGIATEELGVGKYRDFG
DKIIPRIVKQGYNAIQVMAIMEHAYYASFGYQVTSFYAASSRYGTPDELKYMIDMAHKAGLYVLLDVVHS
HASKNVADGLNRFDGTNTGFFHDGARGEHPLWDSRLFNYTEYEVLRFLVSNLRWWHDEYNFDGYRFDGVT
SMLYHSRGVGEGFSGDYNEYFGLNVDTDALVYLAIANEILHKLDKDIITIAEDVSGMPTMCRPVSEGGIG
FDYRLGMAIPDKWIEYLKEKRDDDWNMGNIVHTLTNRRWMEKTVAYAESHDQALVGDKTLAFWLMDKEMY
THMSTLSEPPLIIDRGIALHKMIRLITHSLGGEAYLNFMGNEFGHPEWLDFPRVGNNDSYHYARRQWHLV
DDEMLKYKYLNEFDRAMNGLEMKYGWLACDPAYVSCKHEDDKVIVFERAGLLFAFNFHPSKSFTDYRLGV
EVGGLYRIVLSTDDPLFGGMNRVDVKCDHLSTPEGYAGRRNFIQAYLPARTAFVFAKFD#

Glycogen debranching enzyme
>LLOJ008312/ LlGDE1
MGHQDVLSLAISHGEDKEGTLFRLKRGTILHIVPGPNLLGRHIALYCNYPQNGEKGFERTKYSTLIWHTS
SGQKLTNAAQPFVEVNDLDIYCEIVANKSGTFHFYFSFQESPKEREGSLYVQVEPKIKVGPKNAEREIPL
DSIRCQTVMAKCLGHLETWEAKLRVTHESGYNLLHFTPIQKLGNSRSGYSLCDQLSVNPDFGPSANFDKV
AKIVKKCREEWGIASICDIVLNHTANESPWLKEHPETTYSCSTCPHLRPAFLLDSVLAMASSDTGNGLLE
TFGVPVEIDREDHINALRHQLHSNYLPKARIHELYQCNVEEYVKKFSEEMRKRPPPKAPKESQSKETIVL
KQDPAYRRLACTIDFESAFEIFNVFRNDAFDEDTRLRKCAEAFRQHLEELNEVVRCEIAEHLRSAVENCL
SGLRYERIQDDGPRLREISIKHPLFCRYFTDGDVTCKTIEDIEALMYGDSAKYLMAHNGWVMNGDPLKDF
ARPQPTTANVYIRRELIAWGDSVKLKYGDKPEDCPYLWDHMKKYVEITAKIFDGVRLDNCHSTPLHVAEY
LLDAARKINPELYVVAELFTNSDHTDNIFVNRLGITSLIREALSAWDSHEEGRLVYRYGGAPVGAFFSSP
KRLLAPCIAHALFLDLTHDNPSPIQKRSVFDLLPSAGLVSMACCATGSTRGYDELVPHHIHVVDEEREYQ
EWGKAVDAETGIISGKKAINLLHGELAEKGFSEVFVDQMDYNVVAVTRSCPTTRESVILVAHTCFSYPDP
YSGPTNVRSLRFEGHLEEIILEAGVTHKSSKPYDCPYRYEKDEKYINGLTEYQLKIVEHIPLEKSTIFST
EMVKDGNITQLNFKNLTPGSIVAVRVSLHERTRPHFANVQKLVDAFHFQKGQIFSDLQKIVSKLNLIDLN
RALYRCDEEEKDMGMGSGTYDIPGFGRLVYSGTQGFASALSVIGPNNDLGHPFCDNLRRGDWMIDYIRDR
LARWSGTKALSEWWATNTASLKEMPRYLIPSYFDVILTGVNALLLEQSVTLMSDFVRKGSSFVQSLAMGS
VQCVAECPSANLPALSPQTKAPKPPNQCATMSAGLPHFSTGYMRCWGRDTFISLRGLLLLTGRFDEARYM
ILGFGSCLRHGLIPNLLDGGYKARFNCRDAIWWWLYSIKYFVEEAPKGVEILTEKVSRIFPMDDSEARKA
GECDQALCDVIQEAITVHFQGLVYRERNAGPAIDAHMTEKGFNNQIGVHPDTGFVFGGNDANCGTWMDKM
GSSEKARNRGVPSTPRDGSAVELVGLQMATLRFLQKMSTEGKFPHKSVERTSKNGTKTVWTYKEWADRIK
ANFEKNFFVGQGEIPLANKRGIYKDSCGATQTWQDFQLRPNFPIAMVAAPELFDPKHAWEALEQARKYLL
GPLGMKTLDPQDWGYHGDYDNSNDSEDPKVAHGANYHQGPEWVWPIGFYLRARLIFAAQNNRLKDTVAET
WAILTAHLKEVKSSWWRGLPELTNSNGSFCSGSCTTQAWSMATVLEVLYDLERLKCKS#

L. longipalpis Glycoside Hydrolases from family 31

Glycosidase NET37

>LLOJ001847_1/ LlGlyMyo1
MFPSMIKKKKKSKDFKIKAFVACLFLIIVFLVGYAYIMYNQQVLARSYFDRVKLNKGQRVIGIYNEKGVL
MVTGVWVRLYIQRKPTTASRIIRWKMEVCVWNGMARRGCISIFQELSPSVPCYTIRWQSLSPDVLPTDCY
ESFMGQGHWYGGGLTRGGNWPLETESFPFAPFITGDAKRQQWGNVLKRYFISSRGVAIQVDEKSPLYVSM
NDNKSGEMCFRAKHDHFAFVNRLTPLPELKYRICTSDNMRQLHQQMTQQSLWDGLKEHDINVVHSLLEEP
VWQIPSSGGKGITGDSIHNYTERVIALGFLRLGHVLVNEFWQRHIGDFTLETERFPNLEETVTILHRRGF
RIVFSVQPYISTDSDNFAQSVAQKLLVYERQSERSIPALTRYKSVASAGVLDITNNASIPWLIEKLEKIQ
KTYKIDGFYLDFGTSQNMPHYYQCNKTLYNPDQYKTIFTTALEGVISVIGVSSAVTVPRPPAFVSLPPVN
SSWEGLRTVVTSALTYGIIGYPFIMPGPIGGDYLLPPSASNETVSFYFMEEPPLPDQELYLRWMQLATFL
PVIRFTHLPSEYKSELIMEAVKELTLIRQKAVIPLLKKYLSDAMNEGLPLIRPLWMLDAQDTACLYVNDE
FSIGEDLIVAPILEKGQLQREVYLPQGVWKDGIDGSLRKGSRWIHNYRVPEDKVAYFMKMPDNTRF#

>LLOJ001847_2/ LlGlyMyo2
MIKKKKKSKDFKIKAFVACLFLIIVFLVGYAYIMYNQQVLARSYFDRVKLNKGQRVIGIYNEKGVLMVTG
RLGTTIHSEKAYHCLTNNTLEDGSVCLEWDGKARMYLNFQELSPSVPCYTIRWQSLSPDVLPTDCYESFM
GQGHWYGGGLTRGGNWPLETESFPFAPFITGDAKRQQWGNVLKRYFISSRGVAIQVDEKSPLYVSMNDNK
SGEMCFRAKHDHFAFVNRLTPLPELKYRICTSDNMRQLHQQMTQQSLWDGLKEHDINVVHSLLEEPVWQI
PSSGGKGITGDSIHNYTERVIALGFLRLGHVLVNEFWQRHIGDFTLETERFPNLEETVTILHRRGFRIVF
SVQPYISTDSDNFAQSVAQKLLVYERQSERSIPALTRYKSVASAGVLDITNNASIPWLIEKLEKIQKTYK
IDGFYLDFGTSQNMPHYYQCNKTLYNPDQYKTIFTTALEGVISVIGVSSAVTVPRPPAFVSLPPVNSSWE
GLRTVVTSALTYGIIGYPFIMPGPIGGDYLLPPSASNETVSFYFMEEPPLPDQELYLRWMQLATFLPVIR
FTHLPSEYKSELIMEAVKELTLIRQKAVIPLLKKYLSDAMNEGLPLIRPLWMLDAQDTGLLYVNDVIFNL
VEDLIVAPILEKGQLQREVYLPQGVWKDGIDGSLRKGSRWIHNYRVPEDKVAYFMKMPDNTRF#

>LLOTMP001881/ LlGlyMyo3
XXXXXXXXXXXXXXXXXXXXXXXXXXXXXXXXXXXXXXXXXXXXXXXXXXXXXXXXXXXXXXXXXXXXXX
XXXXXXXXXXXXXXXXXXXXXXXXXXXXXXXXXXXXXXXXXXXXXXXXXXXXXXXXXXXXXXGPQQKYQY
WPVEKLVLSNYSYVTKEADSAAIAERYWLNSEGAFIYLSDRAPLFIDQNSMMDNHLCFVASLALPYNPRA
QTYDFEYSIGVASDPRKAHMEAVHRFLGKPSGVPDRLMAARPIWSTWARYKRDVDQYVVMEFAKEIIDNR
FTNSQFELDDDWEICYGALTFSTTKFPNIRKTVQDLKSMGFRVTLWIHPFINKGCNPWYNEALTNRYFVL
DHNGKEDTQWWNSEQGEAAYIDFTNPEAANWYTSRLHKLLEDTGIDSFKFDAGESSWQPDDPVLPSTSLD
QHPLKIQTDYIMHVAQFGPIVEVRSGFRNQFQQIYMRMIDKDSLWSWNNGLPTLITTLLQLNMNGYPLVL
PDMIGGNGYEGAPSREMFIRWLQANVFMPSLQFSYVPWDYSDGDGIKILDLCRHFVKLHEDYTDLIMERF
QKAVDHGEPVNPPIWWVDPKNPVAQSIYDXXXXXXXXXXXXXXXXXXXXXXXXXXXXXXXXXXXXXXXX
XXXXXXXXXXXXXXXXXXXXXX

>LLOTMP000840/ LlGlyMyo4
MEKKRVKGNPKYKSAFKWGTAGIIATVIFVLVEVFIIHWVTPFDNFLRVHFPKAGVHVQFECEHIEDGVA
FSIHKNRTLVQIVRMGDNLGWDLRVEDHGGGKYVLRSREGSVSFNTAVDEDGLAVFHINQTIRTTDFVEH
CFDLVNPDGNNWFAGPHKYYQHWPSQVLQFTDDAYLPKESSHSSISERYWLTSKGMFIYFSDRTPLFLNR
KPNHLCFAAKKQNPYYTYDTVFVFNYTIGIAADARKAHKAAVGRYLRKPLHYPDRRVIQHPKWILRNHNM
SQVQEYIDNLLYYNFSTSSIILDKSWETCTGALEFDPIKFPNVGSMIRYFRRHRTHLTLTVTPYIHEDCN
PYFQDALSRGFLVKTHQNTYLNFDRTATVDFTQVAARRWFLARLKDLQALGVDNFYFEGGEFDSAPNDPN
FEGVSTSLHPIQYTIDSLRALAEFDHNTIVRTGFCTQDLPLLLRLPDMDNRWDIQNGLDSLIPKILQTNL
NGYYFLMAPIGGTTSEGLTKELYIRWMQAVVFLPSMAFSTPPWEFDDETIELAQKFIRLHMRHVSLFVDL
FKLAKSDGDPVNLPIWWLYPQNWRAQETHDQYLLGEDIIVAPVLKPNSLERGIFLPYGIWRDGNDNSSLY
QGPRWLPSYRAPLDVLPYFVRARNKN#

Lysosomal alpha-glucosidase

>LLOTMP006451/LlLysAglu1
XXXXXXXXXXXXXXXXXXXXXXXXXXXXXXXXXXXXXXXXXXXXXXXXXXXXXXXXXXXXXXXXXXXXXX
XXXXXXXXXXXXXXXXXXXXXXXXXXXXXXXXXXXXXXXXXXXXXXXXXXXXXXXXXXXXXXXXXXXXXX
XXXXXXXXXXXXXXXXXXXXXXXXXXXXXXXXXXXXXXXXXXXXXXXXXXXXXXXXXXXXXXXXXXXXXX
XXXXXXXXXXXXXXXXXXXXXXXXXXXXXXXXXXXXXXXXXXXXXXXXXXXXXXXXXXXXNVFKFACFNF
DFRISIILDATTEMFHGIYINAPYPVEVEITESYLVIVRAIFDTSFHIDVLCGPTIGNLMDQLMEQTEQN
LHPLPNWFFGFHICDTNITRNLTDSVIEVLELLSSDLPFDTHCIREQLLWLGDDIKGLPSEVLPVDLKSA
AYHMAKAKGLLMRHPTLEEPYIGRIGKTNVVYVDWVGSNQENLYEWSNQFMPNNLSADGYMVQANWMWDQ
SDGEKIPHSFPYISMDMINASKNIVPWFIRQSGTDGKASIFSHNFAASAQIDVIRKVLGNESFILSESSL
FANVPISRRQVPSTWTELRNLVRRTMGQSISGIYFSSANICGDGETWTEELCIRWYQFASLSPFYRVASA
KAPTSFSKFAQSLLRASTERRYSLYLYIHTVVTERRPLLTPLFYEYPDLIALMDNLTHQALVGPSLLFAP

VLLPGVQHINIFFPEVYYEIGGGQQLIANTWAELPVVETDAPLFIRAGHIVPIQHTKNVRSITVMQMKPI
HLYVALGPLENGISTAIGKVQFDSEYKVTFNAAYTTSSEQTQLKLSSYSKLTCRTTTNLTIESRCRVEEG
FLPAIFEKFQPLSYXXXXXXXXXXXXXXXXXXXXXXXXXXXXXXXX

Neutral alpha-glucosidase
>LLOTMP003489/LlNAglu1
MRFLLLGLLGLLITLTGSVDKNNFKTCDQSSFCRRCRKVDPGIPSPYEVQLGTLKTFPDHITVDVLNKNN
EQVFNLKVVGLKGNKFHVEVDEKSPLKPRYRVVDALIAPPVTENVSVTKEEEGSVVVKCGENRAVVVASP
FRIDFYHGEVLVVSANAKGLMKFEHLRKKSVPVPAEAAEEGGEGAAAAENEVENPQTNEDDDPGAWEENF
KSHHDSKPNGPEAVALDFTFPQAEVLFGIPEHADSFALKPTLGSEPYRLYNLDVFEYELDSPMALYGSVP
VIYGHGEGNTAGVYWQNAAETWVDVYNSNAKKNVMSSIVNFVSRSRQADPPAAHFMSESGIMDYYVLLGP
TPMETFMQYADLTGPAPLPQMFALGYHQSRWNYNDEADVAGVHDKFDEHDIPMDTIWLDIEYTDAKKYFT
WDGHKFPHPLEMIRNLTERGRHLTIIIDPHIKRDGGYFFHNDCTDRGYYVKNKDGRDYEGWCWPGAASYA
DFFSPDVRQYYADQYQLDKFDTTTADVMLWNDMNEPSVFNGPEVTMLKDNIHFGGWEHRDVHNLYGHMHV
LGTFEGLVRRSGGVQRPFILTRGHFAGTQRYAAIWTGDNTAEWGHLQASIKMCLSESVAGFSFCGADVGG
FFGNPDAELFERWYQAAAFQPFFRAHSHIDTRRREPWLWPEATMLVVRDALRRRYSYLPFWYTLFYEHER
TGRPVMRPLLTHYPLDKETFTIDYEYLLGDILLVRPVLQQGVSKVDVYFPAVDGKKEGDIWYDVDDHRKI
TAAGFESVPVDNYKIPVYQRGGTILTKKERIRRAATLMANDPVTFVVALDKEHSARGTLYVDDEKTFEYR
KGKYIYLNLEFKDNVISCRKNDEKANYDTKSWVERIVIVGLEHVPKSATLNVSGEPSVTLEVYKHGESVV
VRKPKVVLSRKWSIQLNY#

P. papatasi Glycoside Hydrolases from family 13
Alpha-Amylase
>PPATMP010150_1/ PpAamy2
MEFCIILFIALVPNVLGQFDPHYAAGHNVIVHLFDWKWVDIAAECERFLGPNGFGGVQVSSPAENAIVKE
PNRPWWERYQIISYILQTRSGSQEEFADMVRRCNNARVRIYVDAIFNHMGGTGADMRGTGGSTANSVIRS
YPAVPYDILDFHVPCPIFDYQNKDNVRNCDLVGAPDLNQALEDVREKIVDYLSRLIALGVAGFRIDSAKH
IWPNDLKVIFGRLPNLMTDHGFPPDAKPFIYQEVIDFGGEAISKYEYIELGAVTEFRYSFNISGVFRGLA
RLDTLQNWGEPWGFLPSDLALVFVENHDNERNHGAGGAQILTYKDGKRYIMAVTFGLAHPYGISRIMSSF
FFTDTEAGPPMDANEAIISPSINPDGSCGNGWVCQHRWRQIYNMVKFKNVAGTAPLLNWWSNGNNQIAFA
RSGKGFVAFNNEGSDMDVILETSLPAGTYCDVISGSVSGTTCSGKTVIVQTDGTANIVIGNAEEDGVLAI
HIEAKL#

>PPATMP010150_2/ PpAamy3
MNLSFFVFLALVAVAHGQFNPNFVSGRNGIVHLFEWKWEDIAAECERFLGPNGFAGVQVSPPNENVIVNN
RPWWERYQPMSYRLVTRSGNEQQFANMVYRCNNVGVRIYPDIVINHMAAGHPNMIGTGGSTANVGNRDFP
GVPYSIHDFNPSCPINNYQDRYQVRNCELVGLPDLNQGVAWVRDRIVEYLDNLVNLGVAGFRIDAAKHMW
PGDLEIIYGRVRNLNTVHGFPANARPFFAQEVIDLGGEAVSKNEYTGFGVVTEFKFSAEIGRVFNGNDRL
THLSNWGEAWGFLPSNRALVFVENHDNERGHGAGGDQILTYKNAKPYKMAVAFTLAHPFGIPRIMSSFAF
TNTDIGPPMDGNQNIISPSINADGTCGNGWVCQHRWRQIFNMIRFRNEAGSAALTNWWSNGNNQIAFARS
GRGFVAFNNEGSNMNVNLQTSLPAGTYCDVISGNVSGGSCTGKTVVVQGNGNANIVIGAAEYDGVLAIHV
GARL#

>PPATMP010150_3/ PpAamy4
MRIASAVLVSFFLALASGQHNPNFWGGRTGIVHLFEWKFSDVADECERFLAPRGYAGVQLSPVNENVIIP
NRPWWERYQPISYVLTTRSGNENDFLDMSRRCNAVGIRIYVDVILNHMSADNNPSDGTAGNYADTSARLW
PAVPFGPNDFNPRCSIYNWDDPVEIRNCELVGLHDLNQATEHVRSMLVNFLNHLVQLGAAGFRVDAAKHV
WPHDLEEIYNRVNNLNTDFGFAPGSRPYIYQEVIELGGSPIRYEYTPLGSITEFKFSAEIGRVFRGYDQL
RWLRNWGPEWGFVPSNVALVFVDNHDNQRGHGAGGENILTYKQPRQYKMATAFTLAWDYGTVRLMSSFAF
NHGDQGPPQDANENIISPSINPDNTCGNGWVCEHRWRQIYNMIGFANQVQGTGVNDFWDDGGNQIAFCRG
DQGFIAFNNDQHNFSHTLQTCLPAGTYCDIISGENTGQGCSGVTITVNPDQTAHIVIPVDHYDGMIAIHR
GAASRVN#

>PPATMP010150_4/ PpAamy5
MRIASAVLVLCCLALASGHHWPNFWGGRTGIVHLFEWKFKDVADECERFLAPKGYAGVQLSPVNENVIIP
NRPWWERYQPISYVLTTRSGNENDFLDMSRRCNAVGIRIYIDVLLNHMSADNHPSDGTAGNWADTSQRWW
PAVPFGPNDFNPRCSIYNWDDPVEIRNCELVGLHDLNQGSEHVRSMLVNFLNHLVQLGAAGFRVDAAKHV
WPHDLEEIYNRVNNLNTDFGFAPGSRPYIYQEVIDLGGRPFRHEYTPLGAITEFRFSAEIGRVFRGYDQL
RWLRNWGPEWGFVDSRVALVFVDNHDNQRGHGAGGENVLTYKQPRQYKMATAFTLAWDYGTVRLMSSFAF
NHGDQGPPQDANENIISPSINPDNTCGNGWVCEHRWRQIYNMIGFANQVQGTGVNDFWDNGGNQIAFCRG

DQGFIAFNNDQHNFSHTLQTCLPAGTYCDIISGENTGQGCSGLTITVNWDQTAHIVIPVDHYDGVIAIHR
GPASRLN

>PPATMP010151_1/ PpAamy6
MLEAQFDPYFLPGRSVIVHLFEWKFADIAKECEEYLGPNGFGGVQVSPVNEVLVTPLGAWWERYQPVSYA
IIGRSGDELDFMDMVKRCYDAGVRIYVDVVFNHMAAGPGEIIGTGGSVVYPDERLYPHVPYGPEDFNDDC
LIEDYQDTVQVRDCALVSLPDLNQKSDNVKRNVIEFLDRLIDYGVAGFRADACKHMWPDDIKFLFGNTKN
LSIEFGFPPNARPFLYQEVIDLGQEPISKNEYITNGAITEFLFSAEIGDIFRNKKLKELMNWGTDERFLR
SDRALVFVENHDNERGHGAGGENILTYKDGKRYMMAVLFAIAHPYGIVRIMSSYDFANSDQGPPAKPDLE
IISPVFNEKGLCTNGWICQHRWPEVAAMVKFRNIVGERGVMHWADNGEQQFAFCRGDLGFVAFNGYTRSN
LESDVKVCLPPGIYCDVISGSVGELGCTGLEVVVNDAGYAKVFIPADSPSGVLAIHLGSVFIPSK#

>PPATMP010151_2/ PpAamy7
MNILIALAVIVTSLSVRAQFNPHYIDNRNVIVHLFEWKWNDIASECKNFLGPNGFAGVQVSPVNENWVSP
ERAWYERYQPISYKLTTRSGTQEKFASMVETCRQSGVRIYVDVVFNHMASGSLDTILVGTAGSEAYPQVF
DYPAVPFTKADFHPDCTITDYQDVYQIRNCQLSSLRDLNQTSPYVREKILGFLNQLVDLGVAGFRVDAAK
HMDPQDLRYIYRTIKRLNKDSGFRGTDKAFIVQEVIDLGGEALSSREYTSFGVVTEFKASDELGKAFRGK
YPLADLRHWGPLYGLLPPNRALVFVENHDNERGHGAGGENILTYKNGKIYTMAVAFNLAHSYGIPRIMSS
YEFNDPSQGPPHDDSNNILSPEFSSNGESCTNGWVCQHRWLAIRNMVKFRNVVRKTSVTLWYDNGSNQIA
FSRGTRGFIAFNGDKVDFSAKVRTYLRSGTYCDVISGEKNGNSCTGKTVNVRRSEVAVIIEADDPYGVIA
IHAESKL#

>PPATMP010151_3/ PpAamy8
MNLSAILIFTFLTLAHGQFDPNYAPGRNVIVHLFEWKWVDIAEECERFLGPNGFAGVQVSSPAENAIVTV
LGFNRPWWERYHIMSYILETRSGNELEFADMVRRCNAVGVRIYPDAVFNHMGSLPNMTGTGGSTADGVIR
TYPEIPYNATHFHIPCPIVDYQNSDNVRNCQLLGAPDLNHTLEYVRNKIAGYLDHLITLGVAGFRVDSAK
HIWPSDSQDIYGRVSNLNTDHGFADNSKPYFYHEVIDVGGEAVSKYDYTGLGGVTEFLLPYYIGITFRGN
RSLDTLQNWGEPWGFLPSDRGIAFVENHDNERGTSGRDILTYKDGKPYRMAVAFNLAHPYGITRIMSSFY
FSYDNIDQSPPMDDNQNIISPSINPDGSCGNGWVCQHRWHQIYNMVKFKNVAGTAPLSNWWSNGNNQIAF
ARSGKGFVAFNNEGSDMDVILQTSLPAGTYCDVISGSVSGTTCSGKTVIVQVDGMANIVIGSAEEDGVLA
IHVGAKL#

Maltase
>PPATMP010760_1/ PpAgluB2
MRIFSVTVLCLCYSEILGDLDWWKTANIYQIYPRSFKDSNGDGIGDLNGITERLPYLKDLGIHAVWLSPI
FKSPMKDFGYDISDFYDIQPEYGTIQDFEHLLKVAHDLNIRVLLDFVPNHSSDENEWFVKSANRDPDFED
FYVWHPGKEDPSDATKKLPPSNWLSVFRGSAWRLHEGRGEYYLHQFVYQQPDLNYRNPKVVDQMKDVLRF
WLSKGVDGFRIDAVPHLYEIAPENGQYPDEPKSGNSDDPDNSAYLDHIYTQNQPETVQMVYQWREVLDEF
TNRDQNTRLMLTEAYSDIDILMTYYGDGVKNGSHVPFNFFLITQLNNDSNAIDFHKTIRLWLDKLPQDCI
ANWVIGNHDQRRVASRFGTYRIDVINMILNTLPGVSITYNGEEIGMTDVFLTWEETVDPAACNSDPQHYQ
EFSRDPERTPFXXXXXXXXXXXXXXXXXXXXXXXXXXXXXXXXXXXXXXXXXXXXXXXXXXXXX
XXXXXXXXXXXXXXXXXXXXXXXXXXXXXXXXXXXXXXXXXXXXXXXXXXXXXXXXXXXXXXXXXXXXXX
XXXXXXXXXXXXXXXXXXXXXXXXXXXXXXX

> PPATMP010760_2/ PpAgluB3
MRIFSVTILCLCYSGILGDLDWWKTANVYQIYPRSFKDSNGDGIGDLNGITERLPYLKDLGIHAAWLSPI
FKSPMVDFGYDISDFRDIQPEYGTLQDFDRLLKIAHDLNIKILLDLVPNHSSDQHEWFVKSANRDPDFVD
YYVWHPGKDDPNDPTKKLPPSNWLSDFRGSAWKWHEERGEYYLHQFAYQQPDLNYRNAKVVDEMKDIIRF
WLNRGVDGFRIDAVLCLFEAEPVDGVYPDEPKSGNTDDPDNPKYLTHIYTQNQPETVEMVYQWREVFEEF
TNADQNTRLMLTEAYSDIDILMTYYGDGVRKGSHVPFNFFLLTHIKNDSNAIDFRKAIQIWLDKLPEDCV
ANWVLGNHDQRRVASRFGTHRIDIINMILNTLPGISITYNGEEIGMTDVFLTWEETVDPPACNSNSEIYD
KFSRDPARTPFQWDDSTSAGFSTNTKTWLPVSPLYKEVNVKVERSSRRSHYKVYRKLLQLRRTPTLQKGN
VETRTHGQNVLSITRSHPGEDTFITVVNIGPDHEMIDLRELFSGRLTFHIVSVYSKRHEGDPIRGPILDL
DPHEGVILKGFPXXXXXXXXXXXX

> PPATMP010760_3/ PpAgluB4
MRIFSVTVLCLCYSGILGDLDWWKTANIYQIYPRSFKDSNGDGIGDLNGITERLPYLKDLGIHAVWLSPI
FKSPMADFGYDISDFYDIQPEYGTIQDFEHLLKVAHDLNVRVLLDFVPNHSSDQNEWFIKSANRDPDFVD
YYVWRDWKGDPSDPTKRLPPTNWISLFRGSAWKWHEGREQFYLHQFAYQQPDLNYRNPKVVAQMKDVLRF
WLNKGVDGFRIDAVWTVFEIAPDSDGNFPDEPLSGNSNDPDDHGYLNHIYTQNQPETVDMVYQWRELLEE
FTSKDQNTRLMLTEAYADVDIVMTYYGDGVRKGSHVPFNFNMITDLHNASNAVDFHRLIRVWLDKLPKDC

VANWVIGNHDQKRVGSRFGTHRIDVINMMLNTLPGISITYNGEEIGMTDVFMTWETVDPAACNSNPQIYE
GLSRDPARTPFQWDDSTSAGFSTNPKTWLPVSPLYKEVNVKVERSSPRSHYKIYRKLLQLRRTPTLQKGN
VETRTHGENVLSITRSLPGEDTYITVVNIGPDREVVDLRELFSGRLTFHIISLTSKRREGVAVRGPILDL
DPHEGVILKGSAFHXXXXXXXXXXXXXXXXXXXXXXXXXXXXXXXXXXXXXXXXXXXXXXX
XXXX

> PPATMP010760_4/ PpAgluB5
MKTFLLWVLCWNHICGSQSDLDWWRTANFYQIYPRSFKDSNGDGIGDLNGITERLPYLKDLGIHAVWLSP
IFKSPMKDFGYDISDFYDIQPEYGTLQDFDRLIRIAHDLNLKILLDFVPNHSSNENEWFEKSANRDPDFE
DFYVWHPGKDDPSDPIKKLPPSNWRSVFRGSAWTYHNKRGEYYLHQFAHFQPDLNYRNPKVVDTMKGVLR
YWLDKGVDGFRVDAVPNLFEILPQNGHYPDEPLSSLTEDEDSFDYLQHIYTMDQPETVEMLYQWREVLDV
YTARDGNMRTMLSESDSEISILMTYYGDGIRNGSHVPFNFKLLFNLNKNSNAIDFRNTIHIWLDNLPEGC
VANWVIGNHDRNRVASRFGSFRIDLINIIINTLPGISITYYGEEIGMMDVFVTWEDSVDPAACNSDPDHY
LEFSRDPARTPFQWDDSTSSGFSTNPKTWLPVSPLYKEVNVKVERSSPRSHYKVYRKLLQLRRTPTLQKG
NVETRTHGQNVLSITRSHPGEDTFITVVNIGPDHEMVDLRELFSGRLIFYIVCVSSTRREGDVVRAPILN
LDPFEAIILKGAIQYXXXXXXXXXX
